# The RNA-bound proteome of MRSA reveals post-transcriptional roles for helix-turn-helix DNA-binding and Rossmann-fold proteins

**DOI:** 10.1038/s41467-022-30553-8

**Published:** 2022-05-24

**Authors:** Liang-Cui Chu, Pedro Arede, Wei Li, Erika C. Urdaneta, Ivayla Ivanova, Stuart W. McKellar, Jimi C. Wills, Theresa Fröhlich, Alexander von Kriegsheim, Benedikt M. Beckmann, Sander Granneman

**Affiliations:** 1 Centre for Synthetic and Systems Biology, University of Edinburgh, Edinburgh, EH9 3BF UK; 2 IRI Life Sciences, Humboldt University Berlin, 10115 Berlin, Germany; 3 Cancer Research UK Edinburgh Centre, Institute of Genetics and Molecular Medicine, University of Edinburgh, Edinburgh, EH4 2XR UK

**Keywords:** RNA, Bacteria, Pathogens, Proteomics

## Abstract

RNA-binding proteins play key roles in controlling gene expression in many organisms, but relatively few have been identified and characterised in detail in Gram-positive bacteria. Here, we globally analyse RNA-binding proteins in methicillin-resistant *Staphylococcus aureus* (MRSA) using two complementary biochemical approaches. We identify hundreds of putative RNA-binding proteins, many containing unconventional RNA-binding domains such as Rossmann-fold domains. Remarkably, more than half of the proteins containing helix-turn-helix (HTH) domains, which are frequently found in prokaryotic transcription factors, bind RNA in vivo. In particular, the CcpA transcription factor, a master regulator of carbon metabolism, uses its HTH domain to bind hundreds of RNAs near intrinsic transcription terminators in vivo. We propose that CcpA, besides acting as a transcription factor, post-transcriptionally regulates the stability of many RNAs.

## Abstract (web summary)

RNA-binding proteins play key roles in controlling gene expression in many organisms. Here, Chu et al. identify hundreds of RNA-binding proteins in methicillin-resistant *Staphylococcus aureus*, and show that a major transcription factor uses its helix-turn-helix domain to bind RNAs near intrinsic transcription terminators.

## Introduction

The rapid spread of highly virulent and methicillin-resistant *Staphylococcus aureus* strains (MRSA) is causing major health-care problems worldwide and is becoming increasingly difficult to treat^1^. *S. aureus* is such an effective pathogen because it expresses numerous virulence factors that facilitate its ability to colonise, evade immune defenses and transmit to other hosts^1^. Its capacity to very rapidly adapt to stress caused by environmental insults, such as alteration in host temperature because of an inflammatory response, enables it not only to survive in hostile conditions but also to persist within the host^1^. The latter condition can promote resistance to antimicrobials and the host immune system, leading to chronic infections^2^. To achieve such rapid adaptation, *S. aureus* remodels its transcriptome within minutes of stress imposition^3^.

Transcription factors were thought to be mainly responsible for directing this process by controlling gene expression at the DNA level; however, it has become evident that a substantial amount of regulation occurs post-transcriptionally: RNA-binding proteins (RBPs), are now recognised as key players in controlling adaptive responses in pathogenic bacteria^4,5^. By directly binding to mRNAs, RBPs can shape gene expression profiles by modulating mRNA translation and/or degradation rates.

Although RBPs clearly play a key role in regulating adaptive responses, relatively few RBPs have been identified and characterised in *S. aureus*. Recent in vivo high-throughput proteomic studies have identified hundreds of RBPs (RNA-binding proteome (RBPome)) in diverse organisms^6–11^. Collectively, these studies revealed a surprisingly large number of proteins that previously did not have any known links to RNA metabolism, nor did they contain recognisable RNA-binding domains (RBDs). This included many metabolic enzymes that moonlight as RBPs. These may represent a “shortcut” that directly links the response to nutrient availability with post-transcriptional regulation of gene expression. As metabolic pathways are frequently targeted for drug development, the RNA-binding enzymes may be promising candidates.

Thus far, RBPome studies uncovered RBPs for Gram-negative bacteria, many of which do not have conserved homologues in Gram-positive bacteria, such as *S. aureus*. Vice versa, *S. aureus* also expresses many proteins that are not conserved in *E. coli*. Therefore, in this work, we applied silica- (complex capture (2C)^12^) and organic extraction-based (phenol–toluol extraction (PTex)^13^) approaches to two clinically relevant *S. aureus* strains to obtain a global overview of *S. aureus* RBPs. We biochemically verified our findings in vitro as well as in vivo using ultraviolet (UV) cross-linking and cDNA analyses experiments (CRAC^14^). Our RBPome protocols are widely applicable, and our work implies that the role of HTH domain proteins in regulating gene expression has been considerably underestimated.

## Results

### 2C and PTex reliably capture *S. aureus* RBPs

**Fig. 1:**
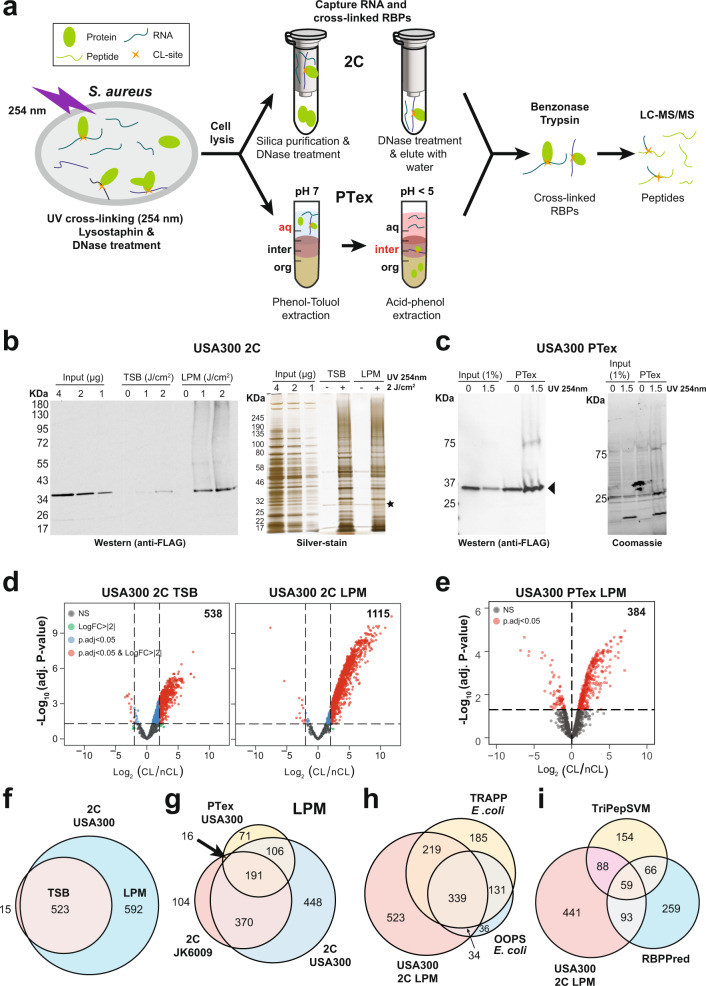
RBPome capture in *S. aureus*. **a** *S. aureus* 2C and PTex RBPome capture workflows. See ‘Methods’ section for a detailed description of the approaches. CL cross-linked, 2C complex capture, PTex phenol–toluol extraction, aq aqueous, inter interphase, org organic. **b** Western blot and silver staining analysis of the USA300 RNase III-HTF 2C experiments. The UV irradiation doses used (J/cm^2^) are indicated. RNase III-HTF (black triangle) was detected using anti-FLAG antibodies. ‘Input’ indicates µg of total lysate loaded on the gel. The black asterisk indicates the Benzonase used to degrade the RNA. Non-cross-linked cells were used as negative controls. TSB tryptic soy broth, LPM low phosphate medium pH 7.5. **c** Western blot results and Coomassie brilliant blue staining of the USA300 RNase III-HTF PTex experiments, performed with the indicated UV intensities (J/cm^2^) in LPM medium. One percent of the total lysate was loaded for comparison. Original images for the experiments shown in **b**, **c** are provided in the Source data. **d** Volcano plots of proteins enriched in TSB (left) and LPM medium 2C data (right) (*n* = 6; two independent biological replicates, three technical replicates). *P* values were generated by empirical Bayes moderated *t* test in limma and adjusted by Benjamini–Hochberg method. Proteins with a log-fold change >2 and a −log_10_ adjusted *P* value of ≥1.3 (indicated with dashed lines) were considered significantly enriched (red dots). The total number of proteins significantly enriched in the cross-linked samples is indicated. **e** As in **d** but now for the PTex results (3 biological replicates) from cells grown in LPM medium. An adjusted *P* value threshold of 0.05 was used to select proteins enriched in the cross-linked samples. **f** Overlap of significantly enriched RBPs identified in TSB and LPM 2C data. **g** Overlap of JDK6009 and USA300 RBPs identified by 2C and PTex in cells grown in LPM medium. **h** Overlap of USA300 RBPs identified by 2C with published *E. coli* OOPS and TRAPP RBPome data sets. **i** Overlap of USA300 RBPs identified by 2C with RBPs predicted by TriPepSVM and RBPPred. TRAPP total RNA-associated protein purification, OOPS orthogonal organic phase separation.

To identify the RNA-bound proteome in *S. aureus*, we applied silica (2C^12^) and organic extraction-based (PTex^13^) approaches (Fig. 1a). We UV irradiated cells in two different growth media: brown coloured tryptic soy broth (TSB) and a clear low phosphate medium (LPM). Based on previous work^15^, we predicted that the LPM medium would absorb less UV, resulting in higher cross-linking yields. For the experiments in LPM medium, we first rapidly filtered cells grown in TSB and transferred them to LPM medium for 15 min before cross-linking (see ‘Methods’ for details). We analysed the RNA-bound proteome of two clinically relevant strains: the vancomycin sensitive JKD6009 and the highly virulent community-acquired USA300 MRSA.

The 2C protocol^12^ had not yet been applied to bacteria, therefore we first optimised the conditions by testing different lysis approaches and UV irradiation intensities (detailed in Methods). We generated strains in which a HIS6-TEV-3xFLAG (HTF) tag was fused to the C-terminus of RNase III^16^, enabling us to monitor the recovery of the RNA cross-linked ribonuclease during the purification steps by Western blotting. This revealed that high UV doses (up to 2 J/cm^2^) were required to obtain sufficient RNase III cross-linking yields (Fig. 1b). This, however, did not result in major RNA degradation (Supplementary Fig. 1a), suggesting that only a fraction of UV penetrates the cells. Analysis of total cell lysates revealed that protein steady-state levels did not significantly change during the 15-minute incubation in LPM medium (Supplementary Fig. 1b). Most of the proteins cross-linked to RNA more efficiently when the cells were grown in LPM medium compared to TSB (Fig. 1b and Supplementary Fig. 1b–d). Hundreds of proteins were enriched in the TSB and LPM cross-linked samples, but we always detected a larger number of proteins in the LPM 2C data (Fig. 1d). We conclude that the increase in cross-linking efficiency is, at least in part, a direct result of the colour of the LPM medium. However, it is very likely that many of the changes in cross-linking efficiency were the result of the cells adapting to the nutrient-poor LPM medium.

Cross-linking of RNase III in the JKD6009 strain was inefficient in TSB (Supplementary Fig. 1c) and therefore we only analysed the JKD6009 RBPome in LPM medium conditions (Fig. 1g and Supplementary Fig. 1e, f).

We next applied the PTex protocol to the USA300 RNase III-HTF strain grown in LPM medium. RNase III-HTF was also enriched by PTex (Fig. 1c), although background levels were higher compared to 2C (Fig. 1b). When applying the same cut-offs used for the 2C analyses (log_2_ fold change >2 and adjusted *P* value <0.05), 197 proteins were obtained. However, because *S. aureus* PTex data showed higher background levels, we used less stringent criteria for selecting enriched proteins by only considering the proteins with enrichment *P* values of ≤0.05. This yielded 384 proteins (Fig. 1e).

There was a very high correlation between the protein signal intensities in the samples, demonstrating the reproducibility of the results (Supplementary Fig. 2). Almost all the proteins identified by 2C in the TSB medium data were also detected in the LPM medium data (Fig. 1f). In addition, there was a strong overlap between the 2C and PTex data (Fig. 1g), demonstrating that the two methods are highly complementary. All the proteins significantly enriched in the 2C and PTex RBPome analyses can be found in Supplementary Data 1.

Homologues of over half of the RBPs identified in the LPM 2C data were also found in existing *E. coli* RBPome data sets^11,17^, implying that many conserved RBPs bind RNA in both Gram-negatives and Gram-positives (Fig. 1h). We also compared our data with RBPs predicted in silico by the TriPepSVM and RBPPred algorithms (Fig. 1i and Supplementary Fig. 1f^15,18,19^). The TriPepSVM and RBPPred predictions did not show a strong overlap but together they recovered over 300 RBPs identified by 2C in LPM samples. The TriPepSVM method generally agreed best with the 2C LPM RBPome data sets generated from both strains.

**Fig. 2:**
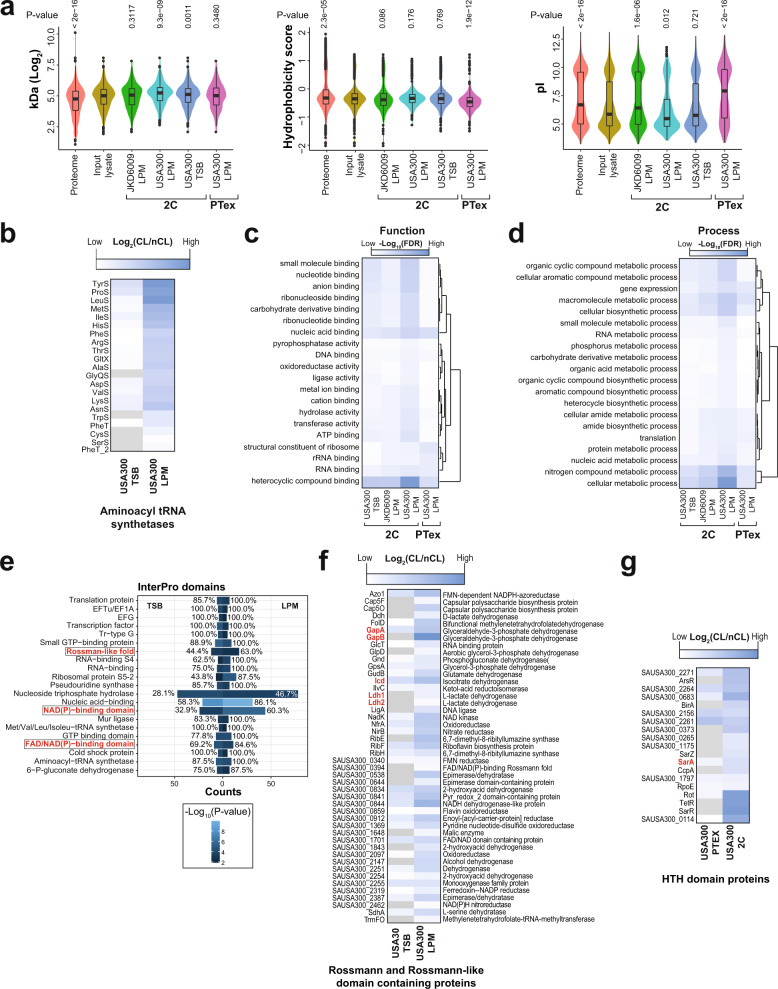
2C and PTex data are highly enriched for nucleic acid-binding proteins and metabolic enzymes. **a** Comparison of the molecular weights (kDa), hydrophobicity scores and isoelectric points (pI) of USA300 proteins in the whole proteome (3062), proteins detected by mass-spectrometry in cell lysates (input lysate, 1702), 2C RBPome data (JKD6009 in LPM, USA300 in LPM and TSB; 681, 1115 and 538 separately) and PTex RBPome data (USA300 in LPM, 384) (box plots centre: median; minima/maxima: the minimum/maximum value in the data set excluding outliers; lower/upper hinges: the first/third quartiles; upper/lower whisker: from the hinge to the largest/ smallest value at most 1.5× IQR (inter-quartile range) from the hinge; outliers: data beyond whiskers). *P* values (numbers above each plot) were calculated using a two-tailed Wilcoxon test using the input lysate data as the reference group. **b** Aminoacyl tRNA synthetases enrichment in the 2C RBPome data. **c**, **d** Gene Ontology (GO) enrichment analysis of Molecular Function and Biological Processes using STRINGdb. Heatmap shows the top 20 GO-terms identified in four different *S. aureus* RBPome data sets. **e** InterPro domain overrepresentation analysis comparing 2C RBPome from TSB samples (left) with LPM samples (right). *P* values (unadjusted) were generated as described in the Methods section. The percentage indicates the fraction of all proteins with the corresponding InterPro domains in USA300 that were identified with 2C. The lighter the blue colour, the higher the significance. **f**, **g** Rossmann/Rossmann-like and HTH domain proteins that were enriched in 2C and PTex RBPome data. The colours indicate the log_2_ fold change in protein intensities between cross-linked (CL) and non-cross-linked (nCL). The darker blue the colour, the higher the enrichment in the cross-linked sample.

In some data sets, the average molecular weight of the isolated RBPs was slightly higher compared to total lysates (Fig. 2a left violin plot). Note that the LC/MS data obtained from cell lysates also appeared to have a similar bias for higher molecular weight proteins, so the slight bias observed in the RBPome data may be associated with technical limitations of the LC/MS approach (Fig. 2a left violin plot). The PTex data was also more enriched for hydrophilic proteins (Fig. 2a middle violin plot). Previous RBPome studies revealed that oligo-d(T) captured RBPs generally have basic isoelectric points (pIs) compared to the whole proteome^8^. However, oligo-d(T) independent methods such as PTex and XRNAX (protein-X-linked RNA eXtraction), generally showed bias towards acidic proteins^7,13^. Although our 2C data did not show a bias in the hydrophobicity of the proteins, acidic proteins were also preferably recovered (Fig. 2a, right violin plot). The *S. aureus* RBPs purified with PTex were more basic compared to the input lysate proteins (Fig. 2a, right violin plot). Most of the *S. aureus* aminoacyl-tRNA synthetases were enriched in the 2C data (Fig. 2b and Supplementary Data 1), indicating that the method effectively recovers RBPs that bind relatively short RNAs.

We next asked what Gene Ontology (GO) terms were enriched in the putative RBPs using the STRINGdb^20^ R package (Fig. 2c, d). We focused our analyses on the 2C data as we consistently obtained a higher number of proteins with this method, allowing us to carry out a more meaningful statistical comparison between the individual data sets. The *S. aureus* 2C data were strongly enriched for nucleic acid binding and RBPs, including factors involved in translation (rRNA-binding and ribosomal proteins; Fig. 2c). In addition, metabolic enzymes involved in nucleoside/nucleotide metabolism and biosynthesis were also very abundant (Fig. 2d). This is consistent with previous studies demonstrating that many metabolic enzymes can also bind RNA (reviewed in^21^). When we classified the proteins based on their domain composition using InterPro, we found that many domains associated with RNA-binding, including ribosomal protein domains (S4, S5) and translation factors (EFTu, EFG, tRNA synthetases), were abundant in our data (Fig. 2e). We did not observe major differences between the domain analyses performed on the data from the two media but noticed a higher recovery of proteins with nucleoside triphosphate hydrolase activity and nucleic acid binding activity in the LPM data (Fig. 2e)

We conclude that both methods successfully identified hundreds of RBPs in *S. aureus*.

### The majority of *S. aureus* HTH DNA-binding and Rossmann(-like) fold domain proteins bind RNA

A surprising observation was the sheer abundance of Rossmann fold or Rossmann-like fold proteins (NAD(P) and ATP-binding) that were enriched in the 2C data set (Fig. 2e, f). This included many proteins with FAD and/or NAD-domains that are required for oxidation and reduction reactions and play an important role in a wide variety of metabolic processes. Interestingly, these proteins appeared to cross-link to RNA more efficiently/frequently in LPM medium. For example, almost 85% of all the annotated FAD/NAD-binding proteins in USA300 were recovered from LPM samples (Fig. 2e, f). Several NAD-binding proteins have been described to bind to RNA in eukaryotes, GAPDH being a classical example^17,22^. Our results suggest that almost all *S. aureus* FAD/NAD-binding proteins can bind RNA. GAPDH isoforms (SAUSA300_RS08910 (GapA) and SAUSA300_RS04080 (GapB) in USA300) were also recovered in our data sets (Fig. 2f and Supplementary Data 1), demonstrating that the RNA-binding activity of this enzyme is conserved in *S. aureus*. We also recovered three other NAD-dependent enzymes known to bind RNA in eukaryotes. This included two L-lactate dehydrogenases (Ldh1 and Ldh2), that, like GAPDH, bind AU-rich elements (AREs) in human cells^23^. Isocitrate dehydrogenase (Icd) was also enriched in the LPM 2C data (Fig. 2f). In addition, we identified 21 uncharacterised *S. aureus* proteins with NAD-binding domains that cross-linked to RNA (Fig. 2f).

Many DNA-binding proteins were also enriched in PTex and 2C, in particular proteins containing Helix-Turn-Helix (HTH) DNA-binding domains (Fig. 2g). This was unexpected since we included DNase treatment steps in the 2C and PTex protocols (see ‘Methods’). On the other hand, the *S. aureus* HTH-domain protein SarA was previously shown to interact with RNA^24^, suggesting that HTH-domain proteins can bind both RNA and DNA.

Collectively, these data imply that many Rossmann fold and HTH DNA-binding proteins could also have a role in RNA metabolism.

### Validation of the 2C data using CRAC

**Fig. 3:**
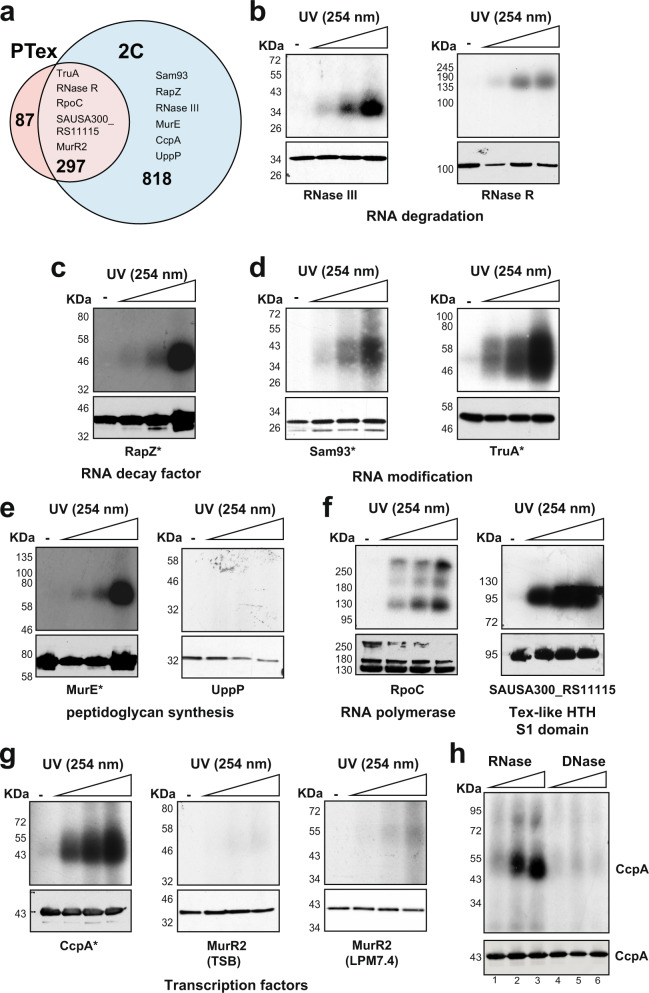
PTex and 2C reliably detect RNA-binding proteins in *S. aureus*. **a** Overlap between PTex and 2C RBPome data sets generated from cells grown in LPM medium. **b**–**g** UV cross-linking analysis of known (**b**) and predicted RBPs (**c**–**g**). USA300 strains expressing HTF-tagged fusion proteins were irradiated with different UV intensities. HTF-tagged proteins and cross-linked RNA were purified under highly stringent and denaturing conditions (see ‘Methods’ for details). After a mild RNase digestion and 5’ ^32^P labelling of the cross-linked RNA, samples were resolved by NuPAGE, transferred to nitrocellulose and cross-linked ribonucleoprotein complexes were detected by autoradiography (upper panel). Western blot analysis with anti-TAP antibodies was performed to visualise the (free) proteins (lower panel). Asterisks indicate signals that were generated from overnight exposures of gels for autoradiograph. Other exposures were 3 h or less. **h** CcpA UV preferentially cross-links to RNA. Cells expressing CcpA-HTF were UV-irradiated and cross-linked CcpA-nucleic acid complexes were incubated with an increasing amount of RNase A/T1 (lanes 1–3) or RQ1 DNase (lanes 4–6). The original pictures from one replicate of the CRAC experiments shown in **b**–**h** are provided as a Source data file.

To validate some of our findings, we epitope-tagged 11 USA300 *S. aureus* proteins with a codon-optimised version of the HTF^16^ and performed CRAC UV cross-linking experiments^14^ to confirm that the proteins directly bind to RNA. Proteins were selected based on their predicted function as well as whether they were uniquely identified in 2C or both PTex and 2C (Fig. 3a). RNase R and RNase III, two well-characterised RNA decay factors, were included as positive controls (Fig. 3b). Proteins were tandem affinity purified under highly denaturing conditions^25^, cross-linked RNAs were partially digested with RNases and radioactively labelled. Radioactive complexes were resolved by Bis-Tris protein gels and detected by autoradiography. The positive controls robustly cross-linked to RNA even at lower UV intensities (Fig. 3b). We also tested another RNA decay factor (RapZ; Fig. 3c), RNA modification enzymes (TruA and the putative SAM methyl transferase Sam93 (SAUSA300_RS09320); Fig. 3d) and two peptidoglycan synthesis enzymes (MurE and UppP; Fig. 3e). As DNA-binding proteins were frequently detected in our data, we tested several DNA-binding proteins, including two HTH-domain proteins (CcpA and SAUSA300_RS11115; Fig. 3f, g). Under the tested conditions, only UppP did not detectably cross-link to RNA (Fig. 3e). UppP is therefore likely a false-positive, although we cannot exclude the possibility that the tag interfered with its RNA-binding function. The MurR2 (SAUSA300_RS12510) transcription factor weakly, but reproducibly, cross-linked to RNA in both TSB and LPM medium in vivo in a UV-dose dependent manner (Fig. 3g).

While these CRAC experiments provide compelling evidence that the tested DNA-binding proteins also directly bind RNA in vivo, we could not exclude the possibility that some of the signal we obtained was the result of the proteins cross-linking to DNA^26^. To investigate this further, we incubated in vivo cross-linked CcpA with increasing concentrations of RNase or DNase and purified the cross-linked ribonucleoprotein (RNP) complexes using the CRAC approach as described above. In the presence of very low nuclease concentrations (Fig. 3h, lanes 1 and 4), the radioactive signal in the gel was diffuse, indicative of protein cross-linked to long nucleic acid strands. When we increased the RNase concentration, a stronger and more discrete radioactive signal was detected in the region of the gel where the free protein migrates. This implies that the treatment shortened the cross-linked nucleic acids (Fig. 3h, lanes 2 and 3). In contrast, increasing the DNase concentration did not noticeably change the signal (Fig. 3h, lanes 5 and 6). We therefore conclude that our UV irradiation conditions favour CcpA cross-linking to RNA.

Collectively, these data provide support for our hypothesis that HTH-type DNA binding proteins also bind RNA in vivo. Additionally, our CRAC validation experiments demonstrate that 2C and PTex reliably identify RBPs in *S. aureus*.

### The transcription factor CcpA is a global RBP that preferentially binds RNA near 3’ ends

**Fig. 4:**
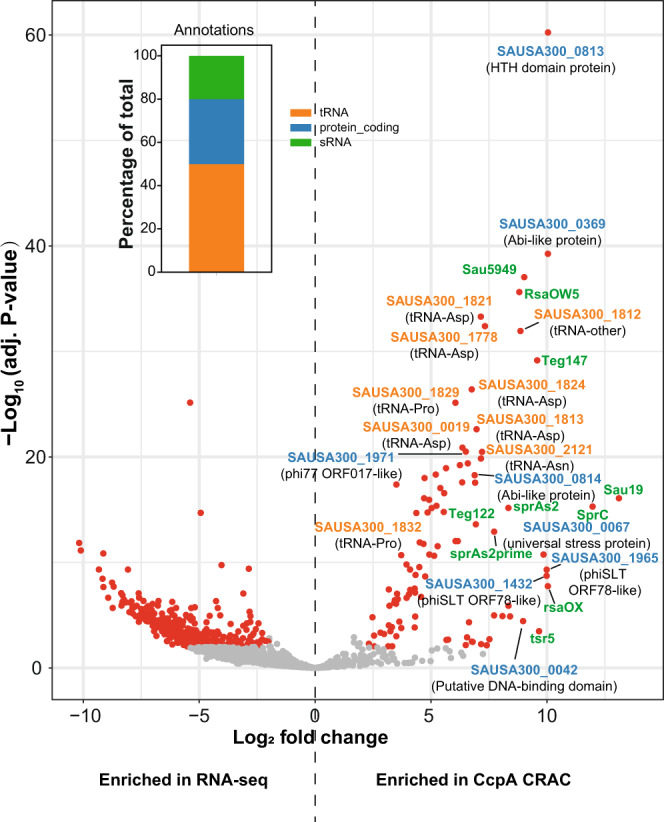
CcpA CRAC data are highly enriched for tRNAs, mRNAs and sRNAs. Volcano plot showing genes that are enriched in the CcpA CRAC data (right half of the volcano plot) relative to RNA expression levels obtained from RNA-seq data (left half of the volcano plot). For several transcripts that were highly enriched in the CRAC data we included the gene names. Gene names coloured orange encode for tRNAs; Blue: mRNAs; Green: sRNAs. The bar plot embedded in the volcano plot the percentage of genes enriched in the CcpA CRAC data that map to tRNAs, sRNA and mRNAs. Benjamini and Hochberg adjusted *P* values were calculated using DESeq2^28^ for six CRAC samples (two independent biological and three technical replicates each) to three biological replicate RNA-seq data sets generated from cells that were incubated with LPM pH 7.5 medium for 15 min.

We next investigated the interaction between HTH-type proteins and RNA in more detail, focussing on the well characterised HTH protein, CcpA. To identify the in vivo RNA substrates, we purified the RNAs cross-linked to CcpA under highly stringent and denaturing conditions using the CRAC protocol^14,27^. Following a limited RNase digestion, cross-linked RNA fragments were deproteinized and converted into NGS sequencing libraries. As negative controls, we used UV cross-linked cells from the parental strain. Two biological replicate experiments were performed, each with three technical replicates. The level of background noise in our CRAC experiments was low, as judged by the very small number of mapped reads in the negative control samples (Supplementary Fig. 3a). The CRAC experiments generated highly reproducible results (Supplementary Fig. 3b, c) and CcpA bound to hundreds of transcripts in our data (Supplementary Data 2). We subsequently used DESeq2^28^ to identify CcpA-bound transcripts that are highly enriched relative to their expression levels (Supplementary Data 3). Most of the significantly enriched transcripts were tRNAs, followed by protein-coding genes and small RNAs (sRNAs) (Fig. 4, bar plot). Interestingly, many of the highly enriched mRNAs are encoded on pathogenicity islands and/or phage-related genomic islands (Supplementary Data 3 and Fig. 4, volcano plot). This included a HTH domain protein of unknown function (Fig. 4, SAUSA300_0813).

**Fig. 5:**
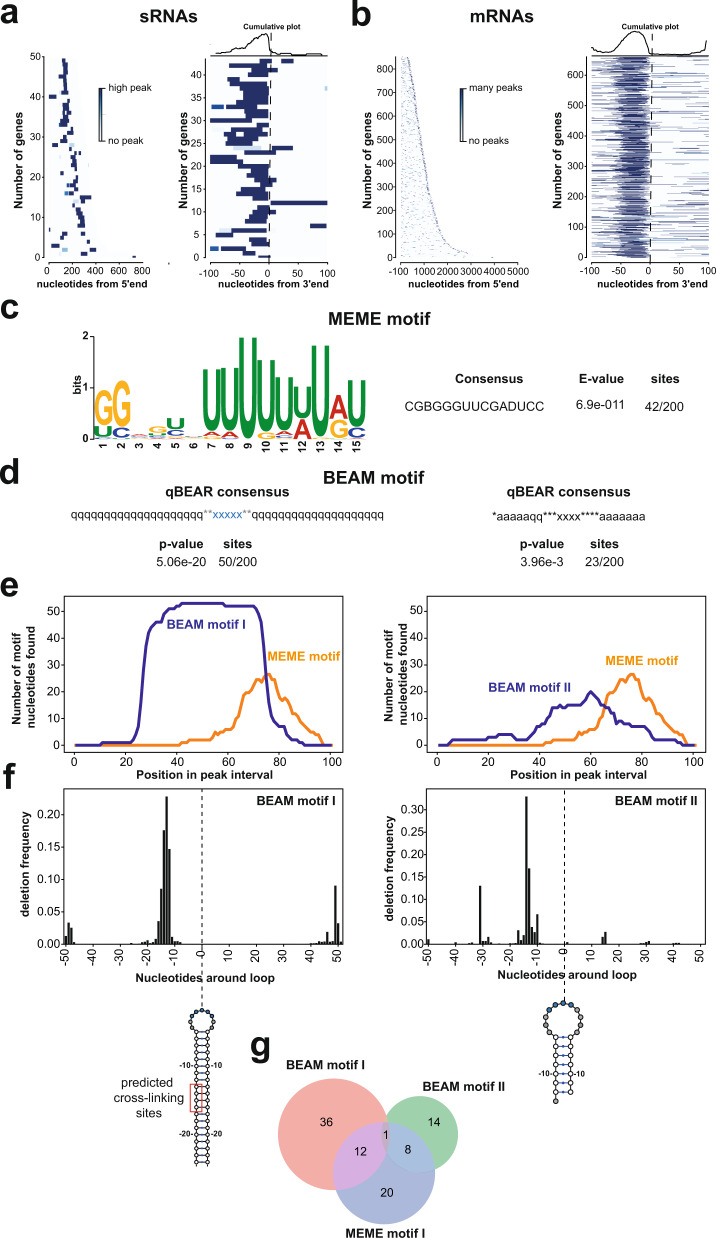
The HTH-type DNA-binding protein CcpA binds hundreds of RNAs in vivo. **a**, **b** Distribution of highly enriched CcpA binding sites (FDR ≤ 0.01) in USA300 sRNA (**a**) and mRNA (**b**) transcripts. The darker the blue signal in the heat map, the higher the number of reads found in a peak (maximum value was set at 10% of total peaks found in a transcript). The first heat map in **a**, **b** shows the peak distribution in all the identified sRNA (**a**) and mRNA (**b**) transcripts sorted by length (shortest top, longest bottom). The second heat map shows the distribution of the peaks around transcript 3’ ends. The total number of peaks in this region is indicated by the cumulative plot above the heat map. **c** The top 200 enriched CcpA CRAC peaks were analysed using the MEME suite^31^. The motif consensus sequence and the *E*-value of the motif are also provided. The motifs identified by MEME for tRNAs can be found in Supplementary Fig. 5a. **d** Enriched RNA structural motifs identified by BEAM (BEAr Motifs finder)^30^ in the top 200 enriched CcpA CRAC peaks. The two most highly enriched structural motifs are shown. qBEAR: quick Brand nEw Alphabet for RNAs. Encoding: q: stem structures that are longer than 10 nucleotides; a: stem structures between 6 and 9 nucleotides; x: 3–9 nucleotide loops; *: any nucleotide. **e** Cumulative plot showing the location of MEME (orange) and BEAM (blue) motifs in 100 nucleotide peak intervals. **f** CcpA UV cross-linking sites are mostly found in the first half of the BEAM structural motifs. Plotted are the cumulative deletion frequencies around the centre of the loop in each hairpin structure (blow the plots). Predicted CcpA cross-linking sites are indicated. **g** Venn diagram showing the number of peak sequences that have BEAM and MEME motifs.

Analysis of the distribution of the CcpA binding peaks on sRNAs and mRNAs revealed that the protein was consistently cross-linked around 10-30 nucleotides upstream of 3’ ends of non-coding sRNAs (Fig. 5a) and mRNAs (Fig. 5b). Although many tRNAs were enriched in the CcpA CRAC data (Fig. 4 and Supplementary Fig. 4), this 3’ end enrichment was not evident in this class of transcripts. Several examples of CcpA binding to RNA transcripts are shown in Supplementary Fig. 4. To test whether CcpA has any preference for binding sequence or structural elements, we first used pyCalculateFDRs^29^ to identify significantly enriched CcpA binding peaks in the data. We then normalised the peak intensities to mRNA expression levels using RNA-seq data and selected the top 200 peaks for further analyses.

We subsequently used BEAM^30^ and MEME^31^ to identify enriched structural and sequence motifs, respectively. MEME identified U-rich sequence motifs that were enriched in sRNAs, and mRNAs bound by CcpA (Fig. 5c, Supplementary Fig. 5a and MEME motif I, Supplementary Data 4). We also recovered a sequence motif found in cross-linked tRNAs (Supplementary Fig. 5a; MEME motif II, Supplementary Data 4). Interestingly, BEAM identified hairpin structures in peaks that were enriched in peaks located in sRNAs and mRNAs (Fig. 5d). Many of these structures consisted of helices interrupted by one or two internal bulges (Supplementary Data 4). These BEAM motif structures were generally located upstream of the U-rich motifs identified by MEME (Fig. 5e). To pinpoint the possible RNA-binding sites, we analysed the distribution of the deletions around the MEME and BEAM motifs as these are often a hallmark for UV cross-linking sites^14,32^. CcpA preferentially cross-linked 5-15 nucleotides upstream of the sequence motifs identified by MEME (Supplementary Fig. 5b, c) and ~15 nucleotides upstream of the hairpin loop in BEAM structural motifs (Fig. 5f). These data suggest that CcpA binds upstream of the sequence motifs identified by MEME. Around a quarter of the MEME I motifs found in sRNAs and mRNAs also contained a structural motif identified by BEAM in the same peak sequence (Fig. 5g).

The striking enrichment of CcpA near 3’ ends of sRNA and mRNA transcripts near hairpin structures and U-rich sequence elements led us to the hypothesis that CcpA may bind RNAs at or near intrinsic transcription terminators.

### The CcpA HTH domain is required for efficient binding to RNA in vivo and in vitro

**Fig. 6:**
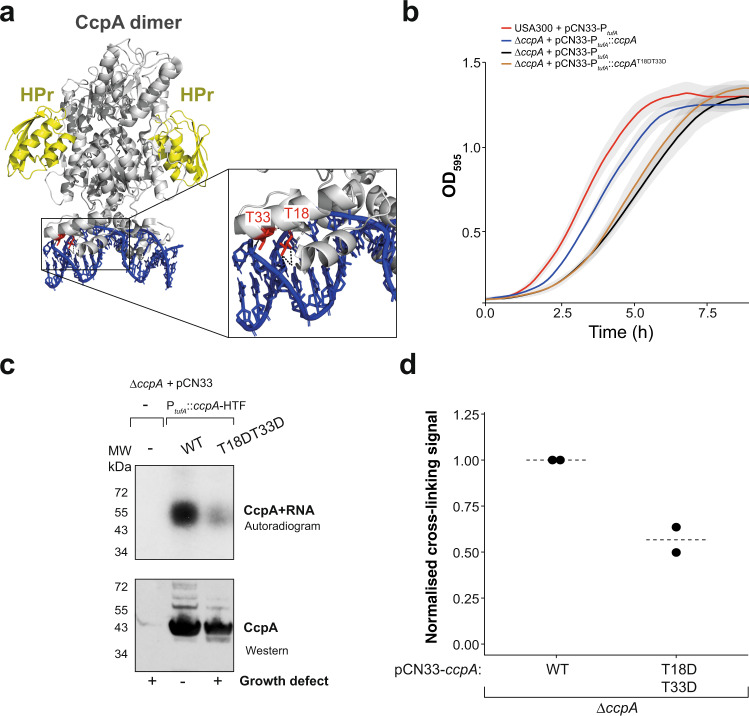
The CcpA HTH domain is required for efficient RNA-binding in vivo. **a** Crystal structure of a CcpA-HPr-dsDNA complex (grey, yellow and blue, respectively) containing a 16 bp CRE dsDNA substrate (adapted from^34^; PDB code 1RZR https://www.wwpdb.org/pdb?id=pdb_00001rzr). The zoomed-in region shows the location of two threonines that are required for dsDNA binding. HPr histidine-containing protein, CRE Catabolite Response Elements. **b** The CcpA T18DT33D mutation causes a growth defect. Growth curves were performed in a plate reader using the following strains: USA300 + pCN33-P_*tufA*_, USA300 Δ*ccpA*, USA300 Δ*ccpA* + pCN33-P_*tufA*_::*ccpA* and the USA300 Δ*ccpA* + pCN33-P_*tufA*_::*ccpA*^T18DT33D^. Data are presented as mean values (solid lines) +/− SD (shaded region). **c** T18 and T33 are required for efficient RNA-binding in vivo. Shown is a result from a CcpA CRAC experiment using the Δ*ccpA* as a negative control, the WT CcpA-HTF protein as positive control and the CcpA T18DT33D-HTF mutant. **d** Quantification of CcpA-HTF WT and T18DT33D cross-linking data. Autoradiography and Western blot signals from two independently performed CRAC experiments (shown in **c**) were quantified, and the autoradiography signal was normalised to the CcpA protein levels. Original data and pictures for **b**–**d** are provided in Source data.

We next asked whether CcpA uses its HTH domain to bind RNA in vivo. Two conserved threonine residues in the CcpA HTH domain play a key role in DNA binding (Fig. 6a; T33 and T18)^33^. Phosphorylation of these sites inhibits DNA binding in vitro^33^. To test whether these amino acids also play a role in RNA binding, we generated *ccpA*-HTF expression constructs containing wild-type (WT) *ccpA* or a phosphomimetic *ccpA* mutant (T18DT33D) and transduced these into the USA300 *ΔccpA* strain. Deleting *ccpA* resulted in a significant growth defect (*P* value = 0.02866; Supplementary Fig. 6), which could be partially restored by expressing WT *ccpA* from a plasmid (Fig. 6b and Supplementary Fig. 6). The phosphomimetic mutant, however, was unable to restore growth (Fig. 6b and Supplementary Fig. 6). Moreover, the T18DT33D mutations reduced in vivo UV cross-linking to RNA by ~50% (Fig. 6c, d; see Source data).

**Fig. 7:**
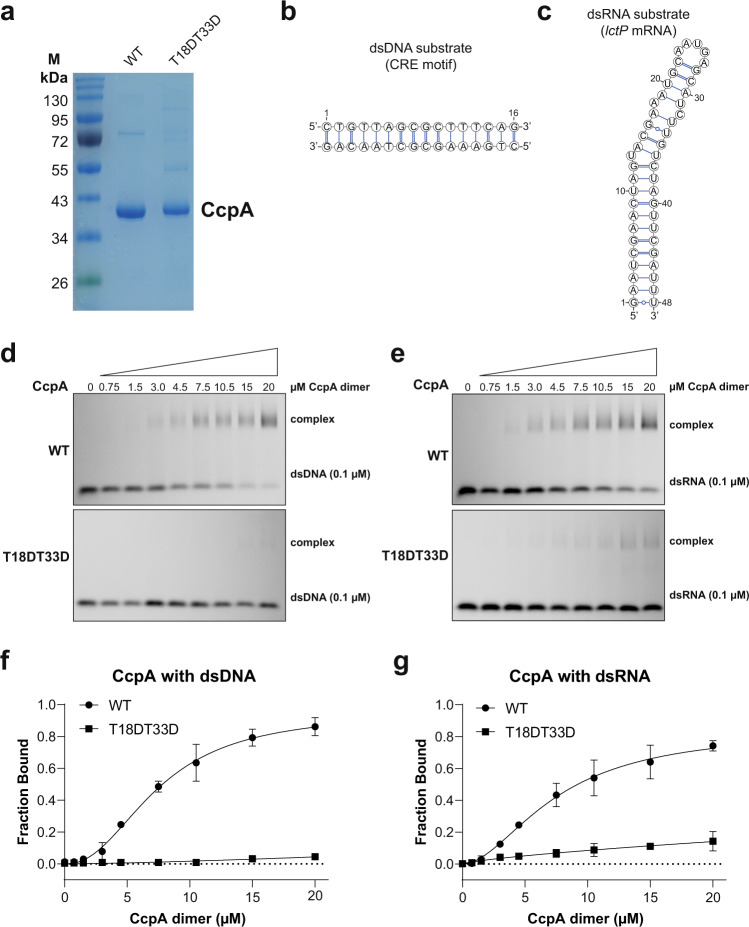
The CcpA HTH domain is required for efficient binding to RNA in vitro. **a** SDS-PAGE of recombinant CcpA WT and T18DT33D phosphomimetic mutant used for EMSA. The original picture of one representative gel was provided in the Source data file. **b**, **c** dsDNA and dsRNA substrates used for the EMSA. **d**, **e** EMSA performed with increasing amounts of recombinant CcpA proteins (0, 1.5, 3, 6, 9 15, 21, 30 and 40 µM) and IRD800-labelled dsDNA and dsRNA substrates (0.1 µM) as well as a 50-fold excess of a non-specific competitor (poly(dI-dC)). Complexes were subsequently resolved on 1% TBE-agarose gels. Two replicate experiments were performed. Source data are provided in the Source data file. **f**, **g** Quantification of the EMSA results. Band intensities were quantified by ImageQauntTL v8.2.0.0. The binding curve and Kd value were obtained from Graphpad 9.3.1, assuming CcpA binds DNA and RNA as dimer. Two independent replicate experiments were used for quantification. Data are plotted as mean ± SD. The binding affinity (Kd) of CcpA WT to dsDNA substrate is 7.97 ± 0.54 µM (95% confidence interval), while to dsRNA is 9.72 ± 0.92 (95% CI).

To complement these analyses, we also examined the binding of recombinant WT CcpA and the phosphomimetic mutant (Fig. 7a) to DNA and RNA substrates in vitro using Electro Mobility Shift Assays (EMSA). CcpA regulates gene expression by binding to (partially) palindromic DNA sequences referred to as Catabolite Response Elements (CRE). We used a 16 bp CRE dsDNA binding site previously used as a DNA substrate in CcpA co-crystallisation studies^34^ (Fig. 7b). The BEAM motif found in the *lctP* mRNA was used as a dsRNA substrate (Fig. 7c). CcpA bound to both double-stranded DNA and RNA in our EMSAs (Fig. 7d and e) with similar affinities (7.97 ± 0.54 and 9.72 ± 0.92 µM, respectively; Fig. 7f, g). Consistent with previous work^33^, the T18DT33D mutations almost completely abolished binding to the dsDNA substrate (Fig. 7d, f). These mutations also substantially reduced binding to the dsRNA substrate (Fig. 7e, g). Given that CcpA binds both nucleic acids with similar affinities, plus the fact that the T18DT33D mutations substantially reduced binding to both DNA and RNA substrates, implies that the binding of CcpA to RNA is biologically meaningful.

Collectively, our results show that the HTH DNA-binding domain is also required for efficient binding to RNA substrates in vivo and in vitro.

### CcpA may regulate the stability of its RNA substrates

**Fig. 8:**
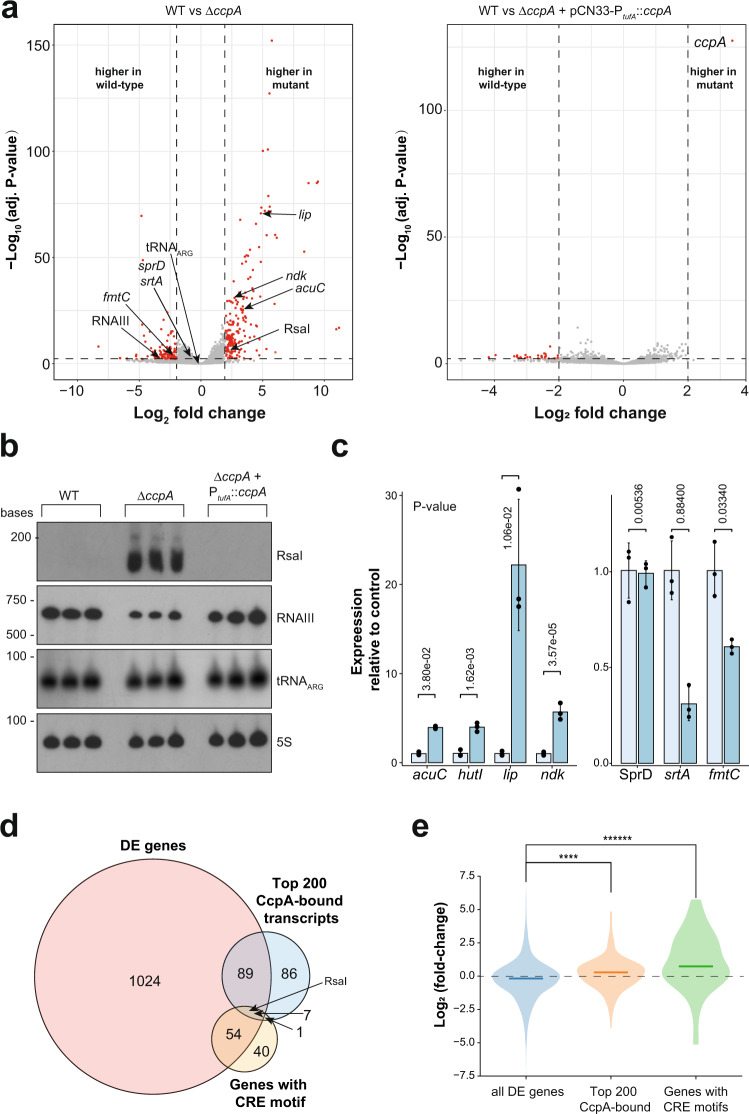
CcpA regulates gene expression at both the transcriptional and post-transcriptional level. **a** Deletion of *ccpA* causes global changes in gene expression. DESeq2 analyses were performed on RNA-seq data from three independent biological replicates (Supplementary Data 5). The left volcano plot shows the comparison between the USA300 WT strain and the USA300 Δ*ccpA* strain. The right volcano plot shows the comparison between the USA300 WT and the complemented strain. Red dots indicate genes that are significantly enriched in the WT (left side) or the mutant strains (right side). Transcripts with a log-fold change >2 and a −log_10_ *P* value of 1.3 or higher were considered enriched in cross-linked data. Benjamini and Hochberg adjusted *P*-values were calculated using DESeq2^28^ on data from three independent biological replicate RNA-seq samples. **b** Northern blot validation of selected transcripts highlighted in **a**. RNA from three independent replicate experiments was used. **c** Expression of selected transcripts highlighted in **a**. Shown are the mean and standard deviations of three independent experiments. Statistical significance was measured through two-sided Student’s unpaired *t* test (uncorrected *P* values marked above each comparison). Source data from **b**, **c** are provided in the Source data file. **d** Overlap between the genes differentially expressed (DE) in Δ*ccpA* with transcripts enriched in the CcpA CRAC data (top 200 CcpA-bound transcripts) and genes with a CRE-motif in promoter regions (genes with CRE motif). **e** Violin plot showing the distribution of fold-changes in mRNA target expression (*y* axis) in the Δ*ccpA* strain for all the differentially expressed genes (all DE genes), transcripts that are strongly bound by CcpA in the CRAC data (Top 200 CcpA-bound) and for all the genes with a CRE motif. The horizontal line in each violin plot indicates the median value. ﻿Statistical significance between the groups was calculated using a two-sided Mann–Whitney *U* test (no *P* value adjustment). ****P value = 9.96e−05, ******P value = 7.134e−12.

To better understand how CcpA contributes to post-transcriptional regulation of gene expression, we compared the steady-state RNA levels from WT USA300 cells to USA300 cells lacking CcpA (Δ*ccpA*). This revealed that over 1000 genes were significantly differentially expressed in the deletion mutant (Fig. 8a and Supplementary Data 5: WT vs Δ*ccpA*). These changes in gene expression could largely be restored by complementing the Δ*ccpA* with a plasmid containing the WT CcpA (Fig. 8a and Supplementary Data 5: WT vs Δ*ccpA* + pCN33-P_*tufA*_::*ccpA*/complemented strain). To validate the results of the differential expression analyses, we performed Northern blot and RT-qPCR analyses to quantify RNA steady-state levels of selected transcripts bound by CcpA (Fig. 8b, c). These data were in good agreement with the DESeq2 results (Fig. 8a).

To gain insight into which of the differentially regulated genes are controlled by CcpA at the transcriptional level, we searched for CRE motifs in the USA300 genome located in promoter regions (see Methods for details). This identified 102 genes with CRE sequences in promoter regions that were significantly enriched for genes differentially expressed in Δ*ccpA* (Fig. 8d; hypergeometric test; *P* value = 2.04e−06). Moreover, the majority of these genes were also upregulated in Δ*ccpA* (Fig. 8e; Genes with CRE motifs), consistent with the idea that CcpA predominantly acts as a transcriptional repressor^35,36^. Interestingly, most of the transcripts highly enriched in the CcpA CRAC data did not have any recognisable CRE motifs near the promoter regions of the corresponding genes (Fig. 8d). A notable exception is RsaI, a small RNA that is downregulated by CcpA in the presence of glucose^37^ and CcpA also binds to the transcription terminator of the RsaI. Those transcripts that were strongly bound were also highly enriched for differentially expressed RNAs (Fig. 8d; hypergeometric test; *P* value = 1.033e−05) and frequently upregulated in Δ*ccpA* (Fig. 8e; Top 200 CcpA bound). Thus, these data imply that CcpA can also repress the expression of many genes at the post-transcriptional level. It is of course possible that some of these genes could still be regulated by CcpA at the transcriptional level as they may contain promoter elements that show some similarity to the classical CRE motif but were missed in our bioinformatics analyses.

## Discussion

Here we present the first comprehensive analysis of RBPs in a Gram-positive bacterium, using clinically relevant *S. aureus* strains. We identified hundreds of RBPs in *S. aureus*. Many of these RBPs had not been described to bind RNA before and many are unique to this Gram-positive bacterium. Our results imply that many metabolic enzymes in this organism can bind RNA and suggest that HTH-domain DNA-binding proteins, as well as Rossman-fold containing proteins, may also function as post-transcriptional regulators. Subsequent validation studies revealed that most proteins selected for further studies cross-linked to RNA in vivo, strongly suggesting that they are indeed RBPs and demonstrating the robustness of our approach.

To explore the RNA-binding proteome in *S. aureus*, we used two independent orthogonal purification principles that employ UV irradiation to covalently link RBPs to their direct RNA substrates. The reason for applying two different approaches was because we had previously shown that the individual protocols to purify ribonucleoparticles (RNPs) do not necessarily yield identical results^38^. Much to our surprise, the 2C method recovered over 1,000 putative *S. aureus* RBPs, which is on par with a previous study that used silica beads to purify *E. coli* RBPs^11^. These data suggest that about a third of the *S. aureus* proteome is an RBP, which seems rather high considering other studies reported that 7.5-15% of eukaryotic and prokaryotic proteomes are RNA-binders^8,9,17,39^. We suspect that 2C and related methods may overestimate the number of RBPs and may produce more false positives. UV irradiation can also cross-link histones to DNA^26^ and although we extensively treated our samples with DNase during the purification of RBPs, it is still possible that we purified DNA cross-linked proteins to some extent. This emphasises the importance of validating the results using alternative approaches. As a control experiment, we performed CRAC experiments where we incubated cross-linked CcpA RNP complexes with increasing amounts of RNase or DNase. This revealed that the CcpA RNP is sensitive to RNase but not DNase (Fig. 3h). Thus, we conclude that our UV irradiation conditions favour cross-linking of proteins to RNA (Fig. 3h).

PTex on *S. aureus* recovered a lower number of enriched proteins (384; Fig. 1e); however, this number is comparable with the number of *E. coli* proteins identified using the OOPS organic extraction protocol (364 proteins)^17^. We note that application of the phenol-based technique to *S. aureus* resulted in higher background levels of non-cross-linked proteins (Fig. 1c), which may explain why fewer proteins were statistically significantly enriched in the mass spectrometry data. This could be due to technical reasons, perhaps because of spillover from separate phases during the extraction procedure. With both 2C and PTex we noticed that the amount of background is correlated with the amount of input material and therefore this should be carefully optimised. Regardless, even though both methods have completely different purification approaches, and have their own unique technical limitations, the overlap between the two data sets and other existing data sets was remarkably high (Fig. 1g, h). In addition, those RBP candidates that were recovered by both PTex and 2C were also all biochemically validated to bind to RNA (Fig. 3). Collectively, these data provide compelling evidence that both methods reliably recover RBPs. We would, however, strongly recommend applying both approaches at the same time when performing RBPome analyses for the following reasons: We identified many nucleotide-binding enzymes in our 2C data, including ATPases, GTPases and NAD(P)-binding proteins. One could argue that such a high recovery of these types of proteins is an artefact of the 2C technique as, depending on the buffer conditions used, the silica resin might also recover proteins cross-linked to short nucleotides. However, many of these nucleotide-binding proteins were also detected in the PTex-purified fraction and successful enrichment of RBPs by PTex requires that the cross-linked RNA is at least 30 nucleotides^6,13^.

Thus, these findings support the notion that the nucleotide-binding proteins we recovered are indeed RBPs.

One major advantage of the organic phase extraction protocols, such as PTex^13^ and OOPS^17^, is that these methods not only enable reliable identification of RBPs, but by sequencing the cross-linked RNAs one can also identify protein-binding sites within the RNA. The adducts formed by protein-RNA cross-linking can be a major obstacle for reverse transcriptases when preparing cDNA libraries and this often leads to the introduction of mutations in the cDNA sequences. Mutations, in particular deletions, have previously been shown to be a hallmark for UV cross-linking sites^14,40^ and using this information one can therefore pinpoint protein-binding sites on RNA. Such analyses will be more challenging with silica-based approaches as this also recovers the non-cross-linked RNA, which is present in vast excess in the purified material.

Taken together, our double-sided approach allowed us to comprehensively map the RBPome of a bacterium while at the same time critically assessing the obtained high-throughput results.

We previously found that the UV cross-linking efficiency increases when cells are irradiated in water or transparent media^15,41^. Therefore, we used two different media for our UV cross-linking studies. Indeed, we recovered a much larger number of proteins in the transparent Low Phosphate Medium (LPM). However, since LPM lacks phosphates and the preferred carbon source glucose, it is certainly possible that we detected more cross-linking because the cells were mounting a nutrient stress response and were remodelling their transcriptome to utilise the available glycerol and amino acids in this medium. Therefore, we cannot rule out the possibility that the increased cross-linking we observed is because some proteins, such as RNA nucleases, simply bind higher numbers of and/or more diverse RNA substrates under these stressful conditions. Indeed, it is conceivable that some of the RBPs we detected may only significantly bind RNA under nutrient stress conditions. While testing these hypotheses is beyond the scope of the current manuscript, we are performing 2C experiments, as well as RNA-seq and proteomics, under a variety of stressful growth conditions, such as pH shock and treatment with antibiotics. Our premise is that those proteins that show increased RNA binding under such stress conditions could be valuable targets for the development of novel antimicrobials.

Much to our surprise, many HTH-type transcription factors were recovered in our 2C. DNA-binding proteins with RNA-binding activity (including transcription factors), have previously been identified in eukaryotes and prokaryotes^42^ and are also frequently detected in RBPome analyses in eukaryotes^43^. The *S. aureus* HTH-type transcription factor SarA was previously shown to interact with RNA in vivo, however, it remained unclear if the HTH domain of SarA was responsible for this interaction^24^. One of the HTH proteins we characterised further is the CcpA transcription factor that plays key role in regulating carbon metabolism^44^. Strikingly, we demonstrate that CcpA binds hundreds of coding and non-coding RNA transcripts in vivo near intrinsic transcription terminators. This suggests that CcpA could have a role in transcription termination or regulates the stability of these RNAs. Consistent with the former, *Streptococcus oligofermentans* CcpA binds tRNA^Arg^ in vitro and deletion of *ccpA* resulted in modest transcriptional readthrough of tRNA^Arg^ ^45^. This points towards a role for CcpA in transcription termination. Additionally, *B. subtilis* CcpA associates with the NusA transcription factor^46^ that plays a role in the termination of some intrinsic terminators^47^. We did not observe major changes in termination efficiency or transcription readthrough of CcpA RNA substrates in the RNA-seq data of the *ccpA* deletion mutant. However, it is certainly possible that these extended species are low-abundant, generally short-lived and may only accumulate to detectable levels in strains lacking RNA decay factors. Our results do imply that CcpA binding near transcription terminators may (either directly or indirectly) impact the stability of the RNA. Those transcripts that are strongly bound to CcpA were also frequently upregulated in our *ccpA* deletion strain. Most of these CcpA-bound transcripts did not have any recognisable CRE motifs near the transcription start sites of the corresponding genes. It is therefore tempting to speculate that CcpA also regulates many genes solely at the post-transcriptional level. How binding of CcpA near intrinsic transcription terminators impairs the stability of the RNA is not yet clear.

Does CcpA have any RNA binding specificity? While CcpA clearly prefers binding CRE DNA motifs, our data do not provide compelling evidence that the protein recognises specific RNA sequences. The RNA structural motifs recovered by BEAM did not share any obvious sequence similarities. However, structurally, they were very similar, consisting of helices interrupted by short internal bulges (Supplementary Data 4). While U-rich sequences were frequently recovered in CcpA RNA-binding peaks, we found that the protein preferentially cross-links upstream of these motifs (Supplementary Fig. 5), suggesting that it does not directly bind to these U-rich elements.

To what extent does CcpA binding to RNA impact cell physiology? This question is difficult to address as CcpA utilises its HTH domain to bind both DNA and RNA. We would need to identify mutations that specifically disrupt RNA-binding, but not DNA binding, which is not trivial. These studies are currently underway.

## Methods

### Chromosomal HTF tagging of genes in *S. aureus*

Chromosomal tagging of genes with a codon-optimised HIS6-TEV-3xFLAG tag was performed using a two-step allelic exchange approach (integration and excision^48,49^). pIMAY vectors were constructed by Gibson assembly (Gibson Assembly® Cloning Kit, NEB) containing the HTF tag flanked by 500-1,000 bp sequences of the target gene (500–1000 bp upstream and downstream of the integrations site, respectively). Integration fragments were either generated by PCR or produced synthetically by Twist (Supplementary Data 6). Five µL of at least 1 µg/µL pIMAY vector was subsequently electroporated to the restriction defective *S. aureus* strain RN4220. Four µL of Dimethyl Sulfoxide (DMSO, Sigma) was added to each plasmid sample and after mixing with competent cells they were transferred to an ice-cold 0.1 cm cuvette (BioRad) and pulsed in a BioRad cell-pulser (cuvette size to 1, 2.1 kV/cm, 100 Ω and 25 µF). Immediately after electroporation, the cells were recovered in 1 mL of pre-warmed TSB. After shaking the cells for 2 h at 30 °C, they were plated on TSA in the presence of 10 µg/mL chloramphenicol (Cm10). Plates were incubated for 48 h at 30 °C. To confirm that the pIMAY plasmids were successfully transformed to RN4220 and were stably replicated, we used colony PCR: a colony was resuspended in 70 µL of lysis buffer (20 mM Tris pH 8, 3 mM MgCl_2_, 0.5% Tween 20, 0.5% NP-40, 60 µg/µL proteinase K) and then incubated at 55 °C for 1 h, followed by incubation at 95 °C for 10 min. The contents were then centrifuged at 6800 × *g* for 10 min, and then PCR was performed using 3 µL of the supernatant and Taq polymerase (NEB) with MSC primers (Supplementary Data 6). After confirming the successful transformation of the plasmids, they were transduced to USA300 or JKD6009 using a slightly modified phage transduction protocol^50^. To generate the phage, a single colony of *S. aureus* RN450 from a TSA plate was inoculated into 5 mL of TSB and grown at 37 °C overnight. The following day, the culture was diluted 1:50 in 25 mL TSB and grown until OD_600_ of roughly 0.3 at 37 °C. To induce phage production, mitomycin C was added to a final concentration of 2 µg/mL and the cells were shaken (80 rpm) at 32 °C for 3–4 h until lysis was completed. The phages were then filtered through a 0.4 µm filter and serially diluted in phage buffer (1 mM MgSO_4_, 4 mM CaCl_2_, 50 mM Tris pH 7.8, 100 mM NaCl, 0.1% gelatine) from 10^−3^ to 10^−8^ and stored at 4 °C until use. Selected RN4220 colonies were subsequently grown on a Brain heart infusion (BHI) agar slant (Oxoid) at 37 °C overnight. Cells were resuspended in 1 mL of TSB containing 5 mM sterile CaCl_2_. Ten µL of RN4220 bacterial cells and 10 µL of phage dilution (chose 10^−1^, 10^−3^ and 10^−6^) were mixed in 15 mL Falcon tubes. Subsequently, 3 mL of liquid phage top agar (equilibrated to temperature 45 °C for 1 h; 0.3% casamino acids, 0.3% yeast extract, 100 mM NaCl, 0.5% agar, freshly added 5 mM sterile CaCl_2_) was added to the mixture and poured onto 20 mL plates of phage bottom agar (0.3% casamino acids, 0.3% yeast extract, 100 mM NaCl, 1.5% agar, freshly added 5 mM sterile CaCl_2_). Plates were incubated overnight at 30 °C. Plates that showed near-confluent lysis were selected, 2 mL of phage buffer was added and incubated at 4 °C for 1 h. The top agar layer was scraped off and transferred with the buffer to a 15 mL Falcon tube. After centrifuging the tubes for 30 min at 2500 × *g* at 4 °C, the supernatant was passed through 0.45 µm filter. The flowthroughs containing the phages were stored at 4 °C. *S. aureus* cells from BHI slants were resuspended in 1 mL of TSB containing 5 mM CaCl_2_. Subsequently, 100 µL of cells were mixed with 10 µL or 100 µL of plasmid-containing phages. Phage buffer was added to the total volume of 300 µL plus 5 mM sterile CaCl_2_. The mixture was then incubated at 37 °C for 20 min with continuous shaking. Subsequently, 3 mL of liquid 0.3 GL top agar (0.3% casamino acids, 0.3% yeast extract, 100 mM NaCl, 0.15% sodium lactate, 0.1% glycerol, 1.5 mM trisodium citrate, 0.5% agar, pH 8) was added, mixed, and poured over 30 mL plates containing 0.3 GL bottom agar (0.3 GL top agar but with 1.5% agar, 25 µg/mL chloramphenicol (Cm25)) containing the appropriate antibiotic. The plates were then incubated overnight at 30 °C for 2 days. Colonies were screened by PCR as described above for RN4220 to identify cells that were successfully transduced. These were subsequently cultured in TSB Cm10 at 37 °C for at least 3 days with continuous shaking to force integration of the plasmid into the chromosome. Colony PCR was subsequently performed to identify cells that integrated the pIMAY constructs using three sets of primers (see Supplementary Data 6). To excise the pIMAY backbone vector from the chromosome, cells were grown in TSB and plated in several dilutions onto TSA containing 0.25 μg Anhydrotetracycline (ATc). Colony PCR was subsequently performed to identify cells that had correctly excised the plasmid (Supplementary Data 6). Western blotting was performed to confirm the HTF tag was correctly integrated. Each strain was also verified by Sanger sequencing the integration sites.

### Generation of *ccpA* (mutant) constructs

A *ccpA-*HTF fragment was PCR amplified from the USA300*::ccpA-*HTF strain with 24 and 90 bp flanking sequences that included BglII and KpnI restriction sites, ribosome binding sites and the transcriptional terminator. PCR products were ligated into pJET1.2/blunt cloning vector (CloneJET PCR Cloning Kit, Thermo Scientific™ K1231) and verified by Sanger sequencing. The DNA fragments were then cloned into the pCN33-P_tufA_ expression vector (P_tufA_ is the promoter). The T18DT33D mutant was generated using the Q5® Site-Directed mutagenesis kit (NEB E0554S).

### Cell culture and UV cross-linking

A list of all the strains used in this study is provided in Supplementary Data 6. *S. aureus* strain JKD6009 RNase III-HTF and USA300 RNase III-HTF were used in the RBPome capture. Both strains were inoculated in TSB (Tryptone soya broth, Oxioid CM0129) and cultured overnight at 37 °C, 200 rpm shaker. Strain JKD6009 RNase III-HTF was sub-cultured into 190 mL LPM^51^ (Low phosphate, low magnesium medium, 5 mM KCl, 7.5 mM (NH_4_)_2_SO_4_, 0.5 mM K_2_SO_4_, 8 µM MgCl_2_, 1 M KH_2_PO_4_, 16 mM Tris-HCl pH 7.8, 0.1% Casamino acids, 0.3% Glycerol) and grew from OD_600_ 0.01 to 1. Overnight cultures of USA300 RNase III-HTF were re-inoculated to 65 mL fresh TSB to OD_600_ = 0.05 and left to grow to an OD_600_ = 3. Cells were then filtered through 0.45 µm filters using a vacuum filtration device (UVO^3^) shifted to the same volume of LPM medium for 15 min at 37 °C and UV irradiated in LPM in the Vari-X-linker (*λ* = 254 nm) (https://www.vari-x-link.com^27,41^) with different UV intensities. For the 2C optimisation experiments (Fig. 1 and Supplementary Fig. 1), cells were irradiated with 1 and 2 J/cm^2^ of UV light (254 nm). Control and UV irradiated samples were collected by filtration and stored at −80 °C before use. In total, six replicates were collected (three technical and two biological) for each experiment.

For CRAC experiments, strains expressing HTF-tagged proteins were grown overnight in TSB at 37 °C. Cells were subsequently diluted to OD_600_ = 0.05 in 100 mL fresh TSB and UV irradiated at OD_600_ = 3. For the CRAC experiments shown in Fig. 3, cells were either untreated or irradiated with 250, 500, 1000 mJ/cm^2^ of UV at 254 nm in the Vari-X-linker. Cells were pelleted by centrifugation and stored at −80 °C.

### CRoss-linking and CDNA analysis (CRAC) in *S. aureus*

*S. aureus* cells expressing HTF-tagged proteins were grown to saturation in TSB at 37 °C. Cells were subsequently diluted in 100 mL fresh TSB to OD_600_ 0.05, grown to an OD_600_ of roughly 3 and 100 mL of cells was subsequently UV cross-linked (254 nm; 1000 mJ/cm^2^) in the Vari-X-linker (UVO3^27,41^). As negative controls, we either used UV irradiated cells from the parental strain (CcpA CRAC experiments; Fig. 4) or non-UV irradiated cells (Fig. 3). For the CRAC validation experiments shown in Fig. 3, cells were subjected to three different UV doses: 250, 500, 1000 mJ/cm^2^. To identify the RNAs that were cross-linked to CcpA (Fig. 4), cells were harvested by filtration and shifted to an equivalent volume of LPM medium for 15 min. Cells were collected by filtration and flash-frozen on the filters and stored at −80 °C. For the CRAC experiments, the cells were washed off the filters with 25 mL of ice-cold phosphate buffer saline (PBS) and pelleted by centrifugation. Cells (up to 0.5 g) were subsequently resuspended in 2 volumes/cell weight of TN150-Lysostaphin buffer (50 mM Tris pH 7.8, 150 mM NaCl, 100 µg/mL Lysostaphin, 0.1% NP-40, 0.5% Triton X-100) and transferred to 5 mL screw-cap tubes (Eppendorf). Sixty µL of RQ DNase 1 and 10 µL of SUPERase·In were added to the mixture and incubated at 37 °C for 10 min to lyse the cells. 3 V/cell weight of Zirconia beads (0.1 mm) were added, and the mixture was vortexed vigorously 5 times for 1 min with 1-min incubations on ice between each step. 2 V/cell weight of cold TN150 anti-peptidase (50 mM Tris pH 7.8, 150 mM NaCl, 1 Roche EDTA-free Protease Inhibitor Cocktail mini pellet, 0.1% NP-40, 0.5% Triton X-100 and 10 mM EDTA) buffer before centrifugation for 30 minutes at 12,100 × *g* at 4 °C. Subsequently, 75 µL Anti-FLAG^®^ M2 Magnetic Beads (pre-washed with 3 times 1 mL TN150 buffer; Sigma Aldrich, M8823-5ML) were added and incubated with the lysate for 2 h at 4 °C. The beads were then washed three times 5 min with 2 mL TN1000 buffer (50 mM Tris pH 7.8, 1 M NaCl, 0.1% NP-40, 0.5% Triton X-100) and three times with 2 mL TN150 buffer (50 mM Tris pH 7.8, 150 mM NaCl, 0.1% NP-40, 0.5% Triton X-100) for 5 min. Beads were subsequently resuspended in 250 µL of TN15, 10 µL of home-made GST-TEV protease was added and the samples were rotated for 1.5–2 h at room temperature. TN150 was added to a final volume of 600 and 550 µL of the TEV eluate was incubated with RNace-it™ (Agilent Technologies 1 µL of 1:100 dilution) for exactly 5 min at 37 °C. Subsequently, 500 µL of the mixture was added to a tube with 0.4 g Guanidium-HCl and vortexed vigorously to inactivate the RNases. NaCl and imidazole (pH 8.0) were added to a final concentration of 300 mM and 10 mM, respectively and the mixture was transferred to 50 µL of Ni-NTA agarose beads (QIAGEN) equilibrated with Wash buffer I (300 mM NaCl, 10 mM imidazole, 6 M GuHCl, 50 mM Tris-HCl pH 7.8, 0.1% NP40, 5 mM β-mercaptoethanol (βMe) and 0.5% Triton X-100) and incubated at 4 °C overnight on a rotator. The next day, the beads were transferred to Pierce snap-cap columns (Thermo Scientific) and washed twice with 500 µL Wash buffer I and three times with 500 µL 1× NP-PNK buffer (10 mM MgCl_2_, 50 mM Tris-HCl pH 7.8, 0.1% NP40, 5 mM βMe and 0.5% Triton X-100). Radioactive labelling of 5’ ends, linker ligation and cDNA library preparation was performed as described previously^27^. Adaptor sequences, RT, and PCR oligos used for preparing the libraries are provided in Supplementary Data 6.

For the CRAC validation experiments shown in Fig. 3, cross-linked protein-RNA complexes were eluted from the nickel beads after radioactively labelling the 5’ ends of the cross-linked RNAs. Cross-linked protein-RNA complexes were precipitated with Trichloroacetic acid (TCA) and Acetone as previously described^14^. Radiolabelled complexes were resolved by NuPAGE, transferred to nitrocellulose membranes, and subsequently detected by autoradiography. Western blotting was performed with TAP Tag (Invitrogen™ CAB1001) and Goat anti-Rabbit IgG antibodies (Invitrogen™ A16096) to detect the proteins.

### Cloning, expression and purification of recombinant CcpA proteins

The *ccpA* wild-type (WT) gene was amplified by PCR using *S. aureus* USA300 chromosomal DNA as a template with primers (see Supplementary Data 6) containing NheI and XhoI restriction sites, respectively. The *ccpA*^T18DT33D^ mutant gene was amplified by PCR using the pCN33-P_*tufA*_*::ccpA*^T18DT33D^-HTF plasmid as a template with the same primers as used for amplifying the WT gene. The PCR products were subsequently digested with NheI (Thermo Scientific, FD0973) and XhoI (Thermo Scientific, FD0694) restriction enzymes and ligated into pRSET vector (see Supplementary Data 6) to generate pRSET-*ccpA* and pRSET-*ccpA*^T18DT33D^, which were subsequently transformed into *E. coli* BL21(DE3) (Invitrogen, C600003). Bacterial cells were grown in Luria Broth medium at 37 °C. At OD_600_ = 4, the expression was induced with 0.5 mM IPTG for 16 h at 18 °C. Cells were collected at 2500 × *g* for 30 min at 4 °C, then resuspended in lysis buffer A (300 mM NaCl, 50 mM Tris-HCl, pH 7.8, 10 mM imidazole, 5 mM DTT) supplemented with 1 mg/mL lysozyme (Sigma-Aldrich, L7651-5G) and protease inhibitor (cOmplete™, EDTA-free Protease Inhibitor Cocktail, Roche, 05056489001). Cells were lysed by sonication on ice for 5 min (10s-on, 50s-off, 80% amplitude). The lysate was cleared at 20,000 × *g* for 30 min at 4 °C. The supernatant was passed through a membrane filter with 0.45 µm pore size (MF-Millipore, HATF04700) and incubated with Ni-NTA beads (QIAGEN, 30230) for 1 h at 4 °C. The beads were transferred into a column and washed with buffer B (500 mM NaCl, 50 mM Tris-HCl, pH 7.8, 20 mM imidazole), and His-tagged protein was eluted with buffer C (300 mM NaCl, 50 mM Tris-HCl, pH 7.8, 5 mM DTT, containing 50 mM, 100 mM, 200 mM, 300 mM, and 400 mM imidazole) into different fractions. The purity was assessed by SDS-PAGE gel followed by SimplyBlue™ SafeStain (Invitrogen, LC6060). The cleanest fractions dialysis against buffer D (150 mM KOAc, 20 mM HEPES, pH 7.8) for 16 h at 4 °C. The protein sample was concentrated using Pierce™ Protein Concentrator PES with 10 K MWCO (Thermo Scientific, 88517). The concentration of protein samples was measured by Bradford assay. Protein samples were stored at 4 °C, then directly used for the following electrophoretic mobility shift assay, and at −80 °C for long-term storage.

### Electrophoretic mobility shift assay (EMSA)

The 16 nt dsDNA probe (see Supplementary Data 6) was purchased from IDT as two single-stranded oligonucleotides, with the sense strand labelled with IRDye800. The RNA probe (see Supplementary Data 6) was purchased from IDT, then labelled with IRDye800 (LI-COR, 929-80020) using the 5’ end labelling Kit (Vector Laboratories, MB-9001). The concentration of labelled RNA was determined by the absorbance of the dye at 780 nm. The DNA and RNA probes were resuspended in buffer containing 150 mM KOAc, 20 mM HEPES, pH 7.5, and annealed by heating up at 94 °C for 2 min and cooling down to 4 °C in 20 min. For the EMSA, a mixture in total 10 µL contained 150 mM KOAc, 20 mM HEPES, pH 7.5, 1 mM Mg(OAc)_2_, 0.5 µg poly(dI-dC) (non-specific competitor, Thermo Scientific, 20148E), 0.1 µM probe, and increasing concentrations (0–40 µM) of purified recombinant CcpA WT or T18DT33D phosphomimetic mutant. After 1 h of incubation on ice, the mixtures were loaded on 1% TBE-agarose gel and run in 1× TBE at 100 V for 1 h at 4 °C. Images were acquired using the Imagequant 800 system (Amersham) using the fluorescent IRlong channel.

### *S. aureus* 2C protocol

Cells were washed off the filters using 30 mL of ice-cold PBS (phosphate-buffered saline, pH 7.5) and transferred to a new 50 mL Falcon. Cells were harvested by centrifugation and resuspended in 300 µL 2C-lysis buffer (50 mM Tris-HCl pH 7.8, 150 mM NaCl, 0.1 % NP-40, 5 mM MgCl_2_, 5 mM CaCl_2_) in the presence of 10 µL RNase inhibitor (SUPERaseIn™, Invitrogen, AM2694), 10 µL of Lysostaphin (10 mg/mL, Prospect Bio ENZ-269) to degrade the cell wall, 10 µL of RQ DNase 1 (Promega, M6101) to degrade extra-and intracellular DNA and 6 µL of 50X protease Inhibitors (cOmplete™, EDTA-free Protease Inhibitor Cocktail, Roche, 4693132001). After a 20-30 min incubation at 37 °C, cells were lysed in a TissueLyser (Qiagen, 85220) for 5 min with 300 µL of 0.1 mm Zirconia beads (Biospec Products, 11079110zx). Samples were centrifuged in a microfuge for 12,400 × *g* for 20 min at 4 °C and the protein concentration was subsequently measured using the Qubit 4 system (Life Technologies). Clarified lysates were subsequently transferred to new tubes and added EDTA to a final concentration 25 mM for subsequent silica purification. 100 µg protein from the lysate was kept as “input” for protein level control. The 2C RBPome capture was adapted from a previously described protocol^12^. After preparing the lysates, four volumes of GTC RNA lysis buffer (Zymo Research, 1060-1) and five volumes of ethanol were added to the lysates and RBPs were captured by passing the mixture through the RNA-binding column (Zymo- Spin V-E, Zymo Research, C1024) using vacuum. To further minimise the recovery of DNA-binding proteins, we first washed the column with 400 µL of RNA wash buffer (Zymo Research, 1060-3) followed by a DNase treatment on the column (5 µL RQ1 DNase, 8 µL 10X RQ1 DNase buffer and 67 µL H_2_O mixture) for 15 min at room temperature. After washing the column with 400 µL RNA prewash buffer (Zymo Research,1060-2) and 400 µL RNA wash buffer, the RNA and cross-linked RBPs were eluted twice with 100 µL DEPC-treated H_2_O (Invitrogen, 750023). RNA and protein concentrations were determined using the Qubit 4 system (Life Technologies) and the integrity of the RNA was verified using the Agilent 2100 Bioanalyzer. The eluted RNA was subsequently degraded by treating the eluates with 25 units of Benzonase nuclease (Sigma-Aldrich, E8263) for 30 min at 37 °C. Proteins were denatured with 10 mM Dithiothreitol (DTT) at 55 °C for 30 min then mixed with three volumes of UA buffer (8 M urea in 100 mM Tris-HCl, pH 8). Denatured proteins were cleaned with filter-aided sample preparation columns (FASP, Microcon-30kDa Centrifugal Filter Unit, Millipore, MRCF0R030)^52^. After passing the mixture through the FASP column, the proteins were washed with 200 µL UA buffer. Proteins were alkylated in the dark using 100 µL 50 mM iodoacetamide (IAA, Sigma-Aldrich, I6125) at room temperature for 20 min and then washed with two rounds with 100 µL UA, followed by two rounds of washes with 100 µL Ammonium bicarbonate (ABC; 50 mM Sigma-Aldrich, 09830). MS Grade Trypsin Protease (1 µg per sample, Thermo Scientific, 90057) in 39 µL ABC was applied to the membrane and incubated at 37 °C overnight. Spin down the peptides and repeat elution with 40 µL ABC. Peptide concentrations were measured using NanoDrop spectrophotometers (Thermo Scientific, ND-2000C). Peptides were subsequently acidified with Trifluoroacetic acid (TFA) at pH ≤ 3 and then desalted using C18-StageTips^53^. Briefly, two pieces of C18 filters (Empore, 2215) were placed on the tips and activated with 15 µL methanol, followed by an equilibration step with 50 µL 0.1% TFA. Samples were passed through the StageTips and washed with 50 µL 0.1% TFA on the tips and subsequently eluted with 40 µL 80% Acetonitrile (ACN), 0.1% TFA).

### *S. aureus* PTex protocol

*S. aureus* USA300 strains expressing RNase III-HTF were grown to OD_600_ = 3.0 in TSB as described above and then shifted to an equal volume of LPM for 15 min. Cells were subsequently cross-linked in the culture medium with 2 J/cm^2^ of UV at 254 nm. Aliquots containing the equivalent cells of OD_600_ = 3.0 were pelleted in 2 mL safety-capped tubes, by centrifuging at 8,600 *g* for 5 min at 4 °C. Supernatants were removed, pellets snap-frozen with liquid nitrogen, and stored until use at −80 °C. As a negative control, non-UV treated cells were used. Three biological replicates of the Phenol-Toluol extraction (PTex) experiment were performed. To adapt the PTex protocol to Gram-positive pellets from 1 ml of untreated or UV cross-linked (2 J/cm^2^) *S. aureus* cells were resuspended in 200 µL of lysis buffer (50 mM Tris, 1 mM EDTA, 0.1% Triton X-100, 1 µg/µL Lysostaphin (Sigma), 0.05 U/µL RNase-free DNase I (NEB), 0.6 U/µL SUPERase-In (Invitrogen), pH 7.8). Non-UV cross-linked samples were partially RNA digested with RNase T1 (50 U/µL) and Benzonase (12.5 U/µL). Cells were lysed for 1 h at 37 °C with constant agitation at 1200 rpm. 10 µL of each sample (5%) was analysed by MS/MS for total proteome analyses.

After lysis, 400 μL of Tris-EDTA buffer (50 mM Tris, 1 mM EDTA, pH 7.8) was added and samples were subjected to the first two steps of the PTex protocol, as described before: lysates in 2 mL safe-lock tubes and were mixed with 200 μL neutral phenol (Roti-Phenol, Roth, 0038.3), 200 μL Toluol (Th.Geyer, 752.1000) and 200 μL 1,3-bromochloropropane (BCP) (Merck, 8.01627.0250) and vigorously agitated at 2,000 rpm (Eppendorf ThermoMixer) for 1 min at 21 °C. The phases were subsequently separated by centrifugation at 20,000 *g* for 3 min at 4 °C and the upper aqueous phase was carefully transferred to 2 mL tubes containing 300 μL of solution D (5.85 M guanidine isothiocyanate (Roth, 0017.3); 31.1 mM sodium citrate (Roth, 3580.3); 25.6 mM *N*-lauryosyl-sarcosine (PanReac AppliChem, A7402.0100); 1% 2-mercaptoethanol (Sigma)). Neutral phenol (600 μL), and BCP (200 μL) were added to each tube, mixed by vigorous centrifugation and phases were again separated by centrifugation as described above. Upper 3/4 of the aqueous phase and lower 3/4 of the organic phase was subsequently removed using a syringe and the interphase was then carefully transferred to 5 mL screw-cap tubes. Proteins were subsequently precipitated by mixing the samples with 9 volumes of 96% ethanol (analytical grade) and stored at −20 °C. Pellets were resuspended in 100 µL 2 M Urea containing 100 mM Tris-HCl, pH 8. RNA degradation, protein denaturation and protease digestion steps were performed as described for the 2C procedure above.

### Mass spectrometry analysis of RBPs

The tryptic peptides eluted from StageTips (80% ACN, 0.1% TFA) were lyophilised and resuspended in 0.1% TFA. Samples were analysed on a Fusion Lumos mass spectrometer connected to an Ultimate Ultra3000 chromatography system (Thermo Scientific, Germany) incorporating an autosampler. 5 μL of each tryptic peptide sample was loaded on an Aurora column (IonOptiks, Australia, 250 mm length), and separated by an increasing ACN gradient, using a 90 min reverse-phase gradient (from 3%–40% ACN) at a flow rate of 400 nL/min. The mass spectrometer was operated in positive ion mode with a capillary temperature of 275 °C, with a potential of 1,300 V applied to the column. Data were acquired with the mass spectrometer operating in automatic data-dependent switching mode, using the following settings: QExactive MS 70k resolution in the Orbitrap, MS/MS 17k resolution obtained by HCD fragmentation (26 normalised collision energy), top 12; Lumos, MS 120k resolution in the Orbitrap, MS/MS obtained by HCD fragmentation (28 or 30 normalised collision energy), read out either in the ion-trap with “rapid” resolution or in the orbitrap with a resolution of 30k with a cycle time of 2 s. To analyse the data from the RBPome MS/MS experiments, MaxQuant version 1.6.2.10/ 1.6.10.43^54^ was used for mass spectra analysis and peptide identification via Andromeda search engine^55^. Match between runs and iBAQ were chosen. Trypsin was chosen as a protease with minimum peptide length 7 and maximum of two missed cleavage sites. Carbamidomethyl of cysteine was set as a fixed modification and methionine oxidation and protein N-terminal acetylation as variable modifications. Proteome databases were obtained from GenBank nucleotide sequence database^56^: *Staphylococcus aureus subsp. aureus* USA300_FPR3757 (NC_007793.1) *and Staphylococcus aureus subsp. aureus str*. JKD6008 (NC_017341.1). First search peptide tolerance was 20 ppm and the main search peptide tolerance was set at 4.5. Peptide spectrum match (PSM) was filtered to 1% FDR.

### Data normalisation and quantification for mass spectrometry

Analysis of the MS/MS data was performed using the R programming language. For the data analyses, the iBAQ intensities from MaxQuant proteinGroups.txt output files were used. Contaminants, such as Benzonase and Trypsin, proteins which were only identified by post-translational modifications (PTMs) and reverse proteins from the decoy database were removed from the list. iBAQ intensities were log_2_ transformed. Protein numbers were counted for each experiment before imputing missing data. Pearson correlation coefficients for biological replicates were calculated using the R PerformanceAnalytics chart.Correlation package^57^. Correlation coefficients for all experiments were calculated with R cor function and Pearson method. Heatmaps were generated using the R superheat package^58^. For the total lysate “input” data, log_2_ transformed intensities were median normalised using normalizeBetweenArrays function from the limma package^59^ with the scale method. For the 2C RBPome data analyses, all intensities were normalised to the Trypsin signal, which was used as an internal standard. Trypsin scaling factors were calculated by dividing Trypsin intensities in each experiment by the maximum Trypsin intensity found in all samples and iBAQ intensities from each sample were subsequently divided by this scaling factor. Subsequently, the normalised intensities were log_2_ transformed and missing values were imputed by replacing missing values with data from downshifted normal distribution. Dendrogram clusters were generated by superheat package. Statistically significant differences between input cross-linked (CL) and non-cross-linked (nCL) samples were performed using the limma package^60^. *P* values were gene rated by empirical Bayes moderated *t* test in R package limma and adjusted using the Benjamini–Hochberg approach.

### Silver staining of 2C samples

Two hundred µL of RBPs captures by 2C were precipitated by mixing the sample with 1 mL 96% ethanol in the presence of 40 µg of glycogen. After > = 2 h incubation at −20 °C, the proteins were pelleted by centrifugation for 30 min at 16,900 *g* (Eppendorf centrifuge) at 4 °C. Pellets were washed with 1 mL 70% ethanol, briefly air-dried and resuspended in 20 μL of H_2_O. Samples were treated with 20 units of Benzonase for 30 min at 37 °C and divided over two NuPAGE™ 4-12% Bis-Tris Protein Gels (1.0 mm, Invitrogen NP0321BOX). The gel was subsequently silver stained (Pierce™ Silver Stain Kit (Thermo Scientific, 24612)).

### RNA extraction

Extraction of RNA from *S. aureus* was performed as previously described^61^. Briefly, cells were cultured to the desired optical density and rapidly harvested by centrifugation at 8600 × *g* for 5 min, 4 °C. Cell pellets were incubated with 100 µL of guanidium thiocyanate (GTC)-Phenol mix (pH 5.4; 1:1 ratio) and lysed by vortexing the cells for 5 minutes with 100 µL of Zirconia beads ﻿(0.1 mm; Biospec products 11079101z). ﻿Subsequently, 550 µL of GTC was added and the mixture was vortexed for several minutes. After a 10-min incubation at 65 °C for 10 min, the mixture was cooled on ice for 10 min. Phases were separated by adding 300 µL chloroform isoamyl alcohol (24:1) and by adding 1/10th of a volume of 3 M NaAc (pH 5.2) followed by vigorous vortexing. After 5 min of centrifugation in an Eppendorf centrifuge (12,400 × *g*), RNA was purified from the upper phase via two additional rounds of phenol-chloroform extraction and precipitated with 3 volumes of 96% cold ethanol. Pellets were washed with 1 mL of 70% cold ethanol, air-dried and resuspended in DEPC-treated water.

### RNA-seq analysis of USA300 and USA300 Δ*ccpA* strains

For the RNA-seq analyses described in Fig. 8, RNA was extracted from cells grown to OD_600_ of ~3 (late exponential phase). RNA from three biological replicates was extracted as described above and sequenced on an Illumina NovaSeq 6000 machine using the TruSeq library preparation protocol (Novogene).

### Western blot analysis

To detect the HTF proteins, nitrocellulose membranes were blocked for 1 h in PBST (PBS with 0.1% Tween-20 and 5% skimmed milk powder) and incubated with the monoclonal anti-FLAG® M2-Peroxidase (HRP) antibody (1:5000; Sigma-Aldrich A8592) or the anti-TAP antibody (1:5,000 dilution; ThermoFisher CAB1001) overnight at 4 °C. After the anti-TAP incubation, membranes were washed twice for 10 min with PBST and incubated overnight with Goat anti-Rabbit IgG antibodies (1:5000; Invitrogen™ A16096). The membrane was washed three times with PBST before chemiluminescence. Membranes were subsequently incubated with the Pierce™ ECL Western Blotting Substrate (Thermo Scientific; 32106) and proteins were visualised using films or the ImageQuant 800 system (Amersham).

### Northern blotting

*S. aureus* strains were grown overnight in TSB with erythromycin at 37 °C, diluted into fresh TSB supplemented with erythromycin the day after and grown to an OD_600_ value of 3. Total RNA was extracted through acid guanidinium thiocyanate-phenol-chloroform extraction as described above. Total RNA was resolved on a 6% polyacrylamide TBE-urea gel, transferred to nitrocellulose membranes via electroblotting and then UV irradiated (1200 mJ of 254 nm) to cross-link the RNAs to the membrane. Subsequently, membranes were pre-hybridised in 10 mL of UltraHyb buffer (Ambion) for 1 h at 37 °C. Membranes were then probed with a ^32^P-labelled DNA oligonucleotides (Supplementary Data 6) at 37 °C for up to 20 h. Membranes were subsequently washed twice with 100 mL of 2× SSC (pH 7.0, containing 0.3 M sodium chloride and 0.03 M sodium citrate), 0.5% SDS for 5 min at 37 °C. RNAs were subsequently detected by autoradiography.

### Reverse-transcription quantitative PCR (RT-qPCR)

The RT-qPCR analyses were performed on RNA samples extracted from corresponding strains that were grown to an OD_600_ of ~3.0. Total RNA was extracted as described above and treated with DNase I (RQ1 DNase; Promega) for 45 min at 37 °C in the presence of 2 U of SUPERasin. RNA was then purified using RNAClean XP beads (Beckmann Coulter). The RT-qPCRs were then performed using the Luna Universal One-Step RT-qPCR kit (NEB) according to the manufacturer’s instructions using 10 ng of total RNA. The PCR was run on a LightCycler 480 (Roche). Analysis of the qPCR data was performed using the IDEAS2.0 software. Ct values were calculated using the absolute quantification/fit points method with default parameters, and the fidelity of the PCR was examined through melt curve genotyping analyses. To calculate the relative fold-change of genes, the 2^(ΔΔCt) method was employed using 5S rRNA as a control. Each qPCR experiment was performed in biological and technical triplicates. For final data analyses, the mean and standard error of the mean of three biological triplicates was calculated and plotted. All oligonucleotides used for qPCR analyses are listed in Supplementary Data 6.

### Growth curves

The strains were grown at 37 °C overnight in 5 mL TSB supplemented with erythromycin (30 µg/mL) in a shaking incubator at 200 rpm. On the next day, the overnight cultures were diluted into fresh 200 µL TSB supplemented with antibiotic to reach OD_600_ = 0.05 and transferred to a Greiner 96 well flat bottom transparent polystyrene microplate. The plate was incubated at 36.5–37.5 °C in a Tecan infinite 200Pro pate reader with shaking (shaking duration 535 s and amplitude 6 mm) overnight. The optical density (OD_595_) was measured every 9 min. Three biological replicate experiments were performed each with three technical replicates.

### Computational analyses

#### Gene Ontology (GO) analysis and RBP predictions

To obtain UniProt IDs that can be recognised by PANTHER.d (http://pantherdb.org) and STRINGdb, JKD6008 and USA300 proteomes were blasted against *S. aureus* strain NCTC 8325:

makeblastdb -in UP000008816_93061.fasta -dbtype prot -title UP000008816_blastdb -out UP000008816_blastdb

blastp -query USA300.fasta -db UP000008816_93061_blastdb -out blast.txt -outfmt 6 -max_target_seqs 1.

Only proteins that had at least 80% similarity to the NCTC8325 strain were considered for further analyses.

The STRINGdb (Search Tool for the Retrieval of Interacting proteins database) R interface was used to test whether the 2C data were enriched for features such as Molecular Function (MF), Biological Process (BP), Cellular Component (CC), PFAM and InterPro classes (species searching ID 93061). *P* values and FDRs were generated using STRINGdb get_enrichment () function.

#### In silico prediction of RBPs using TriPepSVM and RBPPred

TriPepSVM analysis was performed as previously described^15^. For the prediction of *Staphylococcus aureus* RBPs, we used the following parameters: first, protein sequences from *S. aureus* strain USA300 were taken from uniprot.org (USA300_FPR3757.fasta). TriPepSVM accepts the following options:

./oligoPepSVM.sh [predictionFile] [OUTDIR] [TAXON_ID] [P] [COST] [RECURSIVE] [TRAINSET]

with the predictionFile being the protein sequences, the TAXON_ID representing the uniprot taxon identifier (1280), P the length of protein motifs (k-mer length = 3), COST being a support vector machine soft margin parameter, and RECURSIVE mode is used to train the programme with additional closely related bacteria from taxon 1280. Finally, TRAINSET is set to use random subsamples for training:

./oligoPepSVM.sh staph_usa300.fasta usa300_1280_T/ 1280 3 1 TRUE random

A total of 367 proteins were identified as putative RBPs using a score cut-off of 0.68. RBPPred analyses were performed on the USA300_FPR3757.fasta as previously described^19^.

#### RNA-seq bioinformatics analyses

Three experimental replicate data sets from the USA300 parental strain and USA300 Δ*ccpA* were processed using Flexbar^62^ to remove poor quality nucleotides (Phred score <23) and adaptor sequences. The reads are then mapped to the USA300 genome using Novoalign (version 2.07). Reads that mapped to multiple features in the genome were randomly distributed over the features. To determine to which genes the reads mapped to, we generated a modified USA300 annotation file in the Gene Transfer Format (GTF). This file contains the start and end positions of each gene on the chromosome as well as what genomic features (i.e., sRNA, protein-coding, tRNA) it belongs to. To generate this file, we used the Rockhopper software^63^ on all our USA300 rRNA-depleted total RNA-seq data and a minimal GTF file obtained from ENSEMBL (without UTR information). The resulting GTF file contained information not only on the coding sequences but also more complete 5’ and 3’ UTR coordinates. We then used pyReadCounters from the pyCRAC package^29^ (version 1.5.1) to count the total number of reads that map to each gene and genomic features. These tables with raw counts were subsequently used to perform a differential expression analysis using DESeq2^28^.

#### Identification of CRE motifs and putative CcpA regulated genes in the USA300 genome

We used the highly degenerate *B. subtilis* CRE motif consensus sequence (WTGNNARCGNWWWCAW^64^) to search for possible CcpA DNA-binding sites in the USA300 genome. Fuzznuc from the EMBOSS suite^65^ was used to extract all the motif locations in the USA300 genome on both strands. We allowed two mismatches in the motif. Custom Python scripts were used to convert the Fuzznuc output to the Gene Transfer Format (GTF). We then used bedtools^66^ to identify CRE motifs that were located within −80 to +50 of the 5’ end of transcripts. This identified 102 genes that may be regulated by CcpA at the transcriptional level.

#### CcpA CRAC bioinformatics analyses

##### Raw data analyses

Two biological CcpA CRAC experiments were performed, each with three technical replicates as previously described^14,27^. The cDNA libraries were sequenced on Illumina NovaSeq 6000 machines (Novogene) and Illumina MiniSeq systems. Raw sequencing reads in fastq files were processed using the paired-end CRAC data analysis pipeline developed by Sander Granneman, which uses tools from the pyCRAC package (version 1.5.1; https://git.ecdf.ed.ac.uk/sgrannem)^29^. The CRAC_pipeline_PE.py script first demultiplexes the data using pyBarcodeFilter.py and the in-read barcode sequences found in the L5 5’ adaptors. Flexbar^62^ then trims the reads to remove 3’-adaptor sequences and poor-quality nucleotides (Phred score <23). Using the random nucleotide information present in the L5 5’ adaptor sequences, the reads are then collapsed to remove potential PCR duplicates using pyFastqDuplicateRemover.py. The reads were then mapped to the USA300 genomes using Novoalign (www.novocraft.com; version 2.07). We then used pyReadCounters.py with Novoalign novo or .sam output files as input and the GTF annotation file to count the total number of unique cDNAs that mapped to each gene. Reads that mapped to multiple features in the genome were randomly distributed over the features.

##### Normalisation steps

Because the correlation between technical CcpA CRAC replicates was very high (Supplementary Fig. 3b), we merged them into one large data set for further analyses. This resulted in two large data sets for biological replicate experiments. Because the negative control samples produced very few reads (Supplementary Fig. 3a), we did not include them in the remainder of the analyses. To normalise the read count data for the CcpA-HTF samples generated by pyReadCounters.py and to correct for differences in library depth between these samples, we calculated Transcripts Per Million reads (TPM) for each gene in the two biological replicates. Briefly, raw counts for each gene were first divided by the gene length and then divided by the sum of all the values for the genes in that time point to normalize for differences in library depth. The TPM values for biological replicate were then log_2_-normalised.

##### Peak and motif distribution plots

PyCalculateFDRs.py was used to identify the significantly enriched CcpA-binding peaks (minimum 5 reads, minimum 20 nucleotide intervals, FDR ≤ 0.01). Next, pyBinCollector was used to plot the distribution of CcpA binding peaks around 3’ ends annotated in the GTF file of mRNA and sRNA transcripts (important pyBinCollector flags: -n 101 -r 50 -s exon --normalise). To generate the distribution profile for all sRNA and mRNAs individually (Fig. 5a, b), we normalised the total number of read peaks (assemblies of overlapping cDNA sequences) covering each nucleotide position by the total number of reads that cover the gene. Subsequently, using RNA-seq data generated under the same conditions as the CRAC data, we normalised the peak intensities (total number of reads in a peak) by the RNA-seq trimmed mean of *M* values (TMM^67^) for the gene that peak was located. The top 200 peaks with the highest normalised values were subsequently used for downstream analyses.

For the detection of RNA structural and sequence motifs, BEAM^30^ (version 2.5) and MEME^31^ were used using coordinates from the top 200 CcpA peaks that were extended to 100 nucleotides. We allowed any number of occurrences of motifs in the sequences and set the maximum motif length to 15.

### Reporting summary

Further information on research design is available in the Nature Research Reporting Summary linked to this article.

## Supplementary information

Supplementary Information

Reporting Summary

Description of Additional Supplementary Files

Supplementary Data 1

Supplementary Data 2

Supplementary Data 3

Supplementary Data 4

Supplementary Data 5

Supplementary Data 6

## Data Availability

The next-generation sequencing data have been deposited on the NCBI Gene Expression Omnibus (GEO) under accession codes GSE163719, GSE166151 and GSE189977. The mass spectrometry proteomics data have been deposited to the ProteomeXchange Consortium via the PRIDE^68^ partner repository with the data set identifiers: 2C RBPs (JKD6009, PXD023368; USA300, PXD023427), 2C total lysate (PXD023408) and PTex RBPs and total lysate (PXD023414). Figure 6a was generated using pymol and the PDB code 1RZR^34^ (https://www.rcsb.org/structure/1rzr). Source data are provided with this paper.

## Source data

Source Data

## Code availability

The Python pyCRAC (version 1.5.1) and GenBank parser packages are available from https://git.ecdf.ed.ac.uk/sgrannem/. The pyCRAC package is also available from pypi (https://pypi.org/user/g_ronimo/).
