# Supplementary material for: The RNA-bound proteome of MRSA reveals post-transcriptional roles for helix-turn-helix DNA-binding and Rossmann-fold proteins
Source: Nat Commun. 2022 May 24;13:2883. doi: 10.1038/s41467-022-30553-8 (PMC9130240; doi:10.1038/s41467-022-30553-8)
Supplement: Supplementary file 1 — Supplementary Information [file 41467_2022_30553_MOESM1_ESM.pdf]

Supplementary Information

The RNA-bound proteome of MRSA reveals post-transcriptional roles for helix-turn-helix DNA-binding and Rossmann-fold proteins

Liang-Cui Chu[1], Pedro Arede[1], Wei Li[1], Erika C. Urdaneta[2], Ivayla Ivanova[1], Stuart W. McKellar[1], Jimi Carlo Wills [3], Theresa Fröhlich[1], Alexander von Kriegsheim[3], Benedikt M. Beckmann[2] and Sander Granneman[1]*

Affiliations:

[1]Centre for Synthetic and Systems Biology, University of Edinburgh, Edinburgh EH9 3BF, UK.

[2]IRI Life Sciences, Humboldt University, 10115 Berlin, Germany.

[3]Cancer Research UK Edinburgh Centre, Institute of Genetics and Molecular Medicine, University of Edinburgh, Edinburgh, EH4 2XR, United Kingdom.

*To whom correspondence should be addressed:
Sander Granneman
e-mail: Sander.Granneman@ed.ac.uk
Tel: +44 131 6519082

Content:

Supplementary Figures: 2-10
Supplementary References: 11

Supplementary Figures and Figure legends

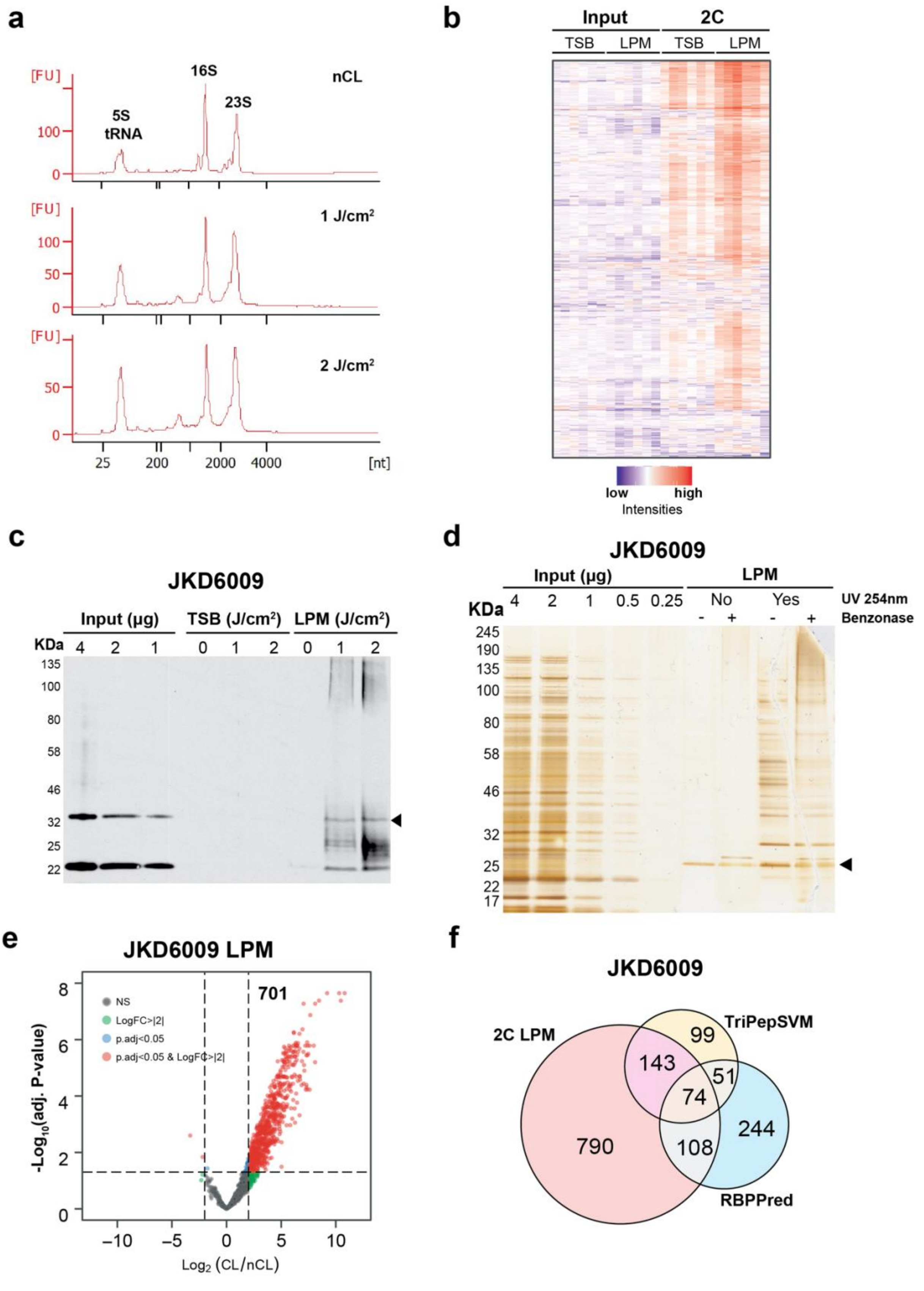
a
[FU]
5S
tRNA
16S
23S
nCL
1 J/cm²
2 J/cm²
25 200 2000 4000
[nt]
b
Input
2C
TSB LPM TSB LPM
low high
Intensities
c
JKD6009
Input (µg)
TSB (J/cm²)
LPM (J/cm²)
KDa
d
JKD6009
Input (µg)
LPM
No Yes
UV 254nm
Benzonase
KDa
e
JKD6009 LPM
NS
LogFC>|2|
p.adj<0.05
p.adj<0.05 & LogFC>|2|
701
-Log10(adj. P-value)
Log2 (CL/nCL)
f
JKD6009
2C LPM
TriPepSVM
RBPPred
790
143
99
74
51
108
244

Supplementary Fig. 1. Optimization of the 2C RBPome protocol in different *S. aureus* strains using two different growth media.

(a) Integrity of total RNA after irradiation with different doses of 254 nm UV light (0, 1 J/cm^2 and 2 J/cm^2). After UV treatment, total RNA was extracted, and a fraction was analysed by an Agilent Bioanalyser. The positions of tRNAs and rRNAs (5S, 16S and 23S) in the Bioanalyser results are indicated.

(b) UV cross-linking is generally more efficient in the colourless LPM medium. The heat map shows the normalised intensities (cross-linked normalised to control samples) of proteins detected in TSB and LPM input and 2C samples. The darker the red colour, the higher the intensities of the protein in the UV cross-linked samples compared to the untreated samples. The white colour indicates proteins that show no change in UV irradiated versus untreated samples.

(c) UV cross-linking is more efficient in the colourless LPM medium. Western blot analysis of 2C and PTex results on the JKD6009 strain expressing an HTF-tagged RNase III. 2C was performed with increasing UV cross-linking intensities (254 nm: J/cm^2). Following 2C, the recovery of RNase III-HTF (black triangle) was analysed by Western blotting using anti-FLAG antibodies.

(d) NuPAGE gel silver staining results of 2C captured RBPs in JKD6009 cells grown in LPM medium. Non-cross-linked cells were used as negative controls. The black triangle indicates the Benzonase enzyme that was used to degrade the RNA. (c)-(d) original pictures this experiment are provided in a Source Data file.

(e) Volcano plot of JKD6009 proteins enriched by 2C from cells grown in LPM medium. P-values were calculated using a moderate t-test on $\log_2$ transformed protein intensities from UV cross-linked (CL) and control (nCL) samples using the limma package. For the 2C results, only proteins with a $\log_2$-fold change >2 and a $-\log_{10}$ of the adjusted P-value of 1.3 or higher (indicated with dashed lines) were further considered (highlighted in red in the volcano plot). The number in the top right corner of the plots shows the total number of proteins that were enriched in the UV cross-linked samples. P-values were generated by empirical Bayes moderated t-test in limma and adjusted by Benjamini-Hochberg method.

(f) Overlap of RBPs in JKD6009 identified by 2C with RBPs predicted *in silico* using the TriPepSVM and RBPPred algorithms (see main text for details).

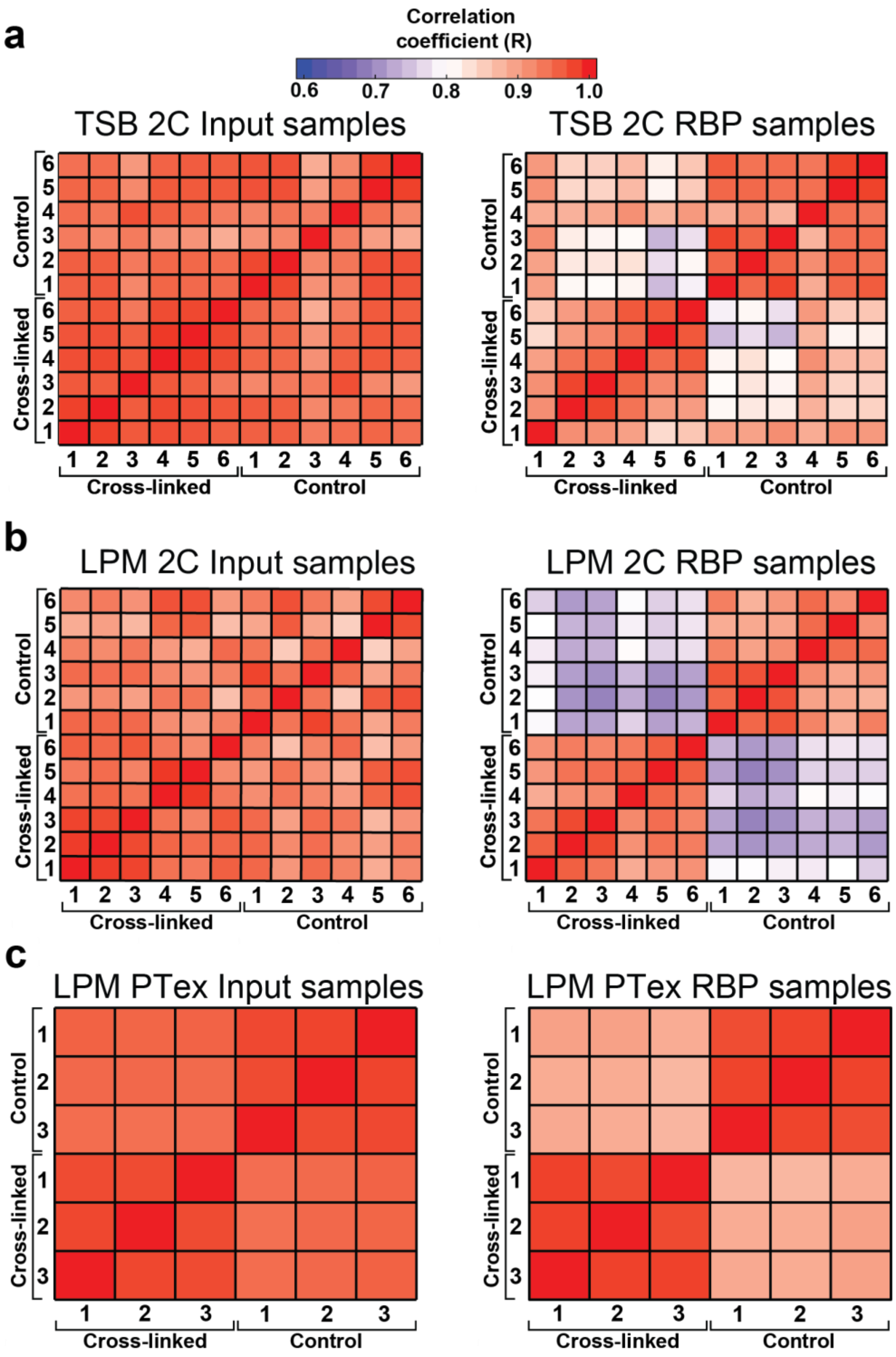

Supplementary Fig. 2. 2C and PTex RBPome data are highly reproducible.

(a-b) Pearson correlation coefficients of the protein signal intensities between the replicates (indicated by numbers below the heat maps) of total lysates (input) and 2C samples (TSB and LPM). These six samples consist of two biological and three technical replicates. 'Control' indicates the non-cross-linked samples.

(c) Pearson correlation coefficients of PTex replicate experiments (n=3).

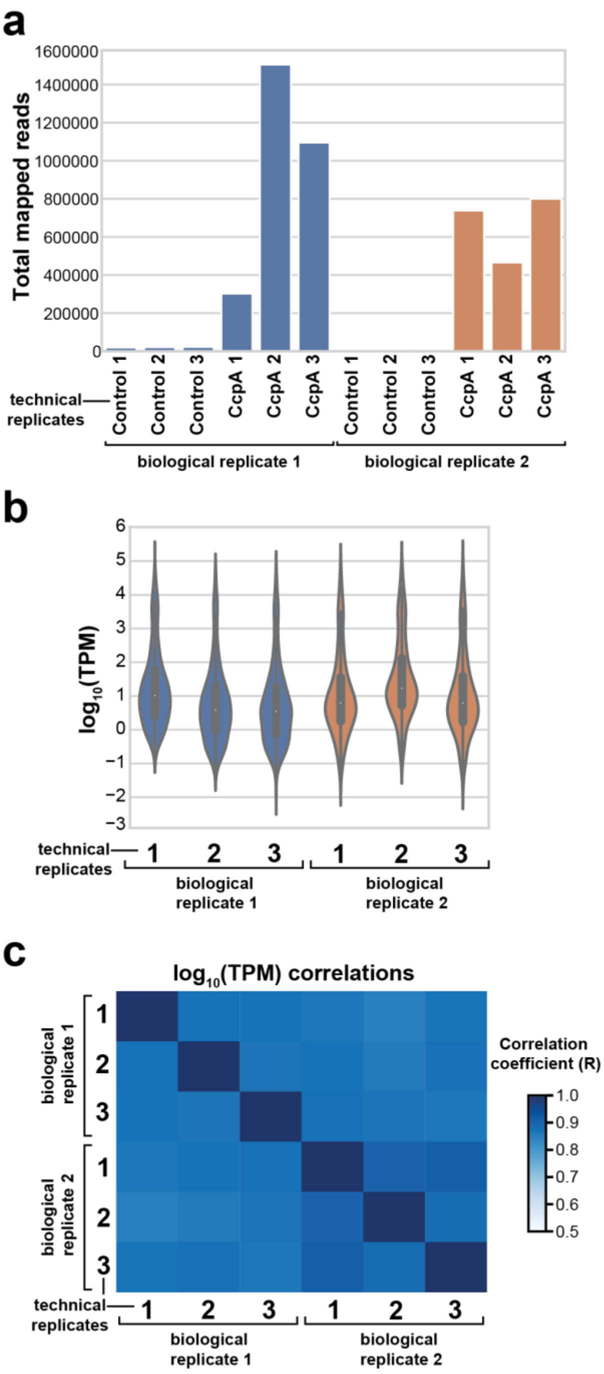

Supplementary Fig. 3. The CcpA CRAC data are highly reproducible and have very low background levels.

(a) Bar plot showing the total number of mapped reads identified in technical replicates of two biological replicate CcpA CRAC experiments. Each biological replicate experiment was performed by a different person.

(b) Distribution of Transcript Per Million (TPM) normalized read counts from the CcpA-HTF CRAC experiments (see Supplementary Table 2 for raw and TPM normalised counts of individual genes, box plots centre: median; minima/maxima: the minimum/maximum value in

the dataset excluding outliers; lower/upper hinges: the first/third quartiles; upper/lower whisker: from the hinge to the largest/ smallest value at most 1.5 * IQR (inter-quartile range) from the hinge; outliers: data beyond whiskers).

(c) Pearson correlation coefficients of comparisons between TPM normalised technical and biological replicates of the CcpA-HTF CRAC datasets.

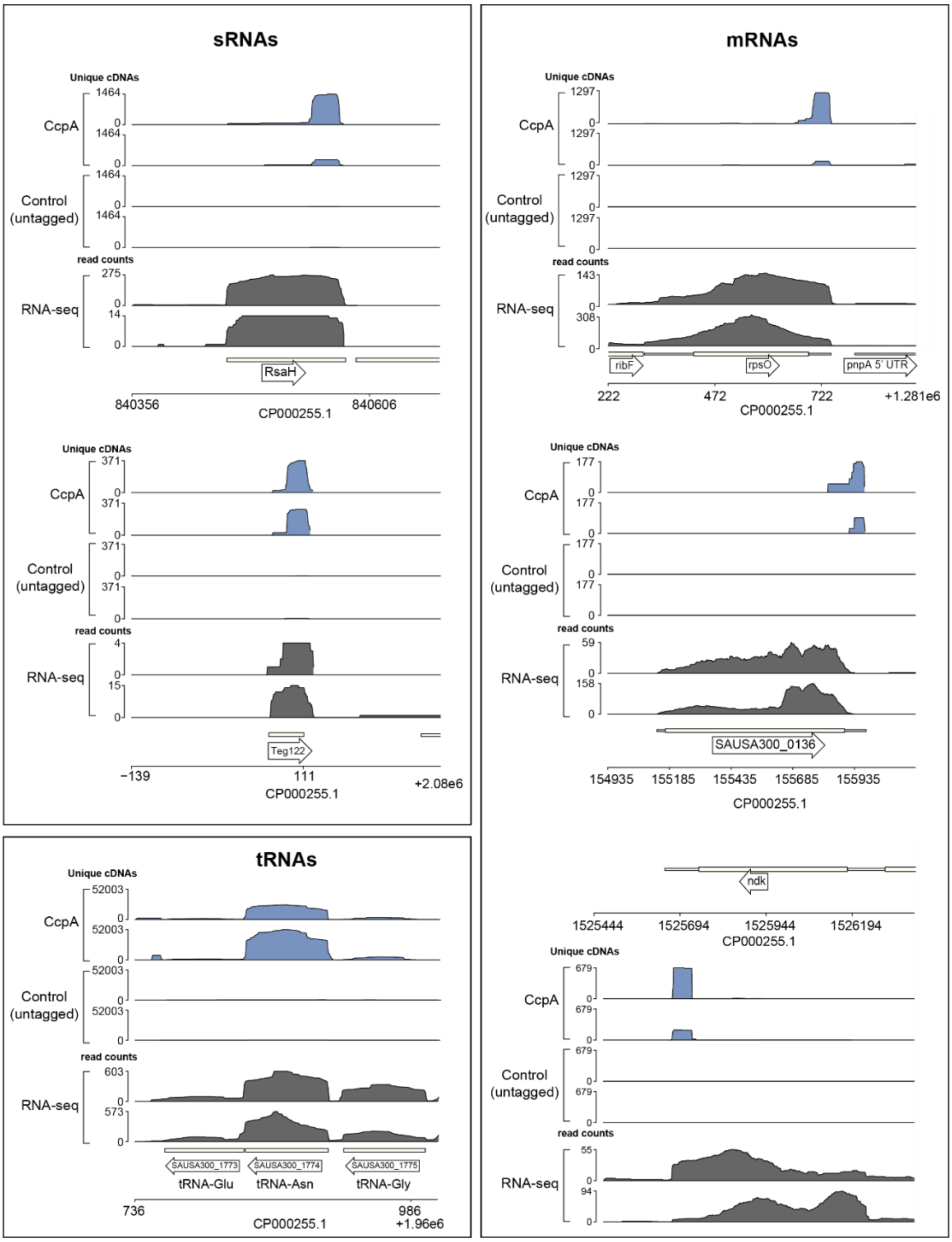

Supplementary Fig. 4. CcpA binds sRNAs and mRNAs near 3' ends of transcripts. Shown are examples of CcpA binding to diverse transcripts, including sRNAs, tRNAs and mRNAs. Shown are the results for two replicate CcpA-HTF CRAC experiments and two CRAC datasets that were generated from untagged cells. Also included are data from replicate RNA-seq experiments that were generated under the same conditions as the CRAC samples.

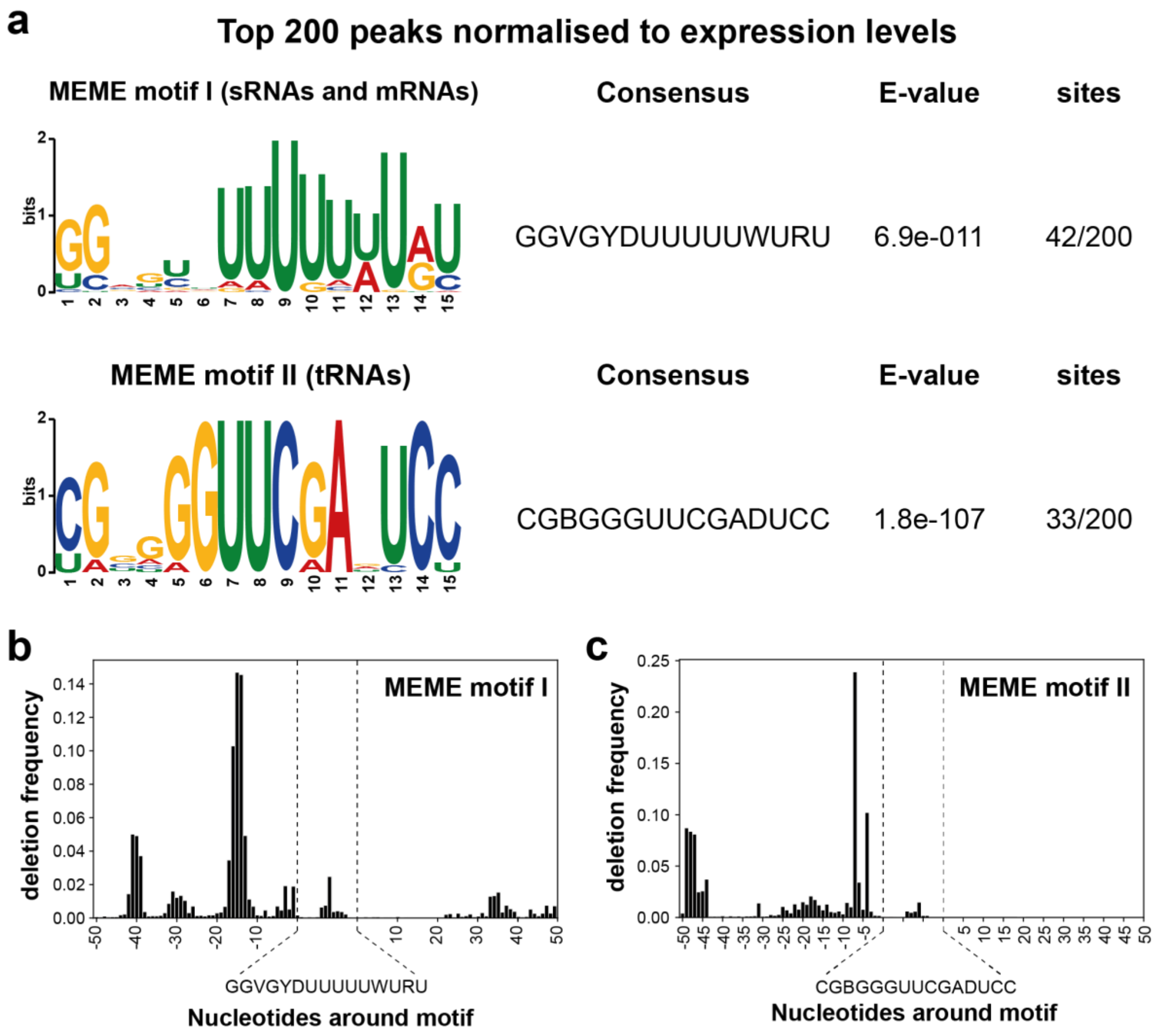

Supplementary Fig. 5. CcpA cross-links 10-15 nucleotides upstream of sequence motifs identified by MEME in CcpA binding sites.

(a) Motif analysis using the MEME suite[1] Motifs identified by MEME in the top 200 of the expression normalised peaks. The number of target sequences that contained the common motif and the E-value of MEME are shown.

(b-c) Distribution of nucleotide deletions (y-axis) found in reads around MEME motif I (b, x-axis) and MEME motif II (c, x-axis). The deletion frequency indicates the cumulative deletion frequency of deletions found in each read around the MEME motifs.

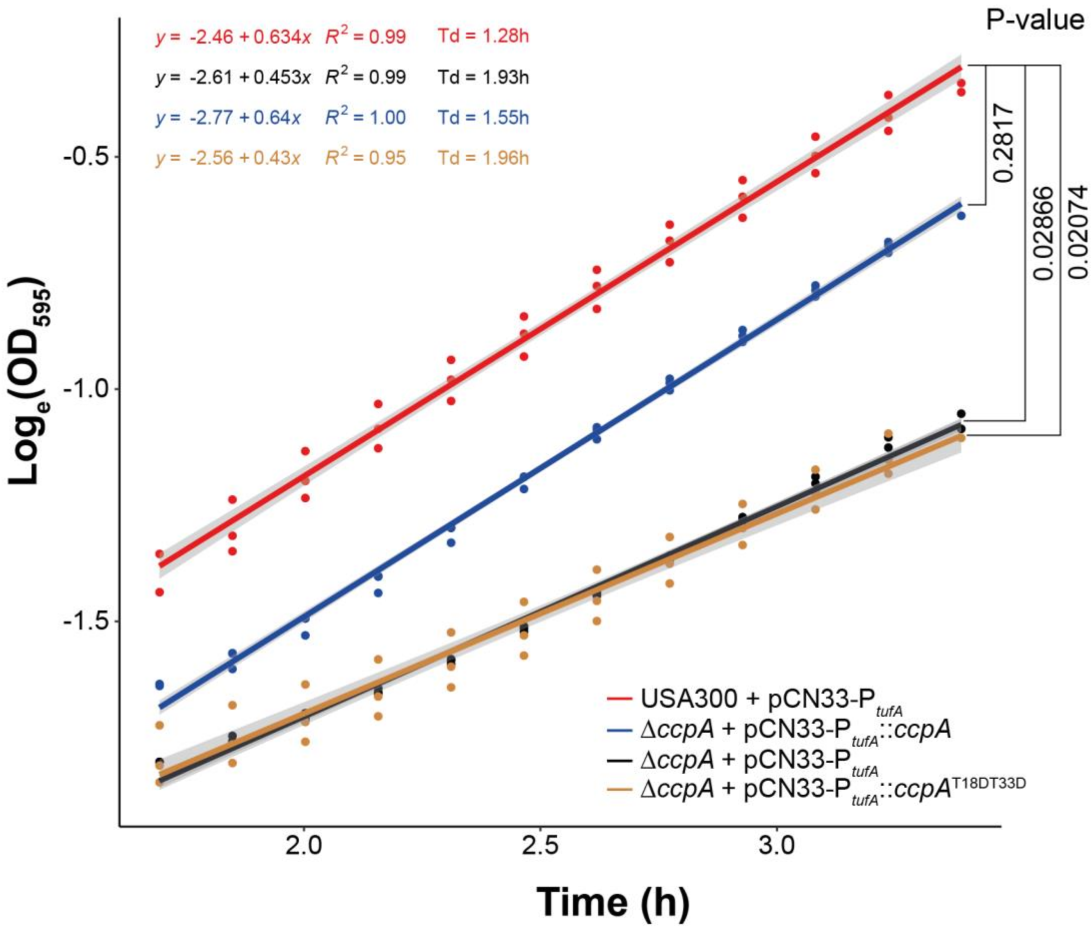

Supplementary Fig. 6. The CcpA T18DT33D mutant is unable to restore the growth defect of Δ*ccpA*.

Graphs represents log(e) of the OD_{595} values from 3 independent experimental replicates taken during the exponential phase of growth (0.16 – 0.32 on the unadjusted OD values from the plate reader). The thick lines represent the best fit linear regression, with the shaded area representing the 95% confidence interval. In the top left corner, the equations of the linear regression analyses are shown. 'Td': doubling time in hours. The unmodified p-values indicated in the figure were generated using a two-tailed Welch's T-test using the USA300 data between the interpolated time from OD 0.16 to 0.32 and the other samples.

Supplementary References

1. Bailey, T. L. *et al.* MEME Suite: Tools for motif discovery and searching. *Nucleic Acids Res.* **37**, (2009).
