# Supplementary material for: The RNA-bound proteome of MRSA reveals post-transcriptional roles for helix-turn-helix DNA-binding and Rossmann-fold proteins
Source: Nat Commun. 2022 May 24;13:2883. doi: 10.1038/s41467-022-30553-8 (PMC9130240; doi:10.1038/s41467-022-30553-8)
Supplement: Supplementary file 2 — Reporting Summary [file 41467_2022_30553_MOESM2_ESM.pdf]

nature portfolio

Corresponding author(s): Sander Granneman

Last updated by author(s): May 4, 2022

Reporting Summary

Nature Portfolio wishes to improve the reproducibility of the work that we publish. This form provides structure for consistency and transparency in reporting. For further information on Nature Portfolio policies, see our Editorial Policies and the Editorial Policy Checklist.

Statistics

For all statistical analyses, confirm that the following items are present in the figure legend, table legend, main text, or Methods section.

n/a	Confirmed	
☐	☒	The exact sample size (*n*) for each experimental group/condition, given as a discrete number and unit of measurement
☐	☒	A statement on whether measurements were taken from distinct samples or whether the same sample was measured repeatedly
☐	☒	The statistical test(s) used AND whether they are one- or two-sided<br>*Only common tests should be described solely by name; describe more complex techniques in the Methods section.*
☒	☐	A description of all covariates tested
☐	☒	A description of any assumptions or corrections, such as tests of normality and adjustment for multiple comparisons
☐	☒	A full description of the statistical parameters including central tendency (e.g. means) or other basic estimates (e.g. regression coefficient) AND variation (e.g. standard deviation) or associated estimates of uncertainty (e.g. confidence intervals)
☐	☒	For null hypothesis testing, the test statistic (e.g. *F*, *t*, *r*) with confidence intervals, effect sizes, degrees of freedom and *P* value noted<br>*Give P values as exact values whenever suitable.*
☒	☐	For Bayesian analysis, information on the choice of priors and Markov chain Monte Carlo settings
☒	☐	For hierarchical and complex designs, identification of the appropriate level for tests and full reporting of outcomes
☐	☒	Estimates of effect sizes (e.g. Cohen's *d*, Pearson's *r*), indicating how they were calculated

Our web collection on statistics for biologists contains articles on many of the points above.

Software and code

Policy information about availability of computer code

Data collection

MaxQuant version 1.6.2.10/ 1.6.10.4351 was used to process the mass-spectrometry data.

Data analysis

All the code we used for the data analyses are available from our repositories (https://git.ecdf.ed.ac.uk/sgrammen; https://pypi.org/user/g_ronimo/)
pyCRAC 1.5.1
CRAC_pipeline_PE 0.6.1

Other packages used:
ggplot2 (Wickham, 2016)
https://ggplot2.tidyverse.org/
dplyr (Wickham et al., 2019a)
https://dplyr.tidyverse.org/
readxl (Wickham et al., 2019b)
https://readxl.tidyverse.org/
writexl (Ooms, 2020)
https://docs.ropensci.org/writexl/
rowr (Varrichio, 2016)
https://www.rdocumentation.org/packages/rowr
reshape2 (Wickham, 2007)
https://github.com/hadley/reshape

superheat (Barter and Yu, 2018)
https://rlbarter.github.io/superheat/
tidyverse (Wickham et al., 2019c)
https://www.tidyverse.org/
tidyr (Wickham and Girlich, 2022)
https://tidyr.tidyverse.org/
ggrepel (Slowikowski et al., 2018)
https://cran.r-project.org/web/packages/ggrepel/vignettes/ggrepel.html
Hmisc (Harrell Jr et al., 2020)
https://hbiostat.org/R/Hmisc/
eulerr (Larsson et al., 2020)
https://cran.r-project.org/web/packages/eulerr/vignettes/introduction.html
stringr (Wickham, 2019)
https://stringr.tidyverse.org/
drawProteins (Brennan, 2018)
https://bioconductor.org/packages/release/bioc/html/drawProteins.html
Biostrings (Pagès et al., 2019)
https://bioconductor.org/packages/release/bioc/html/Biostrings.html
wordcloud (Fellows, 2018)
https://www.rdocumentation.org/packages/wordcloud/versions/2.6/topics/wordcloud
phylotools (Zhang, 2017)
https://github.com/helixcn/phylotools
limma (Ritchie et al., 2015)
https://bioconductor.org/packages/release/bioc/html/limma.html
qvalue (Storey et al., 2019)
https://www.bioconductor.org/packages/release/bioc/html/qvalue.html
PerformanceAnalytics (Brian G. Peterson, 2020)
https://cran.r-project.org/web/packages/PerformanceAnalytics/index.html
STRINGdb (Szklarczyk et al., 2019)
http://www.bioconductor.org/packages/release/bioc/html/STRINGdb.html
DMwR (Torgo, 2016)
http://cran.nexr.com/web/packages/DMwR/index.html
DESeq2 (Love et al., 2014)
https://bioconductor.org/packages/release/bioc/html/DESeq2.html
MaxQuant (1.6.2.10/ 1.6.10.43) (Cox and Mann, 2008)
https://www.maxquant.org/
RColorBrewer (Neuwirth, 2014)
https://cran.r-project.org/web/packages/RColorBrewer/index.html
genefilter (Gentleman et al., 2021)
https://bioconductor.org/packages/release/bioc/html/genefilter.html
pheatmap (Kolde, 2019)
https://www.rdocumentation.org/packages/pheatmap/versions/1.0.12/topics/pheatmap
EnhancedVolcano (Blighe et al., 2020)
https://bioconductor.org/packages/release/bioc/html/EnhancedVolcano.html
R (4.0.3) (R Core Team, 2020)
https://www.r-project.org/
RStudio (1.4.1103) (Team, 2015)
https://www.rstudio.com/

For manuscripts utilizing custom algorithms or software that are central to the research but not yet described in published literature, software must be made available to editors and reviewers. We strongly encourage code deposition in a community repository (e.g. GitHub). See the Nature Portfolio guidelines for submitting code & software for further information.

Data

Policy information about availability of data

All manuscripts must include a data availability statement. This statement should provide the following information, where applicable:

- Accession codes, unique identifiers, or web links for publicly available datasets
- A description of any restrictions on data availability
- For clinical datasets or third party data, please ensure that the statement adheres to our policy

The next generation sequencing data have been deposited on the NCBI Gene Expression Omnibus (GEO) with accession numbers GSE163719, GSE166151 and GSE189977. The mass spectrometry proteomics data have been deposited to the ProteomeXchange Consortium via the PRIDE58 partner repository with the dataset identifiers: 2C RBPs (JKD6009, PXD023368; USA300, PXD023427), 2C total lysate (PXD023408) and PTex RBPs and total lysate (PXD023414). The Source data are provided with this paper. Figure 6a was generated using pymol and PDB code 1RZR.

Field-specific reporting

Please select the one below that is the best fit for your research. If you are not sure, read the appropriate sections before making your selection.

[x] Life sciences   [ ] Behavioural & social sciences   [ ] Ecological, evolutionary & environmental sciences

For a reference copy of the document with all sections, see nature.com/documents/nr-reporting-summary-flat.pdf

Life sciences study design

All studies must disclose on these points even when the disclosure is negative.

Sample size	Experiments were performed using independent biological replicates (2-3). For the Mass-spectrometry, qPCR, RNA-seq and CLASH analyses at least two experimental replicates and/or three technical replicates were performed. No sample size calculation was performed. Sample sizes were chosen based on the minimal number of samples required to obtain robust statistics (RNA-seq: n>=3; label free mass-spectrometry n>=6; CRAC datasets n>=2)
Data exclusions	No data was excluded.
Replication	Experiments were performed at least twice and often by two independent researchers. To quantify reproducibility, we calculated Pearson correlations between experimental replicates. Where possible, results from each experimental replicate were displayed in either the main figures or the Supplementary Figures to illustrate reproducibility. We have also uploaded source files that show the Northern, Western and qPCR results for all experimental replicates.
Randomization	Not applicable as this study did not involve patients or clinical trials.
Blinding	Not applicable as this study did not involve patients or clinical trials.

Reporting for specific materials, systems and methods

We require information from authors about some types of materials, experimental systems and methods used in many studies. Here, indicate whether each material, system or method listed is relevant to your study. If you are not sure if a list item applies to your research, read the appropriate section before selecting a response.

Materials & experimental systems

n/a	Involved in the study	
[ ]	[x]	Antibodies
[x]	[ ]	Eukaryotic cell lines
[x]	[ ]	Palaeontology and archaeology
[x]	[ ]	Animals and other organisms
[x]	[ ]	Human research participants
[x]	[ ]	Clinical data
[x]	[ ]	Dual use research of concern

Methods

n/a	Involved in the study	
[x]	[ ]	ChIP-seq
[x]	[ ]	Flow cytometry
[x]	[ ]	MRI-based neuroimaging

Antibodies

Antibodies used	anti-FLAG-HRP antibody (clone M2; Sigma-Aldrich A8592)<br>anti-FLAG Magnetic Beads (clone M2; Sigma-Aldrich; M8823)<br>anti-TAP Tag polyclonal antibody (ThermoFisher; CAB1001)<br>goat anti-Rabbit IgG (Invitrogen; A16096)
Validation	Anti-FLAG antibodies:<br>https://www.sigmaaldrich.com/catalog/product/sigma/m8823?lang=en&region=GB<br><br>Anti-TAP antibody:<br>https://www.thermofisher.com/antibody/product/TAP-Tag-Antibody-Polyclonal/CAB1001
