# Supplementary material for: The RNA-bound proteome of MRSA reveals post-transcriptional roles for helix-turn-helix DNA-binding and Rossmann-fold proteins
Source: Nat Commun. 2022 May 24;13:2883. doi: 10.1038/s41467-022-30553-8 (PMC9130240; doi:10.1038/s41467-022-30553-8)
Supplement: Supplementary file 3 — Description of Additional Supplementary Files [file 41467_2022_30553_MOESM3_ESM.pdf]

Title: Supplementary Data 1
Description: (PRIDE file sample descriptions): Sample information of data submitted to PRIDE. (all others): RBPs identified in JKD6009 (2C), USA300 LPM (2C), USA300 TSB (2C) and USA300 (PTex).

Title: Supplementary Data 2.
Description: CcpA CRAC raw counts replicate 1 and 2.

Title: Supplementary Data 3:
Description: DESeq2 analyses of the CcpA CRAC data (n=6; two independent biological replicates with three technical replicates each) compared to RNA-seq data generated under the same conditions (n=3; three independent biological replicates).

Title: Supplementary Data 4:
Description: BEAM and MEME motifs found in the top 200 CcpA binding peaks identified by CRAC (two motifs each).

Title: Supplementary Data 5:
Description: DESeq2 differential expression analysis of USA300 wild-type, Δ*ccpA* samples and the Δ*ccpA* strain complemented with CcpA expressed from a plasmid (Δ*ccpA* + pCN33-P_{tufA}::*ccpA*).

Title: Supplementary Data 6:
Description: Plasmids, DNA fragments, oligonucleotides and bacterial strains used in this study.
