# Supplementary material for: The RNA-bound proteome of MRSA reveals post-transcriptional roles for helix-turn-helix DNA-binding and Rossmann-fold proteins
Source: Nat Commun. 2022 May 24;13:2883. doi: 10.1038/s41467-022-30553-8 (PMC9130240; doi:10.1038/s41467-022-30553-8)
Supplement: Supplementary file 10 — Source Data [file 41467_2022_30553_MOESM10_ESM.zip › Source_Data/Source_Data_Growth_curves_Fig_6b/platereadr_ccpA.nb.html]

Growth Curve Analysis


Code 

- Show All Code
- Hide All Code
- Download Rmd

# Growth Curve Analysis

This is an r-notebook to take the output file of the i-control software and create a growth-curve from this. Input is a csv of the excel sheet produced by the program.


```
library(dplyr)
library(tidyr)
library(reshape2)
library(ggplot2)
library(reticulate)
conda_create("r-reticulate")
```


```
Collecting package metadata (current_repodata.json): ...working... done
Solving environment: ...working... done

## Package Plan ##

  environment location: /Users/hmccaugh/Library/r-miniconda/envs/r-reticulate

  added / updated specs:
    - python


The following NEW packages will be INSTALLED:

  ca-certificates    conda-forge/osx-64::ca-certificates-2021.5.30-h033912b_0
  libcxx             conda-forge/osx-64::libcxx-12.0.1-habf9029_0
  libffi             conda-forge/osx-64::libffi-3.4.2-he49afe7_4
  ncurses            conda-forge/osx-64::ncurses-6.2-h2e338ed_4
  openssl            conda-forge/osx-64::openssl-3.0.0-h0d85af4_1
  pip                conda-forge/noarch::pip-21.2.4-pyhd8ed1ab_0
  python             conda-forge/osx-64::python-3.9.7-h38b4d05_3_cpython
  python_abi         conda-forge/osx-64::python_abi-3.9-2_cp39
  readline           conda-forge/osx-64::readline-8.1-h05e3726_0
  setuptools         conda-forge/osx-64::setuptools-58.0.4-py39h6e9494a_2
  sqlite             conda-forge/osx-64::sqlite-3.36.0-h23a322b_2
  tk                 conda-forge/osx-64::tk-8.6.11-h5dbffcc_1
  tzdata             conda-forge/noarch::tzdata-2021a-he74cb21_1
  wheel              conda-forge/noarch::wheel-0.37.0-pyhd8ed1ab_1
  xz                 conda-forge/osx-64::xz-5.2.5-haf1e3a3_1
  zlib               conda-forge/osx-64::zlib-1.2.11-h7795811_1010


Preparing transaction: ...working... done
Verifying transaction: ...working... done
Executing transaction: ...working... done
#
# To activate this environment, use
#
#     $ conda activate r-reticulate
#
# To deactivate an active environment, use
#
#     $ conda deactivate

[1] "/Users/hmccaugh/Library/r-miniconda/envs/r-reticulate/bin/python"
```


```
py_install("pandas")
```


```
Collecting package metadata (current_repodata.json): ...working... done
Solving environment: ...working... done

## Package Plan ##

  environment location: /Users/hmccaugh/Library/r-miniconda/envs/r-reticulate

  added / updated specs:
    - pandas


The following NEW packages will be INSTALLED:

  libblas            conda-forge/osx-64::libblas-3.9.0-11_osx64_openblas
  libcblas           conda-forge/osx-64::libcblas-3.9.0-11_osx64_openblas
  libgfortran        conda-forge/osx-64::libgfortran-5.0.0-9_3_0_h6c81a4c_23
  libgfortran5       conda-forge/osx-64::libgfortran5-9.3.0-h6c81a4c_23
  liblapack          conda-forge/osx-64::liblapack-3.9.0-11_osx64_openblas
  libopenblas        conda-forge/osx-64::libopenblas-0.3.17-openmp_h3351f45_1
  llvm-openmp        conda-forge/osx-64::llvm-openmp-12.0.1-hda6cdc1_1
  numpy              conda-forge/osx-64::numpy-1.21.2-py39h7eed0ac_0
  pandas             conda-forge/osx-64::pandas-1.3.3-py39h4d6be9b_0
  python-dateutil    conda-forge/noarch::python-dateutil-2.8.2-pyhd8ed1ab_0
  pytz               conda-forge/noarch::pytz-2021.1-pyhd8ed1ab_0
  six                conda-forge/noarch::six-1.16.0-pyh6c4a22f_0


Preparing transaction: ...working... done
Verifying transaction: ...working... done
Executing transaction: ...working... done
```


```
py_install("numpy")
```


```
Collecting package metadata (current_repodata.json): ...working... done
Solving environment: ...working... done

# All requested packages already installed.
```


```
pip install tkintertable
```


```
Collecting tkintertable
  Using cached tkintertable-1.3.3-py3-none-any.whl
Collecting future
  Using cached future-0.18.2-py3-none-any.whl
Installing collected packages: future, tkintertable
Successfully installed future-0.18.2 tkintertable-1.3.3
```


```
from tkintertable import TableCanvas, TableModel, Prefs, Tables_IO
from tkinter import *
import csv as csv
import tkinter as tk
import random
from collections import OrderedDict
import pandas as pd
import numpy as np
data={"A": {'1_Var': "A1 Variable", '1_Rep': "A1 Replicate", '2_Var': "A2 Variable", '2_Rep': "A2 Replicate",'3_Var': "A3 Variable", '3_Rep': "A3 Replicate", '4_Var': "A4 Variable", '4_Rep': "A4 Replicate",'5_Var': "A5 Variable", '5_Rep': "A5 Replicate", '6_Var': "A6 Variable", '6_Rep':"A6 Replicate", '7_Var':"A7 Variable",'7_Rep': "A7 Replicate",'8_Var': "A8 Variable", '8_Rep': "A8 Replicate", '9_Var': "A9 Variable", '9_Rep':"A9 Replicate", '10_Var':"A10 Variable", '10_Rep': "A10 Replicate",'11_Var': "A11 Variable", '11_Rep': "A11 Replicate", '12_Var': "A12 Variable", '12_Rep': "A12 Replicate"},
 "B": {'1_Var': "B1 Variable", '1_Rep': "B1 Replicate", '2_Var': "B2 Variable", '2_Rep': "B2 Replicate",'3_Var': "B3 Variable", '3_Rep': "B3 Replicate", '4_Var': "B4 Variable", '4_Rep': "B4 Replicate",'5_Var': "B5 Variable", '5_Rep': "B5 Replicate", '6_Var': "B6 Variable", '6_Rep':"A6 Replicate", '7_Var':"A7 Variable",'7_Rep': "B7 Replicate",'8_Var': "B8 Variable", '8_Rep': "B8 Replicate", '9_Var': "B9 Variable", '9_Rep':"A9 Replicate", '10_Var':"A10 Variable",'10_Rep': "B10 Replicate",'11_Var': "B11 Variable", '11_Rep': "B11 Replicate", '12_Var': "B12 Variable", '12_Rep': "B12 Replicate"},
 "C": {'1_Var': "C1 Variable", '1_Rep': "C1 Replicate", '2_Var': "C2 Variable", '2_Rep': "C2 Replicate",'3_Var': "C3 Variable", '3_Rep': "C3 Replicate", '4_Var': "C4 Variable", '4_Rep': "C4 Replicate",'5_Var': "C5 Variable", '5_Rep': "C5 Replicate", '6_Var': "C6 Variable", '6_Rep':"A6 Replicate", '7_Var':"A7 Variable",'7_Rep': "C7 Replicate",'8_Var': "C8 Variable", '8_Rep': "C8 Replicate", '9_Var': "C9 Variable", '9_Rep':"A9 Replicate", '10_Var':"A10 Variable",'10_Rep': "C10 Replicate",'11_Var': "C11 Variable", '11_Rep': "C11 Replicate", '12_Var': "C12 Variable", '12_Rep': "C12 Replicate"},
 "D": {'1_Var': "D1 Variable", '1_Rep': "D1 Replicate", '2_Var': "D2 Variable", '2_Rep': "D2 Replicate",'3_Var': "D3 Variable", '3_Rep': "D3 Replicate", '4_Var': "D4 Variable", '4_Rep': "D4 Replicate",'5_Var': "D5 Variable", '5_Rep': "D5 Replicate", '6_Var': "D6 Variable", '6_Rep':"A6 Replicate", '7_Var':"A7 Variable",'7_Rep': "D7 Replicate",'8_Var': "D8 Variable", '8_Rep': "D8 Replicate", '9_Var': "D9 Variable", '9_Rep':"A9 Replicate", '10_Var':"A10 Variable",'10_Rep': "D10 Replicate",'11_Var': "D11 Variable", '11_Rep': "D11 Replicate", '12_Var': "D12 Variable", '12_Rep': "D12 Replicate"},
 "E": {'1_Var': "E1 Variable", '1_Rep': "E1 Replicate", '2_Var': "E2 Variable", '2_Rep': "E2 Replicate",'3_Var': "E3 Variable", '3_Rep': "E3 Replicate", '4_Var': "E4 Variable", '4_Rep': "E4 Replicate",'5_Var': "E5 Variable", '5_Rep': "E5 Replicate", '6_Var': "E6 Variable", '6_Rep':"A6 Replicate", '7_Var':"A7 Variable",'7_Rep': "E7 Replicate",'8_Var': "E8 Variable", '8_Rep': "E8 Replicate", '9_Var': "E9 Variable", '9_Rep':"A9 Replicate", '10_Var':"A10 Variable",'10_Rep': "E10 Replicate",'11_Var': "E11 Variable", '11_Rep': "E11 Replicate", '12_Var': "E12 Variable", '12_Rep': "E12 Replicate"},
 "F": {'1_Var': "F1 Variable", '1_Rep': "F1 Replicate", '2_Var': "F2 Variable", '2_Rep': "F2 Replicate",'3_Var': "F3 Variable", '3_Rep': "F3 Replicate", '4_Var': "F4 Variable", '4_Rep': "F4 Replicate",'5_Var': "F5 Variable", '5_Rep': "F5 Replicate", '6_Var': "F6 Variable", '6_Rep':"A6 Replicate", '7_Var':"A7 Variable",'7_Rep': "F7 Replicate",'8_Var': "F8 Variable", '8_Rep': "F8 Replicate", '9_Var': "F9 Variable", '9_Rep':"A9 Replicate", '10_Var':"A10 Variable",'10_Rep': "F10 Replicate",'11_Var': "F11 Variable", '11_Rep': "F11 Replicate", '12_Var': "F12 Variable", '12_Rep': "F12 Replicate"},
 "G": {'1_Var': "G1 Variable", '1_Rep': "G1 Replicate", '2_Var': "G2 Variable", '2_Rep': "G2 Replicate",'3_Var': "G3 Variable", '3_Rep': "G3 Replicate", '4_Var': "G4 Variable", '4_Rep': "G4 Replicate",'5_Var': "G5 Variable", '5_Rep': "G5 Replicate", '6_Var': "G6 Variable", '6_Rep':"A6 Replicate", '7_Var':"A7 Variable",'7_Rep': "G7 Replicate",'8_Var': "G8 Variable", '8_Rep': "G8 Replicate", '9_Var': "G9 Variable", '9_Rep':"A9 Replicate", '10_Var':"A10 Variable",'10_Rep': "G10 Replicate",'11_Var': "G11 Variable", '11_Rep': "G11 Replicate", '12_Var': "G12 Variable", '12_Rep': "G12 Replicate"},
 "H": {'1_Var': "H1 Variable", '1_Rep': "H1 Replicate", '2_Var': "H2 Variable", '2_Rep': "H2 Replicate",'3_Var': "H3 Variable", '3_Rep': "H3 Replicate", '4_Var': "H4 Variable", '4_Rep': "H4 Replicate",'5_Var': "H5 Variable", '5_Rep': "H5 Replicate", '6_Var': "H6 Variable", '6_Rep':"A6 Replicate", '7_Var':"A7 Variable",'7_Rep': "H7 Replicate",'8_Var': "H8 Variable", '8_Rep': "H8 Replicate", '9_Var': "H9 Variable", '9_Rep':"A9 Replicate", '10_Var':"A10 Variable",'10_Rep': "H10 Replicate",'11_Var': "H11 Variable", '11_Rep': "H11 Replicate", '12_Var': "H12 Variable", '12_Rep': "H12 Replicate"}}
```


```
class PlateMapMaker(Frame):
    def __init__(self, parent=None):
        self.parent = parent
        global table
        Frame.__init__(self)
        self.main = self.master
        self.main.geometry('1800x400')
        self.main.title('Platemap')
        f = Frame(self.main)
        f.pack(fill=BOTH,expand=1)
        table = TableCanvas(f, data=data, showkeynamesinheader=True, rowheight=40, thefont=("",12))
        table.redraw
        table.update()
        btn2 = tk.Button(table, text="Export as CSV for platereadr", command = save_platemap)
        table.create_window((900,330), window=btn2) 
        table.show()
        return
    
def save_platemap():
        from tkintertable.Tables_IO import TableExporter
        exporter = TableExporter()
        exporter.ExportTableData(table)
        return

def save_4_r(filename = "platereader_key.csv"):
        pl_df.to_csv(filename)
```


```
app=PlateMapMaker()
app.mainloop()
```


```
app.quit()
```


```
        variable      replicate cell
0         aaaaaa              1   A1
1         aaaaaa              2   B1
2         aaaaaa              3   C1
3         bbbbbb              1   D1
4         bbbbbb              2   E1
..           ...            ...  ...
91  D12 Variable  D12 Replicate  D12
92  E12 Variable  E12 Replicate  E12
93  F12 Variable  F12 Replicate  F12
94  G12 Variable  G12 Replicate  G12
95  H12 Variable  H12 Replicate  H12

[96 rows x 3 columns]
```


```
#Variables (n is the number of replicates of each condition, wells)
```


```
The working directory was changed to /Users/hmccaugh inside a notebook chunk. The working directory will be reset when the chunk is finished running. Use the knitr root.dir option in the setup chunk to change the working directory for notebook chunks.
```


```
input<-"~/Documents/new_format/programming/platereadr/9501_dev_version/20210928_v2/test_len17.csv"
timepoints<-60
wells<-17 #Need to set the number of rows in the raw data
```


```
#import platemap key from python script
platemap<-"~/Documents/new_format/programming/platereadr/9501_dev_version/20210928_v2/platereader_key.csv"
platemap_in<-read.csv(platemap)
```


```
#Import the file as a dataframe
raw_df<-read.csv(input, header = FALSE, stringsAsFactors=FALSE)

#Create vector of the Time
time_Labels<-raw_df[38,2:(timepoints+1)]

#Create vector of the Temp
temp_labels<-raw_df[39,]

#Create vector of the Cycle number
cycle_labels<-raw_df[37,]

#Trim the dataframe
clean_df<-raw_df[40:(39+wells),1:(timepoints+1)]

#Assign correct row names
row.names(clean_df)<-clean_df[,1]

#Remove row names column
cleanest_df<-clean_df[,1:ncol(clean_df)]

#get rid of horrible factors
cleanest_df$V2<-as.numeric(as.character(cleanest_df$V2))
cleanest_df$V4<-as.numeric(as.character(cleanest_df$V4))
cleanest_df$V5<-as.numeric(as.character(cleanest_df$V5))
cleanest_df$V6<-as.numeric(as.character(cleanest_df$V6))
cleanest_df$V8<-as.numeric(as.character(cleanest_df$V8))
cleanest_df$V9<-as.numeric(as.character(cleanest_df$V9))
```


```
#filter data to include only those which appear in the key
input_used <- left_join(platemap_in,cleanest_df,by = c("cell" = "V1"))
input_used <- input_used[,2:ncol(input_used)]
input_used <- filter(input_used, !grepl("Variable", variable))
```


```
#filter data to include only those which appear in the key
input_used <- left_join(platemap_in,cleanest_df,by = c("cell" = "V1"))
input_used <- input_used[,2:ncol(input_used)]
input_used <- filter(input_used, !grepl("Variable", variable))
```


```
#make a list of unique variables
variables_list<-input_used$variable[!duplicated(input_used$variable)]
```


```
replicates <- list()
#make dfs for the variables
for (i in variables_list){
  a <- (filter(input_used, input_used$variable == i))
  a <- a[,4:ncol(a)]
  replicates[[i]] <- a
  }
```


```
#Split replicates into separate dataframes held in a list
#nr <- nrow(cleanest_df)
#replicates<-split(cleanest_df, rep(1:ceiling(nr/n), each=n, length.out=nr))

#Calculate mean and standard deviation for each data point
means<-mapply(colMeans, replicates) 
colSdThomas <- function(x)sqrt(rowMeans(na.rm=TRUE, (t(x)-colMeans(x, na.rm=TRUE))^2)*((dim(x)[1])/(dim(x)[1]-1)))
sdevs<-mapply(colSdThomas, replicates)
```


```
#Append these to the dataframes
e<-1
rep_list<-list()
for(x in (1:length(variables_list))){
  rep<-rbind(replicates[[x]], (t(means[,x])),(t(sdevs[,x])),((means[,x])+(2*sdevs[,x])), (means[,x]-(2*sdevs[,x])))
  rep_list[[e]]<-rep
  e<-e+1
}
```


```
#Name the new rows
j<-1
for(x in rep_list){
  row.names(rep_list[[j]])[(nrow(replicates[[j]])+1)]<-"mean"
  row.names(rep_list[[j]])[(nrow(replicates[[j]])+2)]<-"stdev"
  row.names(rep_list[[j]])[(nrow(replicates[[j]])+3)]<-"upperbound"
  row.names(rep_list[[j]])[(nrow(replicates[[j]])+4)]<-"lowerbound"
  j<-j+1
}
```


```
#Split out the time labels and move to hours format
time_labels<-as.numeric(time_Labels)
timepoints_h<-time_labels/3600
timepoints_h <- c("Time (h)", timepoints_h)
```


```
#CALCULATE OUTLIERS???
```


```
#get means
means_out = list()
upper_error = list()
lower_error = list()

for (i in 1:length(rep_list)){
  means_out[[i]]<-rep_list[[i]][4,]
  upper_error[[i]]<-rep_list[[i]][6,]
  lower_error[[i]]<-rep_list[[i]][7,]
}

names(rep_list)<-variables_list
names(means_out)<-variables_list
names(upper_error)<-variables_list
names(lower_error)<-variables_list

means_fin<-bind_rows(means_out, .id = "variables")
upper_error_fin<-bind_rows(upper_error, .id = 'variables')
lower_error_fin<-bind_rows(lower_error, .id = 'variables')
```


```
rep_list[1]
```


```
$aaaaaa
```


```
NA
```


```
means_final<-rbind.data.frame(means_fin, timepoints_h)
means_final_2<-(means_final[,-1])
rownames(means_final_2)<-means_final[,1]
means_final_2<-as.data.frame(t(means_final_2))


upper_final<-rbind.data.frame(upper_error_fin, timepoints_h)
upper_final_2<-upper_final[,-1]
rownames(upper_final_2)<-upper_final[,1]
upper_final_2<-as.data.frame(t(upper_final_2))

lower_final<-rbind.data.frame(lower_error_fin, timepoints_h)
lower_final_2<-lower_final[,-1]
rownames(lower_final_2)<-lower_final[,1]
lower_final_2<-as.data.frame(t(lower_final_2))
```


```
#Make the dataframe for a long format
long_df <- data.frame(OD595=integer(), Time=integer(), SDev=integer(), Sample=character(), stringsAsFactors = FALSE)

#Get a list of names for the assignment of the names
sample_names <- colnames(means_final_2)
```


```
#make the long dataframe
for (i in (1:(length(variables_list)))){
  new_df <- data.frame(means_final_2[i], means_final_2$Time, lower_final_2[i], upper_final_2[i], variables_list[i])
  colnames(new_df) <- c("OD595", "Time", "Lower", "Upper", "Sample")
  long_df <- rbind.data.frame(long_df, new_df)
}
```


```
#use the sample names to make factor levels - user can create this list to get consistent colours...
```


```
Warning message:
In readChar(file, size, TRUE) : truncating string with embedded nuls
```


```
long_df$Sample<-factor(long_df$Sample, levels = variables_list)
graph_colours<-c("red", "black","blue","peru","brown4","magenta","turquoise3","green","khaki","#black")
graph_line_opacity <- c(1,1,1,1,1,1,1,1,1)
gph_1_labels_df <- data.frame(variables_list)
gph_1_labels_df$opacity <- graph_line_opacity
gph_1_labels_df <- gph_1_labels_df[(!gph_1_labels_df$opacity == 0),]
gph_1_labels_list <-gph_1_labels_df$variables_list
```


```
#x = 1:(total_gph_number)
#gph_titles<-paste0("gph_title_", x)
#gph_variables<-paste0("gph_variables_", x)
#gph_colours<-paste0("gph_variables_", x)
```


```
#for (i in 1:(total_gph_number)){
#  gph_lines<- long_df %>% filter(Sample %in% gph_variables[[i]])
#  print(gph_lines)
#}
```


```
long_df$OD595 <- as.numeric(long_df$OD595)
long_df$Time<- as.numeric(long_df$Time)
long_df$Lower<- as.numeric(long_df$Lower)
long_df$Upper<- as.numeric(long_df$Upper)
```


```
#Input fields to make graph 1 programatically
gph_title_1<-"Graph 1"
gph_variables_1<-variables_list
gph_colours_1<-graph_colours


#Input to make graph 2 programatically
gph_title_2 <- "Graph 2"
gph_variables_2 <- variables_list[2:4]
gph_colours_2 <- c("black","yellow","red")

total_gph_number <- 2
```


LS0tCnRpdGxlOiAiR3Jvd3RoIEN1cnZlIEFuYWx5c2lzIgpvdXRwdXQ6IGh0bWxfbm90ZWJvb2sKLS0tCgpUaGlzIGlzIGFuIHItbm90ZWJvb2sgdG8gdGFrZSB0aGUgb3V0cHV0IGZpbGUgb2YgdGhlIGktY29udHJvbCBzb2Z0d2FyZSBhbmQgY3JlYXRlIGEgZ3Jvd3RoLWN1cnZlIGZyb20gdGhpcy4KSW5wdXQgaXMgYSBjc3Ygb2YgdGhlIGV4Y2VsIHNoZWV0IHByb2R1Y2VkIGJ5IHRoZSBwcm9ncmFtLgoKYGBge3J9CmxpYnJhcnkoZHBseXIpCmxpYnJhcnkodGlkeXIpCmxpYnJhcnkocmVzaGFwZTIpCmxpYnJhcnkoZ2dwbG90MikKbGlicmFyeShyZXRpY3VsYXRlKQpjb25kYV9jcmVhdGUoInItcmV0aWN1bGF0ZSIpCnB5X2luc3RhbGwoInBhbmRhcyIpCnB5X2luc3RhbGwoIm51bXB5IikKYGBgCgpgYGB7YmFzaH0KcGlwIGluc3RhbGwgdGtpbnRlcnRhYmxlCgpgYGAKCmBgYHtweXRob259CmZyb20gdGtpbnRlcnRhYmxlIGltcG9ydCBUYWJsZUNhbnZhcywgVGFibGVNb2RlbCwgUHJlZnMsIFRhYmxlc19JTwpmcm9tIHRraW50ZXIgaW1wb3J0ICoKaW1wb3J0IGNzdiBhcyBjc3YKaW1wb3J0IHRraW50ZXIgYXMgdGsKaW1wb3J0IHJhbmRvbQpmcm9tIGNvbGxlY3Rpb25zIGltcG9ydCBPcmRlcmVkRGljdAppbXBvcnQgcGFuZGFzIGFzIHBkCmltcG9ydCBudW1weSBhcyBucApkYXRhPXsiQSI6IHsnMV9WYXInOiAiQTEgVmFyaWFibGUiLCAnMV9SZXAnOiAiQTEgUmVwbGljYXRlIiwgJzJfVmFyJzogIkEyIFZhcmlhYmxlIiwgJzJfUmVwJzogIkEyIFJlcGxpY2F0ZSIsJzNfVmFyJzogIkEzIFZhcmlhYmxlIiwgJzNfUmVwJzogIkEzIFJlcGxpY2F0ZSIsICc0X1Zhcic6ICJBNCBWYXJpYWJsZSIsICc0X1JlcCc6ICJBNCBSZXBsaWNhdGUiLCc1X1Zhcic6ICJBNSBWYXJpYWJsZSIsICc1X1JlcCc6ICJBNSBSZXBsaWNhdGUiLCAnNl9WYXInOiAiQTYgVmFyaWFibGUiLCAnNl9SZXAnOiJBNiBSZXBsaWNhdGUiLCAnN19WYXInOiJBNyBWYXJpYWJsZSIsJzdfUmVwJzogIkE3IFJlcGxpY2F0ZSIsJzhfVmFyJzogIkE4IFZhcmlhYmxlIiwgJzhfUmVwJzogIkE4IFJlcGxpY2F0ZSIsICc5X1Zhcic6ICJBOSBWYXJpYWJsZSIsICc5X1JlcCc6IkE5IFJlcGxpY2F0ZSIsICcxMF9WYXInOiJBMTAgVmFyaWFibGUiLCAnMTBfUmVwJzogIkExMCBSZXBsaWNhdGUiLCcxMV9WYXInOiAiQTExIFZhcmlhYmxlIiwgJzExX1JlcCc6ICJBMTEgUmVwbGljYXRlIiwgJzEyX1Zhcic6ICJBMTIgVmFyaWFibGUiLCAnMTJfUmVwJzogIkExMiBSZXBsaWNhdGUifSwKICJCIjogeycxX1Zhcic6ICJCMSBWYXJpYWJsZSIsICcxX1JlcCc6ICJCMSBSZXBsaWNhdGUiLCAnMl9WYXInOiAiQjIgVmFyaWFibGUiLCAnMl9SZXAnOiAiQjIgUmVwbGljYXRlIiwnM19WYXInOiAiQjMgVmFyaWFibGUiLCAnM19SZXAnOiAiQjMgUmVwbGljYXRlIiwgJzRfVmFyJzogIkI0IFZhcmlhYmxlIiwgJzRfUmVwJzogIkI0IFJlcGxpY2F0ZSIsJzVfVmFyJzogIkI1IFZhcmlhYmxlIiwgJzVfUmVwJzogIkI1IFJlcGxpY2F0ZSIsICc2X1Zhcic6ICJCNiBWYXJpYWJsZSIsICc2X1JlcCc6IkE2IFJlcGxpY2F0ZSIsICc3X1Zhcic6IkE3IFZhcmlhYmxlIiwnN19SZXAnOiAiQjcgUmVwbGljYXRlIiwnOF9WYXInOiAiQjggVmFyaWFibGUiLCAnOF9SZXAnOiAiQjggUmVwbGljYXRlIiwgJzlfVmFyJzogIkI5IFZhcmlhYmxlIiwgJzlfUmVwJzoiQTkgUmVwbGljYXRlIiwgJzEwX1Zhcic6IkExMCBWYXJpYWJsZSIsJzEwX1JlcCc6ICJCMTAgUmVwbGljYXRlIiwnMTFfVmFyJzogIkIxMSBWYXJpYWJsZSIsICcxMV9SZXAnOiAiQjExIFJlcGxpY2F0ZSIsICcxMl9WYXInOiAiQjEyIFZhcmlhYmxlIiwgJzEyX1JlcCc6ICJCMTIgUmVwbGljYXRlIn0sCiAiQyI6IHsnMV9WYXInOiAiQzEgVmFyaWFibGUiLCAnMV9SZXAnOiAiQzEgUmVwbGljYXRlIiwgJzJfVmFyJzogIkMyIFZhcmlhYmxlIiwgJzJfUmVwJzogIkMyIFJlcGxpY2F0ZSIsJzNfVmFyJzogIkMzIFZhcmlhYmxlIiwgJzNfUmVwJzogIkMzIFJlcGxpY2F0ZSIsICc0X1Zhcic6ICJDNCBWYXJpYWJsZSIsICc0X1JlcCc6ICJDNCBSZXBsaWNhdGUiLCc1X1Zhcic6ICJDNSBWYXJpYWJsZSIsICc1X1JlcCc6ICJDNSBSZXBsaWNhdGUiLCAnNl9WYXInOiAiQzYgVmFyaWFibGUiLCAnNl9SZXAnOiJBNiBSZXBsaWNhdGUiLCAnN19WYXInOiJBNyBWYXJpYWJsZSIsJzdfUmVwJzogIkM3IFJlcGxpY2F0ZSIsJzhfVmFyJzogIkM4IFZhcmlhYmxlIiwgJzhfUmVwJzogIkM4IFJlcGxpY2F0ZSIsICc5X1Zhcic6ICJDOSBWYXJpYWJsZSIsICc5X1JlcCc6IkE5IFJlcGxpY2F0ZSIsICcxMF9WYXInOiJBMTAgVmFyaWFibGUiLCcxMF9SZXAnOiAiQzEwIFJlcGxpY2F0ZSIsJzExX1Zhcic6ICJDMTEgVmFyaWFibGUiLCAnMTFfUmVwJzogIkMxMSBSZXBsaWNhdGUiLCAnMTJfVmFyJzogIkMxMiBWYXJpYWJsZSIsICcxMl9SZXAnOiAiQzEyIFJlcGxpY2F0ZSJ9LAogIkQiOiB7JzFfVmFyJzogIkQxIFZhcmlhYmxlIiwgJzFfUmVwJzogIkQxIFJlcGxpY2F0ZSIsICcyX1Zhcic6ICJEMiBWYXJpYWJsZSIsICcyX1JlcCc6ICJEMiBSZXBsaWNhdGUiLCczX1Zhcic6ICJEMyBWYXJpYWJsZSIsICczX1JlcCc6ICJEMyBSZXBsaWNhdGUiLCAnNF9WYXInOiAiRDQgVmFyaWFibGUiLCAnNF9SZXAnOiAiRDQgUmVwbGljYXRlIiwnNV9WYXInOiAiRDUgVmFyaWFibGUiLCAnNV9SZXAnOiAiRDUgUmVwbGljYXRlIiwgJzZfVmFyJzogIkQ2IFZhcmlhYmxlIiwgJzZfUmVwJzoiQTYgUmVwbGljYXRlIiwgJzdfVmFyJzoiQTcgVmFyaWFibGUiLCc3X1JlcCc6ICJENyBSZXBsaWNhdGUiLCc4X1Zhcic6ICJEOCBWYXJpYWJsZSIsICc4X1JlcCc6ICJEOCBSZXBsaWNhdGUiLCAnOV9WYXInOiAiRDkgVmFyaWFibGUiLCAnOV9SZXAnOiJBOSBSZXBsaWNhdGUiLCAnMTBfVmFyJzoiQTEwIFZhcmlhYmxlIiwnMTBfUmVwJzogIkQxMCBSZXBsaWNhdGUiLCcxMV9WYXInOiAiRDExIFZhcmlhYmxlIiwgJzExX1JlcCc6ICJEMTEgUmVwbGljYXRlIiwgJzEyX1Zhcic6ICJEMTIgVmFyaWFibGUiLCAnMTJfUmVwJzogIkQxMiBSZXBsaWNhdGUifSwKICJFIjogeycxX1Zhcic6ICJFMSBWYXJpYWJsZSIsICcxX1JlcCc6ICJFMSBSZXBsaWNhdGUiLCAnMl9WYXInOiAiRTIgVmFyaWFibGUiLCAnMl9SZXAnOiAiRTIgUmVwbGljYXRlIiwnM19WYXInOiAiRTMgVmFyaWFibGUiLCAnM19SZXAnOiAiRTMgUmVwbGljYXRlIiwgJzRfVmFyJzogIkU0IFZhcmlhYmxlIiwgJzRfUmVwJzogIkU0IFJlcGxpY2F0ZSIsJzVfVmFyJzogIkU1IFZhcmlhYmxlIiwgJzVfUmVwJzogIkU1IFJlcGxpY2F0ZSIsICc2X1Zhcic6ICJFNiBWYXJpYWJsZSIsICc2X1JlcCc6IkE2IFJlcGxpY2F0ZSIsICc3X1Zhcic6IkE3IFZhcmlhYmxlIiwnN19SZXAnOiAiRTcgUmVwbGljYXRlIiwnOF9WYXInOiAiRTggVmFyaWFibGUiLCAnOF9SZXAnOiAiRTggUmVwbGljYXRlIiwgJzlfVmFyJzogIkU5IFZhcmlhYmxlIiwgJzlfUmVwJzoiQTkgUmVwbGljYXRlIiwgJzEwX1Zhcic6IkExMCBWYXJpYWJsZSIsJzEwX1JlcCc6ICJFMTAgUmVwbGljYXRlIiwnMTFfVmFyJzogIkUxMSBWYXJpYWJsZSIsICcxMV9SZXAnOiAiRTExIFJlcGxpY2F0ZSIsICcxMl9WYXInOiAiRTEyIFZhcmlhYmxlIiwgJzEyX1JlcCc6ICJFMTIgUmVwbGljYXRlIn0sCiAiRiI6IHsnMV9WYXInOiAiRjEgVmFyaWFibGUiLCAnMV9SZXAnOiAiRjEgUmVwbGljYXRlIiwgJzJfVmFyJzogIkYyIFZhcmlhYmxlIiwgJzJfUmVwJzogIkYyIFJlcGxpY2F0ZSIsJzNfVmFyJzogIkYzIFZhcmlhYmxlIiwgJzNfUmVwJzogIkYzIFJlcGxpY2F0ZSIsICc0X1Zhcic6ICJGNCBWYXJpYWJsZSIsICc0X1JlcCc6ICJGNCBSZXBsaWNhdGUiLCc1X1Zhcic6ICJGNSBWYXJpYWJsZSIsICc1X1JlcCc6ICJGNSBSZXBsaWNhdGUiLCAnNl9WYXInOiAiRjYgVmFyaWFibGUiLCAnNl9SZXAnOiJBNiBSZXBsaWNhdGUiLCAnN19WYXInOiJBNyBWYXJpYWJsZSIsJzdfUmVwJzogIkY3IFJlcGxpY2F0ZSIsJzhfVmFyJzogIkY4IFZhcmlhYmxlIiwgJzhfUmVwJzogIkY4IFJlcGxpY2F0ZSIsICc5X1Zhcic6ICJGOSBWYXJpYWJsZSIsICc5X1JlcCc6IkE5IFJlcGxpY2F0ZSIsICcxMF9WYXInOiJBMTAgVmFyaWFibGUiLCcxMF9SZXAnOiAiRjEwIFJlcGxpY2F0ZSIsJzExX1Zhcic6ICJGMTEgVmFyaWFibGUiLCAnMTFfUmVwJzogIkYxMSBSZXBsaWNhdGUiLCAnMTJfVmFyJzogIkYxMiBWYXJpYWJsZSIsICcxMl9SZXAnOiAiRjEyIFJlcGxpY2F0ZSJ9LAogIkciOiB7JzFfVmFyJzogIkcxIFZhcmlhYmxlIiwgJzFfUmVwJzogIkcxIFJlcGxpY2F0ZSIsICcyX1Zhcic6ICJHMiBWYXJpYWJsZSIsICcyX1JlcCc6ICJHMiBSZXBsaWNhdGUiLCczX1Zhcic6ICJHMyBWYXJpYWJsZSIsICczX1JlcCc6ICJHMyBSZXBsaWNhdGUiLCAnNF9WYXInOiAiRzQgVmFyaWFibGUiLCAnNF9SZXAnOiAiRzQgUmVwbGljYXRlIiwnNV9WYXInOiAiRzUgVmFyaWFibGUiLCAnNV9SZXAnOiAiRzUgUmVwbGljYXRlIiwgJzZfVmFyJzogIkc2IFZhcmlhYmxlIiwgJzZfUmVwJzoiQTYgUmVwbGljYXRlIiwgJzdfVmFyJzoiQTcgVmFyaWFibGUiLCc3X1JlcCc6ICJHNyBSZXBsaWNhdGUiLCc4X1Zhcic6ICJHOCBWYXJpYWJsZSIsICc4X1JlcCc6ICJHOCBSZXBsaWNhdGUiLCAnOV9WYXInOiAiRzkgVmFyaWFibGUiLCAnOV9SZXAnOiJBOSBSZXBsaWNhdGUiLCAnMTBfVmFyJzoiQTEwIFZhcmlhYmxlIiwnMTBfUmVwJzogIkcxMCBSZXBsaWNhdGUiLCcxMV9WYXInOiAiRzExIFZhcmlhYmxlIiwgJzExX1JlcCc6ICJHMTEgUmVwbGljYXRlIiwgJzEyX1Zhcic6ICJHMTIgVmFyaWFibGUiLCAnMTJfUmVwJzogIkcxMiBSZXBsaWNhdGUifSwKICJIIjogeycxX1Zhcic6ICJIMSBWYXJpYWJsZSIsICcxX1JlcCc6ICJIMSBSZXBsaWNhdGUiLCAnMl9WYXInOiAiSDIgVmFyaWFibGUiLCAnMl9SZXAnOiAiSDIgUmVwbGljYXRlIiwnM19WYXInOiAiSDMgVmFyaWFibGUiLCAnM19SZXAnOiAiSDMgUmVwbGljYXRlIiwgJzRfVmFyJzogIkg0IFZhcmlhYmxlIiwgJzRfUmVwJzogIkg0IFJlcGxpY2F0ZSIsJzVfVmFyJzogIkg1IFZhcmlhYmxlIiwgJzVfUmVwJzogIkg1IFJlcGxpY2F0ZSIsICc2X1Zhcic6ICJINiBWYXJpYWJsZSIsICc2X1JlcCc6IkE2IFJlcGxpY2F0ZSIsICc3X1Zhcic6IkE3IFZhcmlhYmxlIiwnN19SZXAnOiAiSDcgUmVwbGljYXRlIiwnOF9WYXInOiAiSDggVmFyaWFibGUiLCAnOF9SZXAnOiAiSDggUmVwbGljYXRlIiwgJzlfVmFyJzogIkg5IFZhcmlhYmxlIiwgJzlfUmVwJzoiQTkgUmVwbGljYXRlIiwgJzEwX1Zhcic6IkExMCBWYXJpYWJsZSIsJzEwX1JlcCc6ICJIMTAgUmVwbGljYXRlIiwnMTFfVmFyJzogIkgxMSBWYXJpYWJsZSIsICcxMV9SZXAnOiAiSDExIFJlcGxpY2F0ZSIsICcxMl9WYXInOiAiSDEyIFZhcmlhYmxlIiwgJzEyX1JlcCc6ICJIMTIgUmVwbGljYXRlIn19CmBgYAoKYGBge3B5dGhvbn0KY2xhc3MgUGxhdGVNYXBNYWtlcihGcmFtZSk6CiAgICBkZWYgX19pbml0X18oc2VsZiwgcGFyZW50PU5vbmUpOgogICAgICAgIHNlbGYucGFyZW50ID0gcGFyZW50CiAgICAgICAgZ2xvYmFsIHRhYmxlCiAgICAgICAgRnJhbWUuX19pbml0X18oc2VsZikKICAgICAgICBzZWxmLm1haW4gPSBzZWxmLm1hc3RlcgogICAgICAgIHNlbGYubWFpbi5nZW9tZXRyeSgnMTgwMHg0MDAnKQogICAgICAgIHNlbGYubWFpbi50aXRsZSgnUGxhdGVtYXAnKQogICAgICAgIGYgPSBGcmFtZShzZWxmLm1haW4pCiAgICAgICAgZi5wYWNrKGZpbGw9Qk9USCxleHBhbmQ9MSkKICAgICAgICB0YWJsZSA9IFRhYmxlQ2FudmFzKGYsIGRhdGE9ZGF0YSwgc2hvd2tleW5hbWVzaW5oZWFkZXI9VHJ1ZSwgcm93aGVpZ2h0PTQwLCB0aGVmb250PSgiIiwxMikpCiAgICAgICAgdGFibGUucmVkcmF3CiAgICAgICAgdGFibGUudXBkYXRlKCkKICAgICAgICBidG4yID0gdGsuQnV0dG9uKHRhYmxlLCB0ZXh0PSJFeHBvcnQgYXMgQ1NWIGZvciBwbGF0ZXJlYWRyIiwgY29tbWFuZCA9IHNhdmVfcGxhdGVtYXApCiAgICAgICAgdGFibGUuY3JlYXRlX3dpbmRvdygoOTAwLDMzMCksIHdpbmRvdz1idG4yKSAKICAgICAgICB0YWJsZS5zaG93KCkKICAgICAgICByZXR1cm4KICAgIApkZWYgc2F2ZV9wbGF0ZW1hcCgpOgogICAgICAgIGZyb20gdGtpbnRlcnRhYmxlLlRhYmxlc19JTyBpbXBvcnQgVGFibGVFeHBvcnRlcgogICAgICAgIGV4cG9ydGVyID0gVGFibGVFeHBvcnRlcigpCiAgICAgICAgZXhwb3J0ZXIuRXhwb3J0VGFibGVEYXRhKHRhYmxlKQogICAgICAgIHJldHVybgoKZGVmIHNhdmVfNF9yKGZpbGVuYW1lID0gIn4vRG9jdW1lbnRzL3BsYXRlcmVhZHIvcGxhdGVyZWFkcl9weXRob24vcGxhdGVtYXBfa2V5LmNzdiIpOgogICAgICAgIHBsX2RmLnRvX2NzdihmaWxlbmFtZSkKICAgIApgYGAKCgpgYGB7cHl0aG9ufQphcHA9UGxhdGVNYXBNYWtlcigpCmFwcC5tYWlubG9vcCgpCmBgYAoKYGBge3B5dGhvbn0KYXBwLnF1aXQoKQpgYGAKCmBgYHtweXRob259CmZpbGVuYW1lPSJuZXc0LmNzdiIKcGxhdGUgPSBwZC5yZWFkX2NzdihmaWxlbmFtZSkKbj0wCnBsX2xpc3RfMSA9IFtdCnBsX2xpc3RfMiA9IFtdCgpmb3IgaSBpbiAocmFuZ2UoMTIpKToKICAgIHBsX2xpc3RfMS5hcHBlbmQoKHBsYXRlLmlsb2NbOixuOm4rMV0udmFsdWVzKSkKICAgIHBsX2xpc3RfMi5hcHBlbmQoKHBsYXRlLmlsb2NbOixuKzE6bisyXS52YWx1ZXMpKQogICAgbj1uKzIKCnBsX2RmXzEgPSBwZC5EYXRhRnJhbWUobnAuY29uY2F0ZW5hdGUocGxfbGlzdF8xKSwgY29sdW1ucyA9IFsidmFyaWFibGVzIl0pCnBsX2RmXzIgPSBwZC5EYXRhRnJhbWUobnAuY29uY2F0ZW5hdGUocGxfbGlzdF8yKSwgY29sdW1ucyA9IFsicmVwbGljYXRlcyJdKQoKcGxfZGYgPSBwZC5jb25jYXQoW3BsX2RmXzFbJ3ZhcmlhYmxlcyddLHBsX2RmXzJbJ3JlcGxpY2F0ZXMnXV0sIGF4aXM9MSwga2V5cz1bJ3ZhcmlhYmxlJywicmVwbGljYXRlIl0pCgpvPTAKcm93X2xpc3QgPSBbIkEiLCJCIiwiQyIsIkQiLCJFIiwiRiIsIkciLCJIIl0KY2VsbF9saXN0ID0gW10KZm9yIGggaW4gcmFuZ2UoMTIpOgogICAgbz1vKzEKICAgIGZvciBpIGluIHJhbmdlKDgpOgogICAgICAgIGNlbGxfbGlzdC5hcHBlbmQoKHJvd19saXN0W2ldICsgc3RyKG8pKSkKCmNlbGxfbGlzdF9kZiA9IHBkLkRhdGFGcmFtZShjZWxsX2xpc3QsIGNvbHVtbnMgPSBbImNlbGwiXSkKcGxfZGYgPSBwbF9kZi5hc3NpZ24oY2VsbD1jZWxsX2xpc3RfZGYudmFsdWVzKQoKcGxfZGYKc2F2ZV80X3IoKQpgYGAKCmBgYHtyfQojVmFyaWFibGVzIChuIGlzIHRoZSBudW1iZXIgb2YgcmVwbGljYXRlcyBvZiBlYWNoIGNvbmRpdGlvbiwgd2VsbHMpCmlucHV0PC0ifi9Eb2N1bWVudHMvbmV3X2Zvcm1hdC9wcm9ncmFtbWluZy9wbGF0ZXJlYWRyL290aGVyc193b3JrL3BlZHJvX2dyb3d0aF9jdXJ2ZXMvMjAyMTEwMDFfY2NwQV9tdXRhbnRzL2NjcEFfbXV0YW50c19HQy5jc3YiCnRpbWVwb2ludHM8LTYwCndlbGxzPC0yNyAjTmVlZCB0byBzZXQgdGhlIG51bWJlciBvZiByb3dzIGluIHRoZSByYXcgZGF0YQpgYGAKCmBgYHtyfQojaW1wb3J0IHBsYXRlbWFwIGtleSBmcm9tIHB5dGhvbiBzY3JpcHQKcGxhdGVtYXA8LSJ+L0RvY3VtZW50cy9uZXdfZm9ybWF0L3Byb2dyYW1taW5nL3BsYXRlcmVhZHIvb3RoZXJzX3dvcmsvcGVkcm9fZ3Jvd3RoX2N1cnZlcy8yMDIxMTAwMV9jY3BBX211dGFudHMvcGxhdGVtYXBfa2V5LmNzdiIKcGxhdGVtYXBfaW48LXJlYWQuY3N2KHBsYXRlbWFwKQoKYGBgCgpgYGB7cn0KI0ltcG9ydCB0aGUgZmlsZSBhcyBhIGRhdGFmcmFtZQpyYXdfZGY8LXJlYWQuY3N2KGlucHV0LCBoZWFkZXIgPSBGQUxTRSwgc3RyaW5nc0FzRmFjdG9ycz1GQUxTRSkKCiNDcmVhdGUgdmVjdG9yIG9mIHRoZSBUaW1lCnRpbWVfTGFiZWxzPC1yYXdfZGZbMzgsMjoodGltZXBvaW50cysxKV0KCiNDcmVhdGUgdmVjdG9yIG9mIHRoZSBUZW1wCnRlbXBfbGFiZWxzPC1yYXdfZGZbMzksXQoKI0NyZWF0ZSB2ZWN0b3Igb2YgdGhlIEN5Y2xlIG51bWJlcgpjeWNsZV9sYWJlbHM8LXJhd19kZlszNyxdCgojVHJpbSB0aGUgZGF0YWZyYW1lCmNsZWFuX2RmPC1yYXdfZGZbNDA6KDM5K3dlbGxzKSwxOih0aW1lcG9pbnRzKzEpXQoKI0Fzc2lnbiBjb3JyZWN0IHJvdyBuYW1lcwpyb3cubmFtZXMoY2xlYW5fZGYpPC1jbGVhbl9kZlssMV0KCiNSZW1vdmUgcm93IG5hbWVzIGNvbHVtbgpjbGVhbmVzdF9kZjwtY2xlYW5fZGZbLDE6bmNvbChjbGVhbl9kZildCgojZ2V0IHJpZCBvZiBob3JyaWJsZSBmYWN0b3JzCmNsZWFuZXN0X2RmJFYyPC1hcy5udW1lcmljKGFzLmNoYXJhY3RlcihjbGVhbmVzdF9kZiRWMikpCmNsZWFuZXN0X2RmJFY0PC1hcy5udW1lcmljKGFzLmNoYXJhY3RlcihjbGVhbmVzdF9kZiRWNCkpCmNsZWFuZXN0X2RmJFY1PC1hcy5udW1lcmljKGFzLmNoYXJhY3RlcihjbGVhbmVzdF9kZiRWNSkpCmNsZWFuZXN0X2RmJFY2PC1hcy5udW1lcmljKGFzLmNoYXJhY3RlcihjbGVhbmVzdF9kZiRWNikpCmNsZWFuZXN0X2RmJFY4PC1hcy5udW1lcmljKGFzLmNoYXJhY3RlcihjbGVhbmVzdF9kZiRWOCkpCmNsZWFuZXN0X2RmJFY5PC1hcy5udW1lcmljKGFzLmNoYXJhY3RlcihjbGVhbmVzdF9kZiRWOSkpCmBgYAoKYGBge3J9CiNmaWx0ZXIgZGF0YSB0byBpbmNsdWRlIG9ubHkgdGhvc2Ugd2hpY2ggYXBwZWFyIGluIHRoZSBrZXkKaW5wdXRfdXNlZCA8LSBsZWZ0X2pvaW4ocGxhdGVtYXBfaW4sY2xlYW5lc3RfZGYsYnkgPSBjKCJjZWxsIiA9ICJWMSIpKQppbnB1dF91c2VkIDwtIGlucHV0X3VzZWRbLDI6bmNvbChpbnB1dF91c2VkKV0KaW5wdXRfdXNlZCA8LSBmaWx0ZXIoaW5wdXRfdXNlZCwgIWdyZXBsKCJWYXJpYWJsZSIsIHZhcmlhYmxlKSkKYGBgCgpgYGB7cn0KI2ZpbHRlciBkYXRhIHRvIGluY2x1ZGUgb25seSB0aG9zZSB3aGljaCBhcHBlYXIgaW4gdGhlIGtleQppbnB1dF91c2VkIDwtIGxlZnRfam9pbihwbGF0ZW1hcF9pbixjbGVhbmVzdF9kZixieSA9IGMoImNlbGwiID0gIlYxIikpCmlucHV0X3VzZWQgPC0gaW5wdXRfdXNlZFssMjpuY29sKGlucHV0X3VzZWQpXQppbnB1dF91c2VkIDwtIGZpbHRlcihpbnB1dF91c2VkLCAhZ3JlcGwoIlZhcmlhYmxlIiwgdmFyaWFibGUpKQpgYGAKCmBgYHtyfQojbWFrZSBhIGxpc3Qgb2YgdW5pcXVlIHZhcmlhYmxlcwp2YXJpYWJsZXNfbGlzdDwtaW5wdXRfdXNlZCR2YXJpYWJsZVshZHVwbGljYXRlZChpbnB1dF91c2VkJHZhcmlhYmxlKV0KYGBgCgpgYGB7cn0KcmVwbGljYXRlcyA8LSBsaXN0KCkKI21ha2UgZGZzIGZvciB0aGUgdmFyaWFibGVzCmZvciAoaSBpbiB2YXJpYWJsZXNfbGlzdCl7CiAgYSA8LSAoZmlsdGVyKGlucHV0X3VzZWQsIGlucHV0X3VzZWQkdmFyaWFibGUgPT0gaSkpCiAgYSA8LSBhWyw0Om5jb2woYSldCiAgcmVwbGljYXRlc1tbaV1dIDwtIGEKICB9CgpgYGAKCmBgYHtyfQojU3BsaXQgcmVwbGljYXRlcyBpbnRvIHNlcGFyYXRlIGRhdGFmcmFtZXMgaGVsZCBpbiBhIGxpc3QKI25yIDwtIG5yb3coY2xlYW5lc3RfZGYpCiNyZXBsaWNhdGVzPC1zcGxpdChjbGVhbmVzdF9kZiwgcmVwKDE6Y2VpbGluZyhuci9uKSwgZWFjaD1uLCBsZW5ndGgub3V0PW5yKSkKCiNDYWxjdWxhdGUgbWVhbiBhbmQgc3RhbmRhcmQgZGV2aWF0aW9uIGZvciBlYWNoIGRhdGEgcG9pbnQKbWVhbnM8LW1hcHBseShjb2xNZWFucywgcmVwbGljYXRlcykgCmNvbFNkVGhvbWFzIDwtIGZ1bmN0aW9uKHgpc3FydChyb3dNZWFucyhuYS5ybT1UUlVFLCAodCh4KS1jb2xNZWFucyh4LCBuYS5ybT1UUlVFKSleMikqKChkaW0oeClbMV0pLyhkaW0oeClbMV0tMSkpKQpzZGV2czwtbWFwcGx5KGNvbFNkVGhvbWFzLCByZXBsaWNhdGVzKQpgYGAKCmBgYHtyfQojQXBwZW5kIHRoZXNlIHRvIHRoZSBkYXRhZnJhbWVzCmU8LTEKcmVwX2xpc3Q8LWxpc3QoKQpmb3IoeCBpbiAoMTpsZW5ndGgodmFyaWFibGVzX2xpc3QpKSl7CiAgcmVwPC1yYmluZChyZXBsaWNhdGVzW1t4XV0sICh0KG1lYW5zWyx4XSkpLCh0KHNkZXZzWyx4XSkpLCgobWVhbnNbLHhdKSsoMipzZGV2c1sseF0pKSwgKG1lYW5zWyx4XS0oMipzZGV2c1sseF0pKSkKICByZXBfbGlzdFtbZV1dPC1yZXAKICBlPC1lKzEKfQpgYGAKCmBgYHtyfQojTmFtZSB0aGUgbmV3IHJvd3MKajwtMQpmb3IoeCBpbiByZXBfbGlzdCl7CiAgcm93Lm5hbWVzKHJlcF9saXN0W1tqXV0pWyhucm93KHJlcGxpY2F0ZXNbW2pdXSkrMSldPC0ibWVhbiIKICByb3cubmFtZXMocmVwX2xpc3RbW2pdXSlbKG5yb3cocmVwbGljYXRlc1tbal1dKSsyKV08LSJzdGRldiIKICByb3cubmFtZXMocmVwX2xpc3RbW2pdXSlbKG5yb3cocmVwbGljYXRlc1tbal1dKSszKV08LSJ1cHBlcmJvdW5kIgogIHJvdy5uYW1lcyhyZXBfbGlzdFtbal1dKVsobnJvdyhyZXBsaWNhdGVzW1tqXV0pKzQpXTwtImxvd2VyYm91bmQiCiAgajwtaisxCn0KYGBgCgpgYGB7cn0KI1NwbGl0IG91dCB0aGUgdGltZSBsYWJlbHMgYW5kIG1vdmUgdG8gaG91cnMgZm9ybWF0CnRpbWVfbGFiZWxzPC1hcy5udW1lcmljKHRpbWVfTGFiZWxzKQp0aW1lcG9pbnRzX2g8LXRpbWVfbGFiZWxzLzM2MDAKdGltZXBvaW50c19oIDwtIGMoIlRpbWUgKGgpIiwgdGltZXBvaW50c19oKQpgYGAKCgpgYGB7cn0KI2dldCBtZWFucwptZWFuc19vdXQgPSBsaXN0KCkKdXBwZXJfZXJyb3IgPSBsaXN0KCkKbG93ZXJfZXJyb3IgPSBsaXN0KCkKCmZvciAoaSBpbiAxOmxlbmd0aChyZXBfbGlzdCkpewogIG1lYW5zX291dFtbaV1dPC1yZXBfbGlzdFtbaV1dWzQsXQogIHVwcGVyX2Vycm9yW1tpXV08LXJlcF9saXN0W1tpXV1bNixdCiAgbG93ZXJfZXJyb3JbW2ldXTwtcmVwX2xpc3RbW2ldXVs3LF0KfQoKbmFtZXMocmVwX2xpc3QpPC12YXJpYWJsZXNfbGlzdApuYW1lcyhtZWFuc19vdXQpPC12YXJpYWJsZXNfbGlzdApuYW1lcyh1cHBlcl9lcnJvcik8LXZhcmlhYmxlc19saXN0Cm5hbWVzKGxvd2VyX2Vycm9yKTwtdmFyaWFibGVzX2xpc3QKCm1lYW5zX2ZpbjwtYmluZF9yb3dzKG1lYW5zX291dCwgLmlkID0gInZhcmlhYmxlcyIpCnVwcGVyX2Vycm9yX2ZpbjwtYmluZF9yb3dzKHVwcGVyX2Vycm9yLCAuaWQgPSAndmFyaWFibGVzJykKbG93ZXJfZXJyb3JfZmluPC1iaW5kX3Jvd3MobG93ZXJfZXJyb3IsIC5pZCA9ICd2YXJpYWJsZXMnKQpgYGAKCmBgYHtyfQpyZXBfbGlzdFsxXQpgYGAKCmBgYHtyfQptZWFuc19maW5hbDwtcmJpbmQuZGF0YS5mcmFtZShtZWFuc19maW4sIHRpbWVwb2ludHNfaCkKbWVhbnNfZmluYWxfMjwtKG1lYW5zX2ZpbmFsWywtMV0pCnJvd25hbWVzKG1lYW5zX2ZpbmFsXzIpPC1tZWFuc19maW5hbFssMV0KbWVhbnNfZmluYWxfMjwtYXMuZGF0YS5mcmFtZSh0KG1lYW5zX2ZpbmFsXzIpKQoKCnVwcGVyX2ZpbmFsPC1yYmluZC5kYXRhLmZyYW1lKHVwcGVyX2Vycm9yX2ZpbiwgdGltZXBvaW50c19oKQp1cHBlcl9maW5hbF8yPC11cHBlcl9maW5hbFssLTFdCnJvd25hbWVzKHVwcGVyX2ZpbmFsXzIpPC11cHBlcl9maW5hbFssMV0KdXBwZXJfZmluYWxfMjwtYXMuZGF0YS5mcmFtZSh0KHVwcGVyX2ZpbmFsXzIpKQoKbG93ZXJfZmluYWw8LXJiaW5kLmRhdGEuZnJhbWUobG93ZXJfZXJyb3JfZmluLCB0aW1lcG9pbnRzX2gpCmxvd2VyX2ZpbmFsXzI8LWxvd2VyX2ZpbmFsWywtMV0Kcm93bmFtZXMobG93ZXJfZmluYWxfMik8LWxvd2VyX2ZpbmFsWywxXQpsb3dlcl9maW5hbF8yPC1hcy5kYXRhLmZyYW1lKHQobG93ZXJfZmluYWxfMikpCgpgYGAKCmBgYHtyfQojTWFrZSB0aGUgZGF0YWZyYW1lIGZvciBhIGxvbmcgZm9ybWF0CmxvbmdfZGYgPC0gZGF0YS5mcmFtZShPRDU5NT1pbnRlZ2VyKCksIFRpbWU9aW50ZWdlcigpLCBTRGV2PWludGVnZXIoKSwgU2FtcGxlPWNoYXJhY3RlcigpLCBzdHJpbmdzQXNGYWN0b3JzID0gRkFMU0UpCgojR2V0IGEgbGlzdCBvZiBuYW1lcyBmb3IgdGhlIGFzc2lnbm1lbnQgb2YgdGhlIG5hbWVzCnNhbXBsZV9uYW1lcyA8LSBjb2xuYW1lcyhtZWFuc19maW5hbF8yKQoKYGBgCgpgYGB7cn0KI21ha2UgdGhlIGxvbmcgZGF0YWZyYW1lCmZvciAoaSBpbiAoMToobGVuZ3RoKHZhcmlhYmxlc19saXN0KSkpKXsKICBuZXdfZGYgPC0gZGF0YS5mcmFtZShtZWFuc19maW5hbF8yW2ldLCBtZWFuc19maW5hbF8yJFRpbWUsIGxvd2VyX2ZpbmFsXzJbaV0sIHVwcGVyX2ZpbmFsXzJbaV0sIHZhcmlhYmxlc19saXN0W2ldKQogIGNvbG5hbWVzKG5ld19kZikgPC0gYygiT0Q1OTUiLCAiVGltZSIsICJMb3dlciIsICJVcHBlciIsICJTYW1wbGUiKQogIGxvbmdfZGYgPC0gcmJpbmQuZGF0YS5mcmFtZShsb25nX2RmLCBuZXdfZGYpCn0KYGBgCgpgYGB7cn0KI3VzZSB0aGUgc2FtcGxlIG5hbWVzIHRvIG1ha2UgZmFjdG9yIGxldmVscyAtIHVzZXIgY2FuIGNyZWF0ZSB0aGlzIGxpc3QgdG8gZ2V0IGNvbnNpc3RlbnQgY29sb3Vycy4uLgpsb25nX2RmJFNhbXBsZTwtZmFjdG9yKGxvbmdfZGYkU2FtcGxlLCBsZXZlbHMgPSB2YXJpYWJsZXNfbGlzdCkKZ3JhcGhfY29sb3VyczwtYygicmVkIiwgImJsYWNrIiwiYmx1ZSIsInBlcnUiLCJicm93bjQiLCJtYWdlbnRhIiwidHVycXVvaXNlMyIsImdyZWVuIiwia2hha2kiLCIjYmxhY2siKQpncmFwaF9saW5lX29wYWNpdHkgPC0gYygxLDEsMSwxLDEsMSwxLDEsMSkKZ3BoXzFfbGFiZWxzX2RmIDwtIGRhdGEuZnJhbWUodmFyaWFibGVzX2xpc3QpCmdwaF8xX2xhYmVsc19kZiRvcGFjaXR5IDwtIGdyYXBoX2xpbmVfb3BhY2l0eQpncGhfMV9sYWJlbHNfZGYgPC0gZ3BoXzFfbGFiZWxzX2RmWyghZ3BoXzFfbGFiZWxzX2RmJG9wYWNpdHkgPT0gMCksXQpncGhfMV9sYWJlbHNfbGlzdCA8LWdwaF8xX2xhYmVsc19kZiR2YXJpYWJsZXNfbGlzdApgYGAKCgpgYGB7cn0KbG9uZ19kZiRPRDU5NSA8LSBhcy5udW1lcmljKGxvbmdfZGYkT0Q1OTUpCmxvbmdfZGYkVGltZTwtIGFzLm51bWVyaWMobG9uZ19kZiRUaW1lKQpsb25nX2RmJExvd2VyPC0gYXMubnVtZXJpYyhsb25nX2RmJExvd2VyKQpsb25nX2RmJFVwcGVyPC0gYXMubnVtZXJpYyhsb25nX2RmJFVwcGVyKQpgYGAKCmBgYHtyfQojSW5wdXQgZmllbGRzIHRvIG1ha2UgZ3JhcGggMSBwcm9ncmFtYXRpY2FsbHkKI2dwaF90aXRsZV8xPC0iR3JhcGggMSIKI2dwaF92YXJpYWJsZXNfMTwtdmFyaWFibGVzX2xpc3QKI2dwaF9jb2xvdXJzXzE8LWdyYXBoX2NvbG91cnMKCgojSW5wdXQgdG8gbWFrZSBncmFwaCAyIHByb2dyYW1hdGljYWxseQojZ3BoX3RpdGxlXzIgPC0gIkdyYXBoIDIiCiNncGhfdmFyaWFibGVzXzIgPC0gdmFyaWFibGVzX2xpc3RbMjo0XQojZ3BoX2NvbG91cnNfMiA8LSBjKCJibGFjayIsInllbGxvdyIsInJlZCIpCgojdG90YWxfZ3BoX251bWJlciA8LSAyCgpgYGAKCgpgYGB7cn0KI3ggPSAxOih0b3RhbF9ncGhfbnVtYmVyKQojZ3BoX3RpdGxlczwtcGFzdGUwKCJncGhfdGl0bGVfIiwgeCkKI2dwaF92YXJpYWJsZXM8LXBhc3RlMCgiZ3BoX3ZhcmlhYmxlc18iLCB4KQojZ3BoX2NvbG91cnM8LXBhc3RlMCgiZ3BoX3ZhcmlhYmxlc18iLCB4KQpgYGAKCmBgYHtyfQojZm9yIChpIGluIDE6KHRvdGFsX2dwaF9udW1iZXIpKXsKIyAgZ3BoX2xpbmVzPC0gbG9uZ19kZiAlPiUgZmlsdGVyKFNhbXBsZSAlaW4lIGdwaF92YXJpYWJsZXNbW2ldXSkKIyAgcHJpbnQoZ3BoX2xpbmVzKQojfQpgYGAKCgpgYGB7cn0KI0dyYXBoIDEKZ3BoXzFfbGluZXMgPC0gbG9uZ19kZiAlPiUgZmlsdGVyKFNhbXBsZSAlaW4lIGdwaF8xX2xhYmVsc19saXN0KQpncmFwaF8xID0gZ2dwbG90KGdwaF8xX2xpbmVzLCBhZXMoeD1UaW1lLCB5PU9ENTk1LCBjb2xvdXIgPSBTYW1wbGUpKSArCiAgdGhlbWUoYXhpcy5saW5lLnggPSBlbGVtZW50X2xpbmUoY29sb3VyID0gJ2JsYWNrJywgc2l6ZSA9IDEpLCBheGlzLmxpbmUueSA9IGVsZW1lbnRfbGluZShjb2xvdXIgPSAnYmxhY2snLCBzaXplID0gMSksIGxlZ2VuZC50aXRsZSA9IGVsZW1lbnRfYmxhbmsoKSkrCiAgZ2VvbV9saW5lKHNpemUgPSAxLjUpKwogIGdlb21fcmliYm9uKGFlcyh4PVRpbWUsIHltYXg9VXBwZXIsIHltaW49TG93ZXIsIGNvbG9yID0gU2FtcGxlKSwgZmlsbCA9ICJibGFjayIsIGFscGhhID0gMC4yLCBsaW5ldHlwZSA9IDApKwogIHNjYWxlX3hfY29udGludW91cyhleHBhbmQgPSBjKDAsIDApKSsgCiAgc2NhbGVfeV9jb250aW51b3VzKGV4cGFuZCA9IGMoMCwgMCkpKwogIHNjYWxlX2NvbG9yX21hbnVhbCh2YWx1ZXMgPSBncmFwaF9jb2xvdXJzKQoKcHJpbnQoZ3JhcGhfMSkKCmBgYAoKCgpgYGB7cn0KZ2dzYXZlKCJjY3BBX211dGFudF9ncm93dGhfY3VydmVzLnN2ZyIsIHBsb3QgPSBncmFwaF8xLCB3aWR0aCA9IDEwLCBoZWlnaHQgPSA3LCBkZXZpY2UgPSAnc3ZnJykKYGBgCgoK
