# Supplementary material for: The RNA-bound proteome of MRSA reveals post-transcriptional roles for helix-turn-helix DNA-binding and Rossmann-fold proteins
Source: Nat Commun. 2022 May 24;13:2883. doi: 10.1038/s41467-022-30553-8 (PMC9130240; doi:10.1038/s41467-022-30553-8)
Supplement: Supplementary file 10 — Source Data [file 41467_2022_30553_MOESM10_ESM.zip › Source_Data/Source_Data_qPCRs_Fig_8c/qPCR_analysis_Down_regulated targets.pdf.gz!/qPCR_analysis_Down_regulated targets.pdf]

qPCR analysis script

uses the summary tables with meanCp and STDCp for each gene

In [1]:

```
library(tidyr)
library(tibble)
library(dplyr)
library(ggplot2)
library(ggpubr)
library(rstatix)
```

```
Attaching package: ‘dplyr’


The following objects are masked from ‘package:stats’:

    filter, lag


The following objects are masked from ‘package:base’:

    intersect, setdiff, setequal, union



Attaching package: ‘rstatix’


The following object is masked from ‘package:stats’:

    filter
```

In [2]:

```
#Loading the ref gene data
qpcr_data1 <- read.csv2("5S+rt.txt",sep="\t",skip=1,stringsAsFactors = F) %>%
              select(Samples,MeanCp,STD.Cp)

qpcr_data1$MeanCp <-as.numeric(qpcr_data1$MeanCp)
qpcr_data1$STD.Cp <-as.numeric(qpcr_data1$STD.Cp)
head(qpcr_data1)
```

A data.frame: 6 × 3

	Samples	MeanCp	STD.Cp
	<chr>	<dbl>	<dbl>
**1**	A1, A2, A3	2.233914	0.17373958
**2**	A4, A5, A6	2.583495	0.09058652
**3**	A7, A8, A9	2.259374	0.05850851
**4**	F1, F2, F3	2.283963	0.19431976
**5**	F4, F5, F6	2.456940	0.03570864
**6**	F7, F8, F9	2.424329	0.12783155

Loading the qPCR data for gene(s) of interest

To load your data rename the .txt files in the folowing way : "thenameofgene"_gene.txt for example groL_gene.txt

In [3]:

```
myGenes <- list.files(pattern="*gene.txt")%>% lapply(read.csv2, sep="\t",skip=1,row.
              select(Samples,MeanCp,STD.Cp)
myGenes$MeanCp <-as.numeric(as.character(myGenes$MeanCp))
myGenes$STD.Cp <-as.numeric(as.character(myGenes$STD.Cp))
```

In [4]:

```
myGenes
```

A data.frame: 18 × 3

Samples	MeanCp	STD.Cp
<chr>	<dbl>	<dbl>
C1, C2, C3	9.296843	0.07853441
C4, C5, C6	9.253096	0.30903002
C7, C8, C9	8.970651	0.17307710
F1, F2, F3	9.040420	0.19911443
F4, F5, F6	9.245045	0.07711402
F7, F8, F9	9.361763	0.15005636
B1, B2, B3	16.451415	0.21851719
B4, B5, B6	16.391934	0.09703345
B7, B8, B9	16.380817	0.11339116
D1, D2, D3	18.380895	0.19350662
D4, D5, D6	18.342939	0.30467115
D7, D8, D9	17.762462	0.80912441
A1, A2, A3	12.932170	0.06865342
A4, A5, A6	13.053856	0.03646011
A7, A8, A9	13.134278	0.04441246
D1, D2, D3	13.768144	0.49292936
D4, D5, D6	13.767007	0.41823586
D7, D8, D9	13.824625	0.16120784

In [5]:

```
#Combining the two data frames
data <- rbind(myGenes,qpcr_data1)
data
```

A data.frame: 24 × 3

Samples	MeanCp	STD.Cp
<chr>	<dbl>	<dbl>
C1, C2, C3	9.296843	0.07853441
C4, C5, C6	9.253096	0.30903002
C7, C8, C9	8.970651	0.17307710

Samples	MeanCp	STD.Cp
<chr>	<dbl>	<dbl>
F1, F2, F3	9.040420	0.19911443
F4, F5, F6	9.245045	0.07711402
F7, F8, F9	9.361763	0.15005636
B1, B2, B3	16.451415	0.21851719
B4, B5, B6	16.391934	0.09703345
B7, B8, B9	16.380817	0.11339116
D1, D2, D3	18.380895	0.19350662
D4, D5, D6	18.342939	0.30467115
D7, D8, D9	17.762462	0.80912441
A1, A2, A3	12.932170	0.06865342
A4, A5, A6	13.053856	0.03646011
A7, A8, A9	13.134278	0.04441246
D1, D2, D3	13.768144	0.49292936
D4, D5, D6	13.767007	0.41823586
D7, D8, D9	13.824625	0.16120784
A1, A2, A3	2.233914	0.17373958
A4, A5, A6	2.583495	0.09058652
A7, A8, A9	2.259374	0.05850851
F1, F2, F3	2.283963	0.19431976
F4, F5, F6	2.456940	0.03570864
F7, F8, F9	2.424329	0.12783155

In [6]:

```
samples_wells = select(data,Samples)%>% as.list()
```

Here you have to provide the names of your samples

it is assumed that samples were in the same order for each gene

In [7]:

```
SampleName=c("WT_1","WT_2","WT_3","DccpA_1","DccpA_2","DccpA_3")
```

Below you will have to do changes to Sample_name and Primers

In [8]:

```
#Check the order of the genes as the data is uploaded by checking the values
#Creating df for the primers used (HK-housekeeping gene,test-target gene)
```

```
primer_key <- data.frame(Samples = samples_wells,
                         Sample_name = c(rep(SampleName,4)),  #change this number 3
                         Primers = c(rep("sprD",6),rep("srtA",6),rep("fmtC",6),rep("
primer_key
```

A data.frame: 24 × 3

Samples	Sample_name	Primers
<fct>	<fct>	<fct>
C1, C2, C3	WT_1	sprD
C4, C5, C6	WT_2	sprD
C7, C8, C9	WT_3	sprD
F1, F2, F3	DccpA_1	sprD
F4, F5, F6	DccpA_2	sprD
F7, F8, F9	DccpA_3	sprD
B1, B2, B3	WT_1	srtA
B4, B5, B6	WT_2	srtA
B7, B8, B9	WT_3	srtA
D1, D2, D3	DccpA_1	srtA
D4, D5, D6	DccpA_2	srtA
D7, D8, D9	DccpA_3	srtA
A1, A2, A3	WT_1	fmtC
A4, A5, A6	WT_2	fmtC
A7, A8, A9	WT_3	fmtC
D1, D2, D3	DccpA_1	fmtC
D4, D5, D6	DccpA_2	fmtC
D7, D8, D9	DccpA_3	fmtC
A1, A2, A3	WT_1	5S
A4, A5, A6	WT_2	5S
A7, A8, A9	WT_3	5S
F1, F2, F3	DccpA_1	5S
F4, F5, F6	DccpA_2	5S
F7, F8, F9	DccpA_3	5S

In [9]:

```
#Combining the 2 df qpct_data and primer_key
### Because the data contains several separate plate runs different genes were havin
### So join the data with the primer_key by index!
tidy_data1 <-
left_join(rownames_to_column(primer_key), rownames_to_column(data), by="rowname") %>
  separate(Sample_name, into = c("Sample", "replicate"), sep = "_")
tidy_data1
```

A data.frame: 24 × 8

rowname	Samples.x	Sample	replicate	Primers	Samples.y	MeanCp	STD.Cp

rowname	Samples.x	Sample	replicate	Primers	Samples.y	MeanCp	STD.Cp
<chr>	<fct>	<chr>	<chr>	<fct>	<chr>	<dbl>	<dbl>
1	C1, C2, C3	WT	1	sprD	C1, C2, C3	9.296843	0.07853441
2	C4, C5, C6	WT	2	sprD	C4, C5, C6	9.253096	0.30903002
3	C7, C8, C9	WT	3	sprD	C7, C8, C9	8.970651	0.17307710
4	F1, F2, F3	DccpA	1	sprD	F1, F2, F3	9.040420	0.19911443
5	F4, F5, F6	DccpA	2	sprD	F4, F5, F6	9.245045	0.07711402
6	F7, F8, F9	DccpA	3	sprD	F7, F8, F9	9.361763	0.15005636
7	B1, B2, B3	WT	1	srtA	B1, B2, B3	16.451415	0.21851719
8	B4, B5, B6	WT	2	srtA	B4, B5, B6	16.391934	0.09703345
9	B7, B8, B9	WT	3	srtA	B7, B8, B9	16.380817	0.11339116
10	D1, D2, D3	DccpA	1	srtA	D1, D2, D3	18.380895	0.19350662
11	D4, D5, D6	DccpA	2	srtA	D4, D5, D6	18.342939	0.30467115
12	D7, D8, D9	DccpA	3	srtA	D7, D8, D9	17.762462	0.80912441
13	A1, A2, A3	WT	1	fmtC	A1, A2, A3	12.932170	0.06865342
14	A4, A5, A6	WT	2	fmtC	A4, A5, A6	13.053856	0.03646011
15	A7, A8, A9	WT	3	fmtC	A7, A8, A9	13.134278	0.04441246
16	D1, D2, D3	DccpA	1	fmtC	D1, D2, D3	13.768144	0.49292936
17	D4, D5, D6	DccpA	2	fmtC	D4, D5, D6	13.767007	0.41823586
18	D7, D8, D9	DccpA	3	fmtC	D7, D8, D9	13.824625	0.16120784
19	A1, A2, A3	WT	1	5S	A1, A2, A3	2.233914	0.17373958
20	A4, A5, A6	WT	2	5S	A4, A5, A6	2.583495	0.09058652
21	A7, A8, A9	WT	3	5S	A7, A8, A9	2.259374	0.05850851
22	F1, F2, F3	DccpA	1	5S	F1, F2, F3	2.283963	0.19431976
23	F4, F5, F6	DccpA	2	5S	F4, F5, F6	2.456940	0.03570864
24	F7, F8, F9	DccpA	3	5S	F7, F8, F9	2.424329	0.12783155

In [10]:

```
#Creating df of list for each gene
#Extracting the ref gene - change if you have used different ref gene from 5S
dfList <- split(tidy_data1,tidy_data1$Primers)
ref_gene <- dfList[["5S"]]
ref_gene
```

A data.frame: 6 × 8

	rowname	Samples.x	Sample	replicate	Primers	Samples.y	MeanCp	STD.Cp
	<chr>	<fct>	<chr>	<chr>	<fct>	<chr>	<dbl>	<dbl>
**19**	19	A1, A2, A3	WT	1	5S	A1, A2, A3	2.233914	0.17373958
**20**	20	A4, A5, A6	WT	2	5S	A4, A5, A6	2.583495	0.09058652
**21**	21	A7, A8, A9	WT	3	5S	A7, A8, A9	2.259374	0.05850851

	rowname	Samples.x	Sample	replicate	Primers	Samples.y	MeanCp	STD.Cp
	<chr>	<fct>	<chr>	<chr>	<fct>	<chr>	<dbl>	<dbl>
**22**	22	F1, F2, F3	DccpA	1	5S	F1, F2, F3	2.283963	0.19431976
**23**	23	F4, F5, F6	DccpA	2	5S	F4, F5, F6	2.456940	0.03570864
**24**	24	F7, F8, F9	DccpA	3	5S	F7, F8, F9	2.424329	0.12783155

Normalize by the ref_gene, calculate dCp,ddCp,realConcentration values

In [11]:

```
dfList[["5S"]] <- NULL
newdfList <- list()
  for (i in 1:length(dfList))
    {
    dCp <- left_join(dfList[[i]], ref_gene, by = c("Sample", "replicate")) %>%
      #Calculate deltaCp values (target - ref gene)
      mutate(delta_Cp = MeanCp.x - MeanCp.y)%>%
      #Calculate the mean deltaCp for each sample
      group_by(Sample) %>%
      mutate(mean_delta_Cp = mean(delta_Cp),
             sd_delta_Cp = sd(delta_Cp))
    #Calculate the delta delta Cp of each replicate compared to the mean of the cont
    mean_control <- filter(dCp, Sample == "WT") %>% pull(mean_delta_Cp)
    ddCp<- dCp %>%
      mutate(delta_delta_Cp = delta_Cp - mean_control) %>%
      #Calculating relative DNA concentration
      mutate(rel_conc = 2^-delta_delta_Cp) %>%
      group_by(Sample) %>%
      mutate(mean_rel_conc = mean(rel_conc))%>%
      mutate(sd_rel_conc=sd(rel_conc))
      newdfList <- append(newdfList,list(ddCp),0)
  }
final_df<-bind_rows(newdfList, .id = "column_label")
#Ordering the genes/primers
final_df$Primers.x <- factor(final_df$Primers.x, levels = c("sprD","srtA","fmtC"))
#Ordering the samples
final_df$Sample <- factor(final_df$Sample, levels = c("WT","DccpA"))
final_df
```

column_label	rowname.x	Samples.x.x	Sample	replicate	Primers.x	Samples.y.x	MeanCp.x	STD.
<chr>	<chr>	<fct>	<fct>	<chr>	<fct>	<chr>	<dbl>	<
1	7	B1, B2, B3	WT	1	srtA	B1, B2, B3	16.451415	0.2185
1	8	B4, B5, B6	WT	2	srtA	B4, B5, B6	16.391934	0.0970
1	9	B7, B8, B9	WT	3	srtA	B7, B8, B9	16.380817	0.1133
1	10	D1, D2, D3	DccpA	1	srtA	D1, D2, D3	18.380895	0.1935
1	11	D4, D5, D6	DccpA	2	srtA	D4, D5, D6	18.342939	0.3046
1	12	D7, D8, D9	DccpA	3	srtA	D7, D8, D9	17.762462	0.8091
2	1	C1, C2, C3	WT	1	sprD	C1, C2, C3	9.296843	0.0785
2	2	C4, C5, C6	WT	2	sprD	C4, C5, C6	9.253096	0.3090
2	3	C7, C8, C9	WT	3	sprD	C7, C8, C9	8.970651	0.1730

column_label	rowname.x	Samples.x.x	Sample	replicate	Primers.x	Samples.y.x	MeanCp.x	STD.
<chr>	<chr>	<fct>	<fct>	<chr>	<fct>	<chr>	<dbl>	<(
2	4	F1, F2, F3	DccpA	1	sprD	F1, F2, F3	9.040420	0.1991
2	5	F4, F5, F6	DccpA	2	sprD	F4, F5, F6	9.245045	0.0771
2	6	F7, F8, F9	DccpA	3	sprD	F7, F8, F9	9.361763	0.1500
3	13	A1, A2, A3	WT	1	fmtC	A1, A2, A3	12.932170	0.0686
3	14	A4, A5, A6	WT	2	fmtC	A4, A5, A6	13.053856	0.0364
3	15	A7, A8, A9	WT	3	fmtC	A7, A8, A9	13.134278	0.0444
3	16	D1, D2, D3	DccpA	1	fmtC	D1, D2, D3	13.768144	0.4929
3	17	D4, D5, D6	DccpA	2	fmtC	D4, D5, D6	13.767007	0.4182
3	18	D7, D8, D9	DccpA	3	fmtC	D7, D8, D9	13.824625	0.1612

In [12]:

```
#Statistics
  stat.test <- final_df %>% group_by(Primers.x)%>%
  t_test(rel_conc~Sample,p.adjust.method = "holm",detailed=T)%>%
  add_significance()
#Change name of the statistics file
  stat.test
  write.csv(stat.test,file = "statfile.txt")


  #stat.test <-stat.test %>% add_y_position()
  stat.test <-stat.test %>% add_xy_position(x="Primers.x")
```

A rstatix_test: 3 × 17

	Primers.x	estimate	estimate1	estimate2	.y.	group1	group2	n1	n2	statistic
	<fct>	<dbl>	<dbl>	<dbl>	<chr>	<chr>	<chr>	<int>	<int>	<dbl>
**1**	sprD	0.01460137	1.007284	0.9926822	rel_conc	WT	DccpA	3	3	0.1598342
**2**	srtA	0.69716647	1.007504	0.3103377	rel_conc	WT	DccpA	3	3	6.8191730
**3**	fmtC	0.39753962	1.006637	0.6090975	rel_conc	WT	DccpA	3	3	4.6818614

If you do not want to have the WT in the final figure run the cell below (optional)

In [13]:

```
#To run this cell Remove the #
  #final_df <- filter(final_df,Sample != "WT")
```

To plot

In [13]:

```
#To have the signif label as numerical use label = "p.adj" or "p"
#To have is as * or ns use label="p.adj.signif" or "p.signif"

  p<- ggbarplot(
    final_df, x = "Primers.x", y = "rel_conc", fill = "Sample",stat="identity",
```

```
    add = c("mean_sd","point"), palette = "Blues",position = position_dodge(0.8)) +
    stat_pvalue_manual(stat.test, label= "p.signif",tip.length = 0.01,hide.ns = F)+
    scale_y_continuous(expand = expansion(mult=c(0.01,0.1)),name = "Expression relat
    theme(legend.position = 'right',axis.title.x=element_blank())


p
```

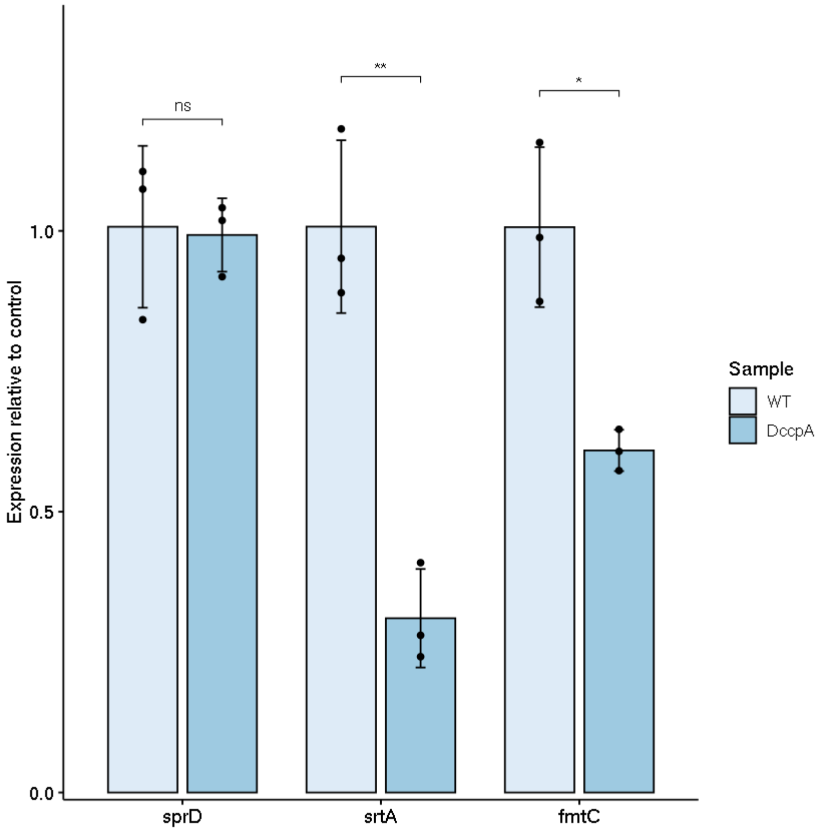

In [14]:

```
#Saving the graph change the name of the file
##You can also change the width and height to your needs
ggsave(p,filename = "Down_regulated_targets.pdf",width = 110, height = 140, units =
```

In [ ]:
