# Supplementary material for: The RNA-bound proteome of MRSA reveals post-transcriptional roles for helix-turn-helix DNA-binding and Rossmann-fold proteins
Source: Nat Commun. 2022 May 24;13:2883. doi: 10.1038/s41467-022-30553-8 (PMC9130240; doi:10.1038/s41467-022-30553-8)
Supplement: Supplementary file 10 — Source Data [file 41467_2022_30553_MOESM10_ESM.zip › Source_Data/Source_Data_qPCRs_Fig_8c/Down_regulated_targets.pdf.gz!/Down_regulated_targets.pdf]

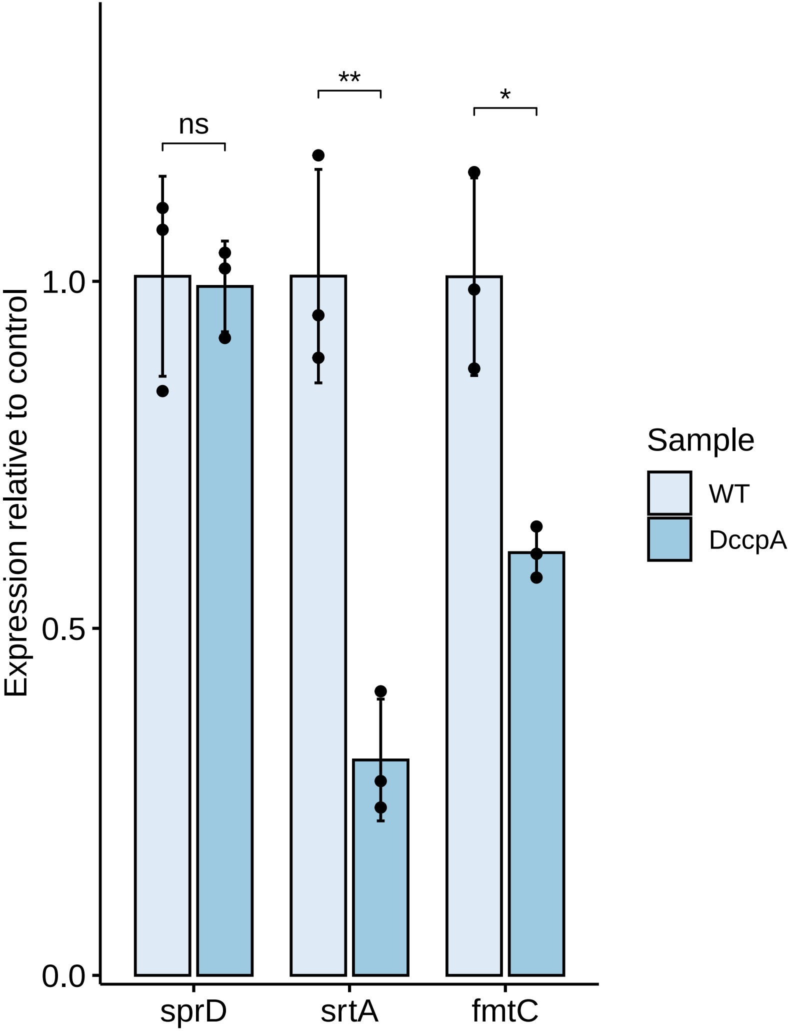
ns
**
*
1.0
0.5
0.0
Expression relative to control
sprD
srtA
fmtC
Sample
WT
DccpA
