# Supplementary material for: The RNA-bound proteome of MRSA reveals post-transcriptional roles for helix-turn-helix DNA-binding and Rossmann-fold proteins
Source: Nat Commun. 2022 May 24;13:2883. doi: 10.1038/s41467-022-30553-8 (PMC9130240; doi:10.1038/s41467-022-30553-8)
Supplement: Supplementary file 10 — Source Data [file 41467_2022_30553_MOESM10_ESM.zip › Source_Data/Source_Data_qPCRs_Fig_8c/211125_upregulated genes.pdf.gz!/211125_upregulated genes.pdf]

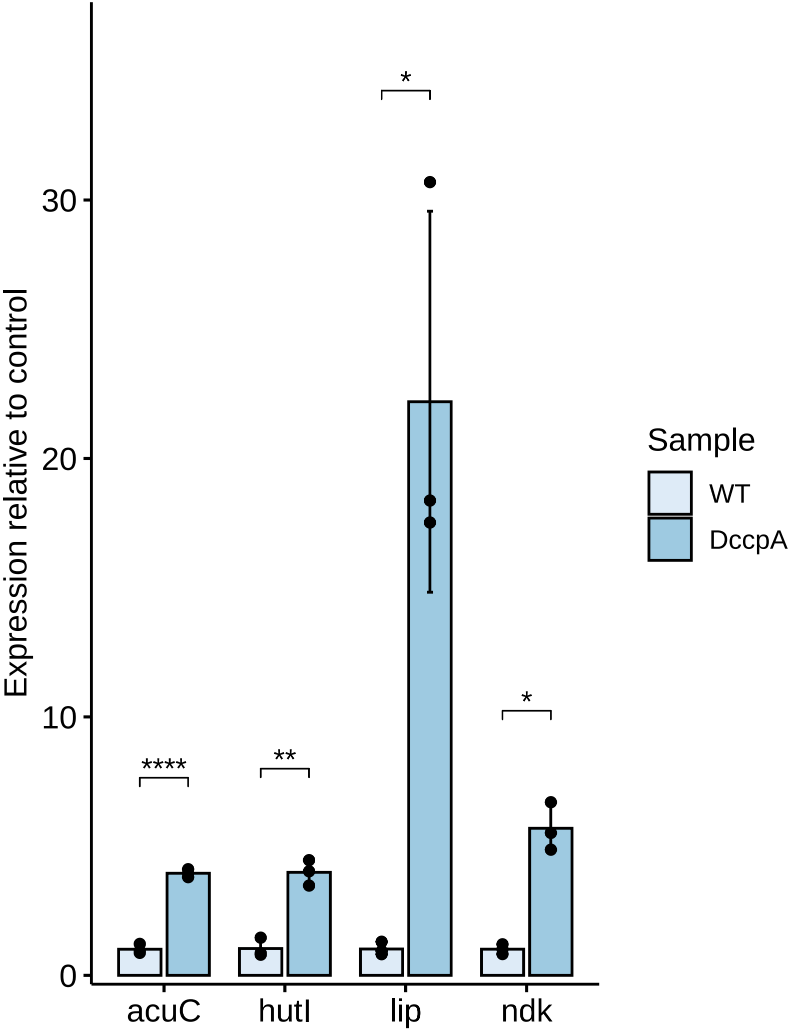

Expression relative to control
0
10
20
30
****
**
*
*
acuC
hutI
lip
ndk
Sample
WT
DccpA
