# Supplementary material for: The RNA-bound proteome of MRSA reveals post-transcriptional roles for helix-turn-helix DNA-binding and Rossmann-fold proteins
Source: Nat Commun. 2022 May 24;13:2883. doi: 10.1038/s41467-022-30553-8 (PMC9130240; doi:10.1038/s41467-022-30553-8)
Supplement: Supplementary file 10 — Source Data [file 41467_2022_30553_MOESM10_ESM.zip › Source_Data/Source_Data_qPCRs_Fig_8c/qPCR_analysis_Upregulated targets_updated.pdf.gz!/qPCR_analysis_Upregulated targets_updated.pdf]

qPCR analysis script

uses the summary tables with meanCp and STDCp for each gene

In [1]:
```
library(tidyr)
library(tibble)
library(dplyr)
library(ggplot2)
library(ggpubr)
library(rstatix)
```

```
Attaching package: ‘dplyr’


The following objects are masked from ‘package:stats’:

    filter, lag


The following objects are masked from ‘package:base’:

    intersect, setdiff, setequal, union



Attaching package: ‘rstatix’


The following object is masked from ‘package:stats’:

    filter
```

In [4]:
```
#Loading the ref gene data
qpcr_data1 <- read.csv2("5S+rt.txt",sep="\t",skip=1,stringsAsFactors = F) %>%
              select(Samples,MeanCp,STD.Cp)

qpcr_data1$MeanCp <-as.numeric(qpcr_data1$MeanCp)
qpcr_data1$STD.Cp <-as.numeric(qpcr_data1$STD.Cp)
head(qpcr_data1)
```

A data.frame: 6 × 3

	Samples	MeanCp	STD.Cp
	<chr>	<dbl>	<dbl>
**1**	A1, A2, A3	2.233914	0.17373958
**2**	A4, A5, A6	2.583495	0.09058652
**3**	A7, A8, A9	2.259374	0.05850851
**4**	F1, F2, F3	2.283963	0.19431976
**5**	F4, F5, F6	2.456940	0.03570864
**6**	F7, F8, F9	2.424329	0.12783155

Loading the qPCR data for gene(s) of interest

To load your data rename the .txt files in the folowing way : "thenameofgene"_gene.txt for example groL_gene.txt

In [5]:

```
myGenes <- list.files(pattern="*gene.txt")%>% lapply(read.csv2, sep="\t",skip=1,row.
              select(Samples,MeanCp,STD.Cp)
myGenes$MeanCp <-as.numeric(as.character(myGenes$MeanCp))
myGenes$STD.Cp <-as.numeric(as.character(myGenes$STD.Cp))
```

In [6]:

```
myGenes
```

A data.frame: 24 × 3

Samples	MeanCp	STD.Cp
<chr>	<dbl>	<dbl>
B1, B2, B3	13.80227	0.13747312
B4, B5, B6	13.78620	0.10934731
B7, B8, B9	13.94584	0.08479463
E1, E2, E3	11.78994	0.13688435
E4, E5, E6	11.90482	0.05462771
E7, E8, E9	11.98442	0.45562796
C1, C2, C3	18.37639	0.25200225
C4, C5, C6	17.85605	0.43368671
C7, C8, C9	18.29315	0.16333544
G1, G2, G3	16.09070	0.46936915
G4, G5, G6	16.47694	0.26480024
G7, G8, G9	16.08444	0.21193850
A1, A2, A3	17.62809	0.28378126
A4, A5, A6	17.30971	0.05638497
A7, A8, A9	17.45422	0.15194571
E1, E2, E3	13.25800	0.09825928
E4, E5, E6	13.36259	0.06356886
E7, E8, E9	12.58958	0.24585197
B1, B2, B3	13.29259	0.18490035
B4, B5, B6	13.09857	0.07051074
B7, B8, B9	13.02434	0.06982479
F1, F2, F3	10.60116	0.17039556
F4, F5, F6	10.95488	0.12423324
F7, F8, F9	10.46004	0.05693210

In [7]:

```
#Combining the two data frames
data <- rbind(myGenes,qpcr_data1)
data
```

A data.frame: 30 × 3

Samples	MeanCp	STD.Cp
<chr>	<dbl>	<dbl>
B1, B2, B3	13.802266	0.13747312
B4, B5, B6	13.786204	0.10934731
B7, B8, B9	13.945836	0.08479463
E1, E2, E3	11.789943	0.13688435
E4, E5, E6	11.904817	0.05462771
E7, E8, E9	11.984417	0.45562796
C1, C2, C3	18.376394	0.25200225
C4, C5, C6	17.856050	0.43368671
C7, C8, C9	18.293152	0.16333544
G1, G2, G3	16.090696	0.46936915
G4, G5, G6	16.476944	0.26480024
G7, G8, G9	16.084436	0.21193850
A1, A2, A3	17.628088	0.28378126
A4, A5, A6	17.309712	0.05638497
A7, A8, A9	17.454222	0.15194571
E1, E2, E3	13.257998	0.09825928
E4, E5, E6	13.362589	0.06356886
E7, E8, E9	12.589583	0.24585197
B1, B2, B3	13.292589	0.18490035
B4, B5, B6	13.098574	0.07051074
B7, B8, B9	13.024338	0.06982479
F1, F2, F3	10.601164	0.17039556
F4, F5, F6	10.954879	0.12423324
F7, F8, F9	10.460045	0.05693210
A1, A2, A3	2.233914	0.17373958
A4, A5, A6	2.583495	0.09058652
A7, A8, A9	2.259374	0.05850851
F1, F2, F3	2.283963	0.19431976
F4, F5, F6	2.456940	0.03570864
F7, F8, F9	2.424329	0.12783155

In [8]:

```
samples_wells = select(data,Samples)%>% as.list()
```

Here you have to provide the names of your samples

it is assumed that samples were in the same order for each gene

In [9]:

```
SampleName=c("WT_1","WT_2","WT_3","DccpA_1","DccpA_2","DccpA_3")
```

Below you will have to do changes to Sample_name and Primers

In [10]:

```
#Check the order of the genes as the data is uploaded by checking the values
#Creating df for the primers used (HK-housekeeping gene,test-target gene)

primer_key <- data.frame(Samples = samples_wells,
                         Sample_name = c(rep(SampleName,5)),  #change this number 3
                         Primers = c(rep("acuC",6),rep("hutI",6),rep("lip",6),rep("n
primer_key
```

A data.frame: 30 × 3

Samples	Sample_name	Primers
<fct>	<fct>	<fct>
B1, B2, B3	WT_1	acuC
B4, B5, B6	WT_2	acuC
B7, B8, B9	WT_3	acuC
E1, E2, E3	DccpA_1	acuC
E4, E5, E6	DccpA_2	acuC
E7, E8, E9	DccpA_3	acuC
C1, C2, C3	WT_1	hutI
C4, C5, C6	WT_2	hutI
C7, C8, C9	WT_3	hutI
G1, G2, G3	DccpA_1	hutI
G4, G5, G6	DccpA_2	hutI
G7, G8, G9	DccpA_3	hutI
A1, A2, A3	WT_1	lip
A4, A5, A6	WT_2	lip
A7, A8, A9	WT_3	lip
E1, E2, E3	DccpA_1	lip
E4, E5, E6	DccpA_2	lip
E7, E8, E9	DccpA_3	lip
B1, B2, B3	WT_1	ndk
B4, B5, B6	WT_2	ndk
B7, B8, B9	WT_3	ndk
F1, F2, F3	DccpA_1	ndk

Samples	Sample_name	Primers
<fct>	<fct>	<fct>
F4, F5, F6	DccpA_2	ndk
F7, F8, F9	DccpA_3	ndk
A1, A2, A3	WT_1	5S
A4, A5, A6	WT_2	5S
A7, A8, A9	WT_3	5S
F1, F2, F3	DccpA_1	5S
F4, F5, F6	DccpA_2	5S
F7, F8, F9	DccpA_3	5S

In [11]:

```
#Combining the 2 df qpct_data and primer_key
### Because the data contains several separate plate runs different genes were havin
### So join the data with the primer_key by index!
tidy_data1 <-
left_join(rownames_to_column(primer_key), rownames_to_column(data), by="rowname") %>
  separate(Sample_name, into = c("Sample", "replicate"), sep = "_")
tidy_data1
```

A data.frame: 30 × 8

rowname	Samples.x	Sample	replicate	Primers	Samples.y	MeanCp	STD.Cp
<chr>	<fct>	<chr>	<chr>	<fct>	<chr>	<dbl>	<dbl>
1	B1, B2, B3	WT	1	acuC	B1, B2, B3	13.802266	0.13747312
2	B4, B5, B6	WT	2	acuC	B4, B5, B6	13.786204	0.10934731
3	B7, B8, B9	WT	3	acuC	B7, B8, B9	13.945836	0.08479463
4	E1, E2, E3	DccpA	1	acuC	E1, E2, E3	11.789943	0.13688435
5	E4, E5, E6	DccpA	2	acuC	E4, E5, E6	11.904817	0.05462771
6	E7, E8, E9	DccpA	3	acuC	E7, E8, E9	11.984417	0.45562796
7	C1, C2, C3	WT	1	hutI	C1, C2, C3	18.376394	0.25200225
8	C4, C5, C6	WT	2	hutI	C4, C5, C6	17.856050	0.43368671
9	C7, C8, C9	WT	3	hutI	C7, C8, C9	18.293152	0.16333544
10	G1, G2, G3	DccpA	1	hutI	G1, G2, G3	16.090696	0.46936915
11	G4, G5, G6	DccpA	2	hutI	G4, G5, G6	16.476944	0.26480024
12	G7, G8, G9	DccpA	3	hutI	G7, G8, G9	16.084436	0.21193850
13	A1, A2, A3	WT	1	lip	A1, A2, A3	17.628088	0.28378126
14	A4, A5, A6	WT	2	lip	A4, A5, A6	17.309712	0.05638497
15	A7, A8, A9	WT	3	lip	A7, A8, A9	17.454222	0.15194571
16	E1, E2, E3	DccpA	1	lip	E1, E2, E3	13.257998	0.09825928
17	E4, E5, E6	DccpA	2	lip	E4, E5, E6	13.362589	0.06356886
18	E7, E8, E9	DccpA	3	lip	E7, E8, E9	12.589583	0.24585197

rowname	Samples.x	Sample	replicate	Primers	Samples.y	MeanCp	STD.Cp
<chr>	<fct>	<chr>	<chr>	<fct>	<chr>	<dbl>	<dbl>
19	B1, B2, B3	WT	1	ndk	B1, B2, B3	13.292589	0.18490035
20	B4, B5, B6	WT	2	ndk	B4, B5, B6	13.098574	0.07051074
21	B7, B8, B9	WT	3	ndk	B7, B8, B9	13.024338	0.06982479
22	F1, F2, F3	DccpA	1	ndk	F1, F2, F3	10.601164	0.17039556
23	F4, F5, F6	DccpA	2	ndk	F4, F5, F6	10.954879	0.12423324
24	F7, F8, F9	DccpA	3	ndk	F7, F8, F9	10.460045	0.05693210
25	A1, A2, A3	WT	1	5S	A1, A2, A3	2.233914	0.17373958
26	A4, A5, A6	WT	2	5S	A4, A5, A6	2.583495	0.09058652
27	A7, A8, A9	WT	3	5S	A7, A8, A9	2.259374	0.05850851
28	F1, F2, F3	DccpA	1	5S	F1, F2, F3	2.283963	0.19431976
29	F4, F5, F6	DccpA	2	5S	F4, F5, F6	2.456940	0.03570864
30	F7, F8, F9	DccpA	3	5S	F7, F8, F9	2.424329	0.12783155

In [12]:

```
#Creating df of list for each gene
#Extracting the ref gene - change if you have used different ref gene from 5S
dfList <- split(tidy_data1,tidy_data1$Primers)
ref_gene <- dfList[["5S"]]
ref_gene
```

A data.frame: 6 × 8

	rowname	Samples.x	Sample	replicate	Primers	Samples.y	MeanCp	STD.Cp
	<chr>	<fct>	<chr>	<chr>	<fct>	<chr>	<dbl>	<dbl>
**25**	25	A1, A2, A3	WT	1	5S	A1, A2, A3	2.233914	0.17373958
**26**	26	A4, A5, A6	WT	2	5S	A4, A5, A6	2.583495	0.09058652
**27**	27	A7, A8, A9	WT	3	5S	A7, A8, A9	2.259374	0.05850851
**28**	28	F1, F2, F3	DccpA	1	5S	F1, F2, F3	2.283963	0.19431976
**29**	29	F4, F5, F6	DccpA	2	5S	F4, F5, F6	2.456940	0.03570864
**30**	30	F7, F8, F9	DccpA	3	5S	F7, F8, F9	2.424329	0.12783155

Normalize by the ref_gene, calculate dCp,ddCp,realConcentration values

In [13]:

```
dfList[["5S"]] <- NULL
newdfList <- list()
  for (i in 1:length(dfList))
    {
    dCp <- left_join(dfList[[i]], ref_gene, by = c("Sample", "replicate")) %>%
      #Calculate deltaCp values (target - ref gene)
      mutate(delta_Cp = MeanCp.x - MeanCp.y)%>%
      #Calculate the mean deltaCp for each sample
      group_by(Sample) %>%
      mutate(mean_delta_Cp = mean(delta_Cp),
```

```
             sd_delta_Cp = sd(delta_Cp))
    #Calculate the delta delta Cp of each replicate compared to the mean of the cont
    mean_control <- filter(dCp, Sample == "WT") %>% pull(mean_delta_Cp)
    ddCp<- dCp %>%
      mutate(delta_delta_Cp = delta_Cp - mean_control) %>%
      #Calculating relative DNA concentration
      mutate(rel_conc = 2^-delta_delta_Cp) %>%
      group_by(Sample) %>%
      mutate(mean_rel_conc = mean(rel_conc))%>%
      mutate(sd_rel_conc=sd(rel_conc))
      newdfList <- append(newdfList,list(ddCp),0)
  }
final_df<-bind_rows(newdfList, .id = "column_label")
#Ordering the genes/primers
final_df$Primers.x <- factor(final_df$Primers.x, levels = c("acuC","hutI", "lip","nd
#Ordering the samples
final_df$Sample <- factor(final_df$Sample, levels = c("WT","DccpA"))
final_df
```

column_label	rowname.x	Samples.x.x	Sample	replicate	Primers.x	Samples.y.x	MeanCp.x	STD.
<chr>	<chr>	<fct>	<fct>	<chr>	<fct>	<chr>	<dbl>	<
1	19	B1, B2, B3	WT	1	ndk	B1, B2, B3	13.29259	0.1849
1	20	B4, B5, B6	WT	2	ndk	B4, B5, B6	13.09857	0.0705
1	21	B7, B8, B9	WT	3	ndk	B7, B8, B9	13.02434	0.0698
1	22	F1, F2, F3	DccpA	1	ndk	F1, F2, F3	10.60116	0.1703
1	23	F4, F5, F6	DccpA	2	ndk	F4, F5, F6	10.95488	0.1242
1	24	F7, F8, F9	DccpA	3	ndk	F7, F8, F9	10.46004	0.0569
2	13	A1, A2, A3	WT	1	lip	A1, A2, A3	17.62809	0.2837
2	14	A4, A5, A6	WT	2	lip	A4, A5, A6	17.30971	0.0563
2	15	A7, A8, A9	WT	3	lip	A7, A8, A9	17.45422	0.1519
2	16	E1, E2, E3	DccpA	1	lip	E1, E2, E3	13.25800	0.0982
2	17	E4, E5, E6	DccpA	2	lip	E4, E5, E6	13.36259	0.0635
2	18	E7, E8, E9	DccpA	3	lip	E7, E8, E9	12.58958	0.2458
3	7	C1, C2, C3	WT	1	hutI	C1, C2, C3	18.37639	0.2520
3	8	C4, C5, C6	WT	2	hutI	C4, C5, C6	17.85605	0.4336
3	9	C7, C8, C9	WT	3	hutI	C7, C8, C9	18.29315	0.1633
3	10	G1, G2, G3	DccpA	1	hutI	G1, G2, G3	16.09070	0.4693
3	11	G4, G5, G6	DccpA	2	hutI	G4, G5, G6	16.47694	0.2648
3	12	G7, G8, G9	DccpA	3	hutI	G7, G8, G9	16.08444	0.2119
4	1	B1, B2, B3	WT	1	acuC	B1, B2, B3	13.80227	0.1374
4	2	B4, B5, B6	WT	2	acuC	B4, B5, B6	13.78620	0.1093
4	3	B7, B8, B9	WT	3	acuC	B7, B8, B9	13.94584	0.0847
4	4	E1, E2, E3	DccpA	1	acuC	E1, E2, E3	11.78994	0.1368
4	5	E4, E5, E6	DccpA	2	acuC	E4, E5, E6	11.90482	0.0546

column_label	rowname.x	Samples.x.x	Sample	replicate	Primers.x	Samples.y.x	MeanCp.x	STD.
<chr>	<chr>	<fct>	<fct>	<chr>	<fct>	<chr>	<dbl>	<(
4	6	E7, E8, E9	DccpA	3	acuC	E7, E8, E9	11.98442	0.4556

In [14]:

```
#Statistics
  stat.test <- final_df %>% group_by(Primers.x)%>%
  t_test(rel_conc~Sample,p.adjust.method = "holm",detailed=T)%>%
  add_significance()
#Change name of the statistics file
  stat.test
  write.csv(stat.test,file = "statfile.txt")


  #stat.test <-stat.test %>% add_y_position()
  stat.test <-stat.test %>% add_xy_position(x="Primers.x")
```

A rstatix_test: 4 × 17

	Primers.x	estimate	estimate1	estimate2	.y.	group1	group2	n1	n2	statistic
	<fct>	<dbl>	<dbl>	<dbl>	<chr>	<chr>	<chr>	<int>	<int>	<dbl>
**1**	acuC	-2.939713	1.010474	3.950187	rel_conc	WT	DccpA	3	3	-21.336449
**2**	hutI	-2.947205	1.038467	3.985672	rel_conc	WT	DccpA	3	3	-8.323103
**3**	lip	-21.175886	1.019469	22.195354	rel_conc	WT	DccpA	3	3	-4.972908
**4**	ndk	-4.678865	1.011832	5.690696	rel_conc	WT	DccpA	3	3	-8.529532

If you do not want to have the WT in the final figure run the cell below (optional)

In [13]:

```
#To run this cell Remove the #
  #final_df <- filter(final_df,Sample != "WT")
```

To plot

In [15]:

```
#To have the signif label as numerical use label = "p.adj" or "p"
#To have is as * or ns use label="p.adj.signif" or "p.signif"

  p<- ggbarplot(
    final_df, x = "Primers.x", y = "rel_conc", fill = "Sample",stat="identity",
    add = c("mean_sd","point"), palette = "Blues",position = position_dodge(0.8)) +
    stat_pvalue_manual(stat.test, label= "p.signif",tip.length = 0.01,hide.ns = F)+
    scale_y_continuous(expand = expansion(mult=c(0.01,0.1)),name = "Expression relat
    theme(legend.position = 'right',axis.title.x=element_blank())


 p
```

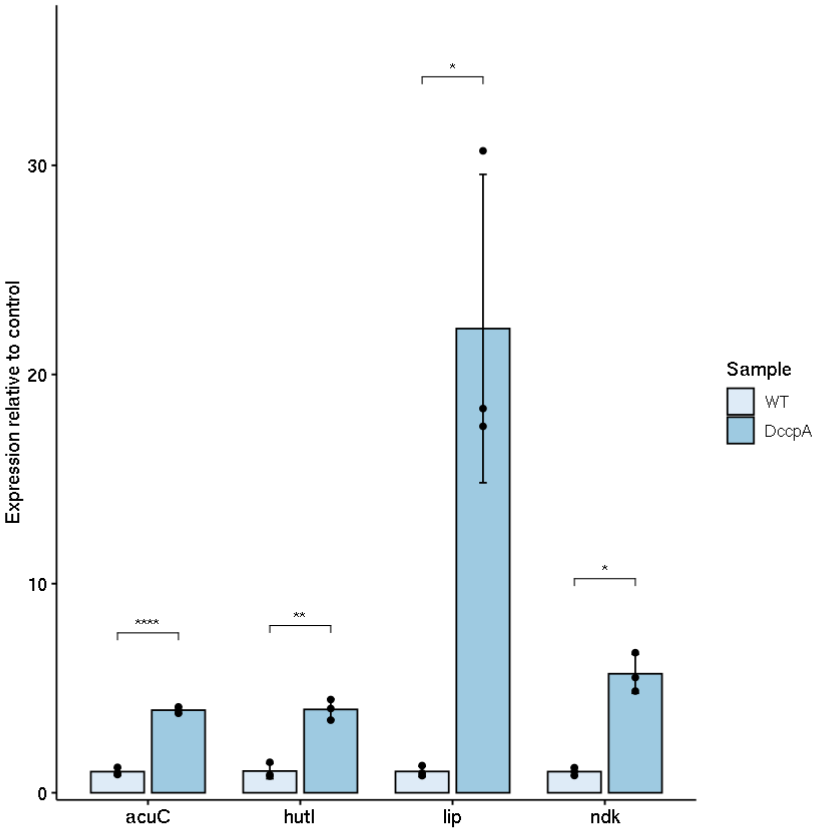

In [16]:

```
#Saving the graph change the name of the file
##You can also change the width and height to your needs
ggsave(p,filename = "211125_upregulated.pdf",width = 110, height = 140, units = "mm"
```

In [ ]:
